# Binary tree-inspired digital dendrimer

**DOI:** 10.1038/s41467-019-09957-6

**Published:** 2019-04-23

**Authors:** Zhihao Huang, Qiunan Shi, Jiang Guo, Fanying Meng, Yajie Zhang, Yutong Lu, Zhuangfei Qian, Xiaopeng Li, Nianchen Zhou, Zhengbiao Zhang, Xiulin Zhu

**Affiliations:** 1 College of Chemistry, Chemical Engineering and Materials Science, Soochow University, 215123 Suzhou, China; 2 Computer Science and Artificial Intelligence Laboratory, Massachusetts Institute of Technology, Cambridge, MA 02139 USA; 3 Department of Chemistry, University of South Florida, Tampa, FL 33620 USA; 4 Global Institute of Software Technology, No. 5. Qingshan Road, Suzhou National Hi-Tech District 215163 Suzhou, China

**Keywords:** Dendrimers, Polymer characterization

## Abstract

Digital polymers with precisely ordered units acting as the coded 0- or 1-bit, are introduced as a promising option for molecular data storage. However, the pursuit of better performance in terms of high storage capacity and useful functions never stops. Herein, we propose a concept of an information-coded 2D digital dendrimer. The divergent growth via thiol-maleimide Michael coupling allows precise arrangements of the 0- and 1-bits in the uniform dendrimers. A protocol for calculating the storage capacity of non-linear binary digital dendrimer is established based on data matrix barcode, generated by the tandem mass spectrometry decoding and encryption. Furthermore, the generated data matrix barcode can be read by a common hand-held device to cater the applications such as item identification, traceability and anticouterfeiting purpose. This work demonstrates the high data storage capacity of a uniform dendrimer and uncovers good opportunities for the digital polymers.

## Abstract (web summary)

Digital polymers with precisely ordered units for next-generation data storage media are continually investigated for higher storage capacity. Here the authors show the synthesis of information-coded 2D digital dendrimers and the generated data matrix barcode can be read by a common hand-held device.

## Introduction

**Fig. 1:**
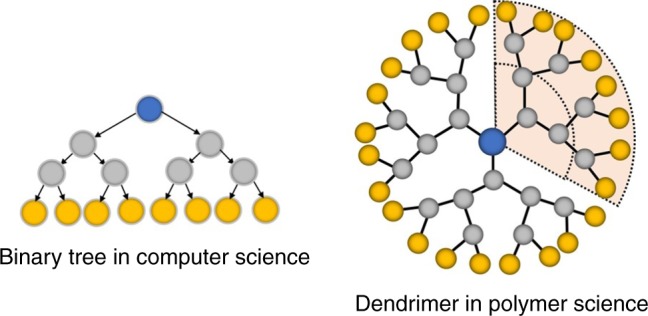
Binary tree versus dendrimer. A typical binary-tree data structure in computer science and a dendrimer or dendron (marked with pale yellow background) structure in polymer science. Both of them have highly branched structures which result multi pathways from periphery to focal (vice versa)

Binary tree in computer science is a data structure in which each node has at most two children^1^. Binary-tree-based data structures are widely used in computer science for efficient searching, labeling, and sorting values. Another important application of binary tree is information coding, such as Huffman coding^2^, used in lossless data compression, encryption and decryption. The basic principle of information coding is that the particular arrangement of nodes in tree structure is considered as the information. Interestingly, in polymer science, the dendron or dendrimer has the similar binary-tree-like structure (Fig. 1). Due to their globular and highly branched structures, the dendrimers usually convey interesting properties in comparison with their linear analogs, such as increased solubility, low intrinsic viscosity, etc.^3^. Many specific applications using dendrimers have thus been developed^3–19^.

Inspired by the 1→2 connectivity of binary-tree data structure, we synthesized dendrons and dendrimers with similar branching feature to explore their application in information coding with high storage capacity. Note that researchers have constantly been seeking for more efficient polymer system as data storage media; they range from natural polymer, i.e., DNA^20–23^, to linear synthetic polymer^24–36^, which is termed as digital polymer. Similar with the life information coding in DNA, the precise order of different monomer units along a digital polymer chain is regarded as the coded information. Owing to the inherent structure induced information coding, the digital polymers can be used as molecular barcodes or tags for anticounterfeiting purpose^37–39^. Considering its binary-tree like and highly branched structure, we envisioned that using dendrimer as another type of digital polymer^24,30,40,41^ might allow a high capability of information coding; that is, richer information can be reasonably decoded from a digital dendrimer. Moreover, intriguing applications of this digital dendrimer were also anticipated.

**Fig. 2:**
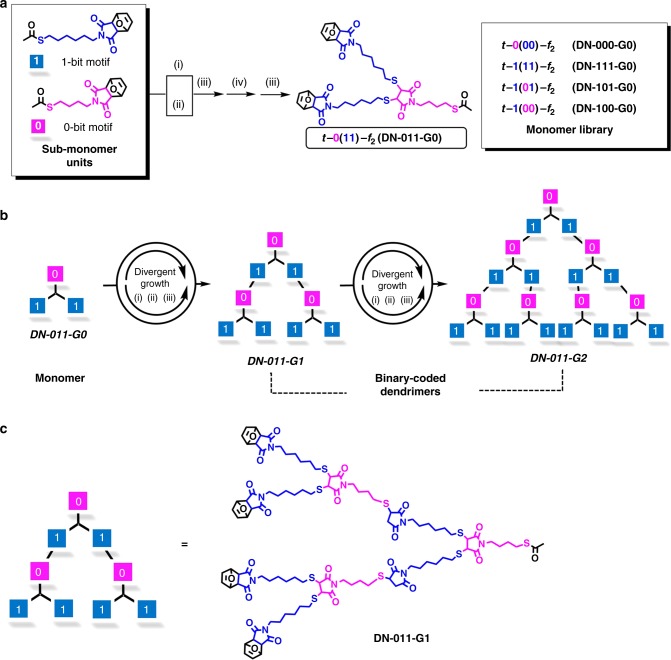
Synthesis of binary-coded digital dendrimers. **a** Synthesis of the monomers from the binary sub-monomer units (0- and 1-bit) based on orthogonal deprotections of thiol and maleimide (i and ii), thiol-maleimide Michael addition (iii) and maleimide ring regeneration maleimide ring (iv). **b** Construction of binary-coded dendrimers from monomers via divergent growth strategy (i, ii, and iii). Experimental conditions: (i) con. HCl, MeOH, 70 °C or acetyl chloride, MeOH/CH_2_Cl_2_ = 1:5, −78 to 25 °C, inert atmosphere, (ii) Toluene, 110 °C, (iii) Et_3_N, CHCl_3_, 25 °C, (iv) N-chlorosuccinimide, CCl_4_, 76 °C. **c** The molecular structure detail of the digital dendrimer (DN-011-G1) containing the certain binary information

In this work, digital dendrimers were precisely built by using binary-coded monomers via divergent strategy^42^, as illustrated in (Fig. 2a, b). The monomers were varied by assembling two different sub-monomer units (0- and 1-bit), endowing the binary codes and the structural diversity. Reliable decoding of these digital dendrimers was realized by using tandem mass spectrometry (MS/MS) authenticity^31,39^. The translation of the MS/MS decoded data created a readable data matrix barcode which was useful for item identification and anticounterfeiting tag.

## Results

### Design and synthesis of digital dendrimers

Information-coded digital polymers should meet two basic criteria, i.e., uniform chain length (monodisperse) and precisely-defined unit sequence. It is widely accepted that dendrimers are monodispersed and highly branched molecules, which meets the first criterion of digital polymer. Moreover, dendrimers are conventionally synthesized via stepwise divergent or convergent approach, offering the possibility to precisely arrange different monomers. In order to encode binary units into dendrimers, as depicted in Fig. 2a, two sub-monomer units containing a hexyl (1-bit) or a butyl group (0-bit) were designed (Supplementary Fig. 1). Then, a library of branched monomers coded by binary information, e.g., t-0(00)-f_2_, t-0(11)-f_2_, t-1(00)-f_2_, t-1(01)-f_2_, and t-1(11)-f_2_, (t presents as thiol group at the focal core, while f_*n*_ presents as furan group at the periphery terminal) was built (Supplementary Figs. 2–4 and 14–26, see experiment details in Supplementary Methods). By deliberately installing different monomers along the direction from focal to periphery, an array of binary digital dendrimers was constructed based on the highly efficient chemistry of orthogonal deprotections of thiol and maleimide, thiol-maleimide Michael addition (Supplementary Figs. 5–7, see experiment details in Supplementary Methods). For example, the dendron DN-011-G2 with the binary information was prepared by iteratively installing t-0(11)-f_2_ monomer (DN-011-G0) via the divergent dendrimer growth (Fig. 2b). Figure 2c displayed an example showing a digital dendrimer (DN-011-G1) containing the certain binary information. Using the same synthesis strategy, 12 digital dendrimers were precisely built as summarized in Table 1. These dendrimers were stringently characterized by thermal characterizations (Supplementary Figs. 9, 10), ^1^H NMR spectrometer (Supplementary Figs. 27–34 and 36–40), size exclusion chromatography (SEC) (Supplementary Fig. 35), and matrix-assisted laser desorption ionization time-of-flight mass spectrometry (MALDI-TOF MS) (Supplementary Figs. 41–43, 52–61). The precise constructions of these dendrimers indicated the successful coding of binary information as designed.

**Table 1:** Isolated yields and MALDI-TOF MS data of dendrimers

Dendrimers	*m*/*z*_calc._ (Da)	*m*/*z*_exp._ (Da)	Yield^a^ (%)	Error (Da)
DN-011-G1	1892.60 [M+Na-4Fu]^+^	1892.69	74.5	+0.09
DN-101-G1	1892.60 [M+Na-4Fu]^+^	1892.70	67.3	+0.10
DN-100-011-G1	1864.57 [M+Na-4Fu]^+^	1864.72	59.1	+0.15
DN-011-100-G1	1863.54 [M+Na-4Fu]^+^	1863.61	56.4	+0.07
DN-111-000-G1	1808.51 [M+Na-4Fu]^+^	1808.49	70.4	−0.02
DN-011-G2	4329.40 [M+Na-8Fu]^+^	4329.32	51.7	−0.08
DN-101-G2	4329.40 [M+Na-8Fu]^+^	4329.34	40.2	−0.07
DN-011-110-011-G2	4273.34 [M+Na-8Fu]^+^	4272.96	40.0	−0.38
DR-011-G1	1599.49 [M+Na-4Fu]^+^	1599.85	67.3	+0.36
DR-011-111-G1	1627.52 [M+Na-4Fu]^+^	1627.95	76.9	+0.43
DR-011-G2	4036.29 [M+Na-8Fu]^+^	4036.58	57.1	+0.29
DR-011-111-G2	4064.32 [M+Na-8Fu]^+^	4064.64	55.9	+0.32

Source data are provided as a Source Data file

^a^Isolated yield: purified by column chromatography over silica gel

### MS/MS decoding of digital dendrimers

**Fig. 3:**
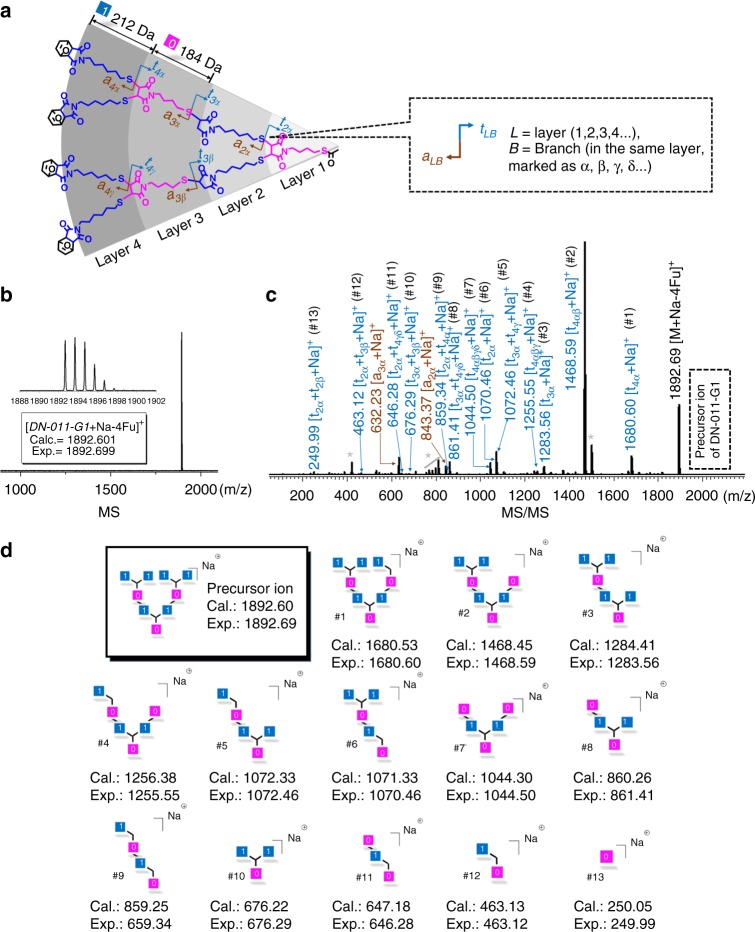
MS/MS decoding digital dendrimer DN-011-G1. **a** Illustration of the theoretical fragmentations. **b** MALDI-TOF MS spectrum. **c** MALDI-TOF MS/MS spectrum. *Signals come from secondary fragmentations. **d** All the main fragments were marked from #1 to #13. Source data are provided as a Source Data file

Besides the efficient data coding, reliable decoding is very important for a digital polymer as well. Tandem mass spectrometry (MS/MS) is regarded as an effective tool for decoding of both natural and synthetic polymers, especially for linear digital polymer**s**^26,43^. During MS/MS decoding, the polymer chains are fragmented into sequence-induced species. The mass/charge ratio analysis of the fragments enables the restoration of the sequence information^27^, thus realizes the information decoding. Up to date, the structure decoding of a non-linear digital polymer has rarely been explored. Here, MS/MS protocol was used to decode the binary information in the digital dendrimer. The 0-bit (butyl group, 184 Da) and 1-bit (hexyl group, 212 Da) coded sub-monomer units were designed with a succinimide thioether linkage as shown in Fig. 3a). Due to the relatively low bond dissociation energy (simulated: 57.16 kcal/mol) (Supplementary Fig. 8), the S-C bond tends to break during MS/MS with easily recognizable fragmentation pattern. All these dendrimers held an acetyl protected thiol moiety as the focal core (t) and furan protected maleimide as the peripheral terminal (f) (Fig. 3a). The fragments holding the f end-group were named as a_LB_ (all the furan groups were detached during MS/MS analysis), while the complementary fragments with t terminal were named as t_LB_^44,45^. Taking DN-011-G1 as an example, Fig. 3b, c showed its MS ([M+Na-4Fu]^+^, *m*/*z* *=* 1892.69 Da) and typical MS/MS pattern with detail fragmentation assignments, respectively. In Fig. 3c, the MS/MS signals could be well recognized and ascribed to the fragmented species. For example, three mono-protonated fragmented species were detected at *m*/*z* of 1680.59, 1283.56, and 1070.46 Da, which matched with the expected values of [t_4α_+Na]^+^, [t_3α_+Na]^+^, and [t_2α_+Na]^+^ (Fragmentation #1, 3 and 6 in Fig. 3c, d and Supplementary Table 1). Two complementary ions were found at *m*/*z* of 632.23 and 843.37 Da, corresponding to [a_3α_+Na]^+^ and [a_2α_+Na]^+^, respectively. Moreover, secondary fragmentation ions were also proposed in Supplementary Fig. 51. All the fragmentation species with calculated MS values were summarized in Fig. 3d, agreeing well with the experimental ones in Fig. 3c. These results confirmed that the MS/MS could induce useful fragmentation pattern of a dendrimer. However, due to its highly branched structure, the MS/MS technique cannot directly provide sequence information of the dendrimer like DN-011-G1, i.e., the spatial arrangements of the binary bits encoded in the dendrimer cannot be exactly decoded. This is much different from the linear digital polymer, which can be readily sequenced by using MS/MS decoding.

### MS/MS translation and storage capacity calculation

**Fig. 4:**
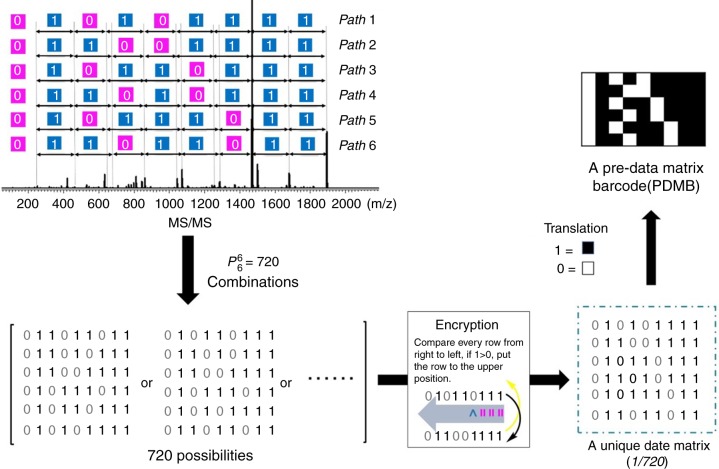
Translation from MS/MS fragmentation pattern of DN-011-G1 to data matrix barcode

Despite having difficulty in sequencing the dendrimer structure by MS/MS technique, it can afford a full sequence-related fragmentation pattern of a specific dendrimer (Fig. 3c, d). The question is how to translate the fragmentation pattern to the binary digital information and thus calculate the data storage capacity in a reasonable manner? Here, a similar manner for sequencing the linear polymer was used, wherein 1 binary path from one chain terminal to the other could be extracted from the fragmentation pattern. For the dendrimer as DN-011-G1, theoretically, there are 6 paths by orderly cleaving the 0- or 1-bit one by one from the periphery terminals to the focal core (see Supplementary Figs. 44–49). The analysis in Fig. 3c confirmed that the MS/MS decoding results agreed with the theoretical results, i.e., 6 binary paths, e.g., 011001111, 010101111, 010110111, etc, were found, as illustrated in Fig. 4a. It should be noted that each binary path is not the MS/MS induced chain cleavage route. Actually, the S-C bond cleavages during MS/MS decoding are random and nonselective in terms of its locations in the dendrimer. Since that the focal unit (0-bit) was connected with two adjacent 1-bits by two S-C bonds (Fig. 3a), the fragmentation of the focal 0-bit from DN-011-G1 cannot provide useful information for sequencing. Therefore, the overall 6 binary paths represented the binary information encoded in of DN-011-G1. Treating each binary path as a row of a 0/1 data matrix, it was interesting that 720 possibilities of data matrix could be obtained with different arrangements of the binary row. Simply put, each data matrix differed from another by different locations of the binary row. In order to obtain a data matrix for a specific dendrimer, we introduced the encryption cryptography^46,47^ in this study. As a matter of fact, the rule of encryption can be designed and varied on the demand for confidentiality. Here, the encryption rule of “1 > 0, right to left, upper” was applied. It compared the bits of different rows one by one from right to left. Specifically, if 1-bit was located in the right position, it was applied to the upper row. By using this encryption, 720 or even more possibilities of data matrix could be encrypted into one unique matrix for a specific dendrimer (Fig. 4 and Supplementary Fig. 50). As such, by MS/MS decoding and encryption, the full sequence-related fragmentation pattern can be translated to binary digital information via the data matrix.

**Fig. 5:**
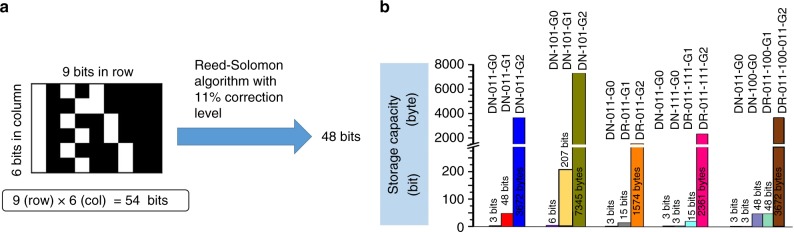
Storage capacity of the digital dendrimers. **a** Calculation of the storage capacity of digital dendrimer DN-011-G1 via corresponding data matrix barcode with correction level of 11%. **b** Summary of the storage capacities of the digital dendrimers

The data storage capacity is a key parameter for a digital polymer. Obviously, the calculation protocol of the storage capacity of a digital dendrimer differed much from the linear digital polymer. Herein, a protocol for calculating the storage capacity of a uniform binary-coded dendrimer was proposed by using a 2D data matrix barcode with the ever developed rule for calculating storage capacity^48^ (https://en.wikipedia.org/wiki/Data_Matrix). Taking DN-011-G1 as the example, the generated specific data matrix by summing and encrypting (Fig. 4) these 6 binary paths, can be translated into a pre-data matrix barcode (PDMB) by replacing 1-bit with a black module and 0-bit with a white module. To facilitate the building of PDMB, especially for digital dendrimers with complex structures, a binary-tree-based computational algorithm consisting of ExtractPath and TraverseTree was developed for the generation of PDMB (Supplementary Fig. 11). With this powerful binary-tree-based computation, a PDMB library was readily constructed including 5 monomers and 12 dendrimers. Each simulated PDMB perfectly matched with that by MS/MS decoding and encryption process. These PDMBs allowed the information storage capacity of a digital dendrimer to be calculated by considering the number of the modules and 11% normal correction level^48^ (Fig. 5a). The storage capacity of each DN-011-G1 molecule was calculated to be 48 bits. Note that the storage capacity of a PDMB strongly relies on the structural factors of a dendrimer, such as generation and monomer structure. As a dendrimer grows, the storage capacity significantly increases with more binary codes. For instance, the storage capacity increased 612 times from 48 bits of 1st generation DN-011-G1 to 3672 bytes of 2nd generation DN-011-G2 (Fig. 5b). In addition, due to different fragmentation patterns upon MS/MS decoding, dendrimers with different monomer structures might also cause different storage capacities. For example, in contrast to the 48 bits-coded DN-011-G1 from monomer t-0(11)-f_2_, each DN-101-G1 molecule could code for 207 bits of information. The dendrimer DR-011-111-G2 from t-0(11)-f_2_ and t-1(11)-f_2_ could code for 2361 bytes of information; however, the dendrimer DR-011-G2 from t-0(11)-f_2_ (Fig. 5b) could only encode 1574 bytes per molecule. Finally, after equipping PDMB with finder patterns (blue and pink) and a dashed pattern (green), the coded specific information in the data matrix barcode, such as chemical information or URL (Universal Resource Locator) (Supplementary Fig. 12), can be extracted with a smartphone. Thus, it can be used like a commercial data matrix barcode for product identification and traceability (Supplementary movie 1). Importantly and uniquely, the generated PDMB stemmed from a specific dendrimer could be used as an anticounterfeiting label (Supplementary Figs. 13, 62).

## Discussion

Inspired by the binary-tree data structure in computer science, this work married the dendrimer with information storage, and put forward the concept of digital dendrimer for information coding at a molecular level. Through MS/MS decoding and encryption, the coded binary data of a dendrimer was translated to a readable data matrix barcode. Based on the data matrix barcode, a protocol for calculating the storage capacity of non-linear binary digital dendrimer was established. The results demonstrated that the digital dendrimer had the high information storage capacity. This data codeable/decodable digital dendrimer can be used for item identification with powerful anticounterfeiting abilities. This work opens up a horizon of dendrimer with promising applications.

## Methods

### General synthetic protocol for digital dendrimer

DN-011-G0-MA: DN-011-G0 (4.0 g, 5.1 mmol) and 150 mL of toluene were added to a three-neck round-bottom flask equipped with a condenser. The mixture was stirred and refluxed at 110 °C under argon flow for about 10 h. ^1^H NMR showed that the reaction was complete. After cooling to room temperature, toluene was evaporated under vacuum. And the residue was dried under vacuum at 25 °C for 24 h to afford DN-011-G0-MA (3.1 g, yield 95%) as a pale yellow oil. ^1^H NMR (300 MHz, CDCl_3_, ppm): δ 6.74 (s, 4 H), 3.50 (m, 8 H), 2.97–2.63 (m, 6 H), 2.34 (s, 3 H), 1.43 (m, 20 H).

DN-011-G0-SH: DN-011-G0 (4.0 g, 5.1 mmol) was dissolved in a mix solvent of 80 mL of anhydrous MeOH and 400 mL of DCM in a 1.0-L three-neck round-bottom flask equipped with a 25-mL slow-addition apparatus under argon atmosphere. The mixture was cooled to −78 °C. Then acetyl chloride (17.1 mL, 0.24 mol) was added dropwise to the solution. The mixture was warmed to room temperature and stirred for 15 h. ^1^H NMR showed that the reaction was complete. The mixture was evaporated and the residue was re-dissolved in 100 mL DCM. The organic layer was washed with washed with saturated NaHCO_3_ (aq.) (2 × 50 mL), 50 mL of water and dried with anhydrous Na_2_SO_4_. Then, DCM was concentrated under vacuum to afford DN-011-G0-SH (3.5 g, yield 93%) as a pale yellow oil. ^1^H NMR (300 MHz, CDCl_3_, ppm): δ 6.51 (s, 4 H), 5.26 (s, 4 H), 3.62–3.36 (m, 8 H), 2.92–2.67 (m, 8 H), 2.55 (dd, *J* = 14.5, 7.0 Hz, 2 H), 1.48 (m, 20 H).

DN-011-G0-MA (0.20 g, 0.3 mmol) and DN-011-G0-SH (0.67 g, 0.9 mmol) were dissolved in 45.0 mL of dry CHCl_3_ in a 100-mL three-neck round-bottom flask under argon atmosphere at 25 °C. Triethylamine (0.4 mL, 2.2 mmol) was add via a 1-mL syringe over 5 min. The mixture was stirred for about 18 h. ^1^H NMR indicated that the reaction was complete. The reaction mixture was washed with saturated NaHCO_3_ (aq.) (15.0 mL) and water (15.0 mL). The combined organic layer was dried with anhydrous Na_2_SO_4_ and the solvent was evaporated to afford the crude product which was purified by column chromatography on silica gel eluting with dichloromethane/methanol (100/1-80/1) to give the DN-011-G1 (0.48 g, yield 74.5%) as a yellow sticky oil. ^1^H NMR (300 MHz, CDCl_3_, ppm): δ 6.52 (s, 8 H), 5.26 (s, 8 H), 3.72 (dd, *J* = 8.9, 3.3 Hz, 2 H), 3.63–3.36 (m, 24 H), 3.13 (dd, *J* = 18.6, 9.0 Hz, 2 H), 3.01–2.63 (m, 26 H), 2.55–2.41 (m, 2 H), 2.32 (s, 3 H), 1.80–1.18 (m, 60 H). ^13^C NMR (75 MHz, CDCl_3_, ppm) δ 195.54, 176.56, 176.27, 174.72, 174.59, 174.52, 136.52, 80.88, 47.35, 46.68, 38.70, 35.97, 31.98, 31.05, 30.63, 28.71, 28.35, 28.06, 27.31, 26.60, 26.46, 26.18, 26.01. MALDI-TOF MS for DN-011-G1, Calcd: *m/z* = 1892.60 of [M+Na-4Furan] ^+^; Found: 1892.69 of [M+Na-4Fu]^+^.

### Instrumentation

MALDI-TOF mass spectroscopy (MS) were acquired on an UltrafleXtreme MALDI-TOF mass spectrometer (Bruker Daltonics, Germany) equipped with an Nd:YAG smart beam-II laser with 355-nm wavelength and 200 Hz firing rate. For high-resolution mass analysis, the instrument was operated in the reflector mode. Tandem MALDI-TOF MS/MS analysis was recorded by using the LIFT mode on the same instrument controlled by the Flexcontrol 1.4 software package. For MS/MS, ions generated by the MALDI process were accelerated at 7.50 kV through a grid at 6.85 kV into a precursor ion selector (PCIS). In this region, the ions pass through a timed-ion-selector device that is able to select one parent ion from a mixture of ions at different *m*/*z* values for subsequent fragmentation in the LIFT cell. After the parent ion at a given *m*/*z* was selected by the timed-ion-selector, it passed through a retarding lens where the ions were decelerated and then passed into the LIFT cell. Fragmentation was performed in the simple metastable decomposition mode, and the fragments were further accelerated by 19 kV in the LIFT cell, passed through a post lift metastable suppressor (PLMS), into the reflector, and finally to the detector. MS and MS/MS data were further processed using FlexAnalysis 1.3 software package.

The compound trans-2-[3-(4-tert-butyl-phenyl)−2-methyl- 2-propenylidene]-malononitrile (DCTB, Aldrich, >98%) served as the matrix and was prepared in CHCl_3_ at a concentration of 20 mg/mL. The cationizing agent sodium trifluoroacetate was prepared in ethanol at a concentration of 10 mg/mL. The matrix and cationizing salt solutions were mixed in a ratio of 10/1 (v/v). The instrument was calibrated prior to each measurement with external PMMA at the molecular weight under consideration. All samples were dissolved in CHCl_3_ at a concentration of 10 mg/mL. After sample preparation and solvent evaporation, the target plate was inserted into the MALDI-TOF mass spectrometer.

## Supplementary information

Supplementary Information

Supplementary Movie 1

Description of Additional Supplementary Files

Source Data

## Data Availability

All relevant data are available within the paper and its Supplementary Information. The computational algorithm can be found at https://github.com/jiangfeng1124/digital-dendrimer. The source data underlying Table 1 and Fig. 3c are provided as a Source Data file. All other data are available from the authors upon request.

## Code availability

The computational algorithm can be found at https://github.com/jiangfeng1124/digital-dendrimer
