# Supplementary material for: Binary tree-inspired digital dendrimer
Source: Nat Commun. 2019 Apr 23;10:1918. doi: 10.1038/s41467-019-09957-6 (PMC6478934; doi:10.1038/s41467-019-09957-6)
Supplement: Supplementary file 1 — Supplementary Information [file 41467_2019_9957_MOESM1_ESM.pdf]

Supplementary Information

Binary Tree-Inspired Digital Dendrimer

Huang et al.

Supplementary Methods

Chemicals

Unless stated otherwise, commercially available reagents were purchased from Sigma-Aldrich, Acros Organic, Alfa Aesar, TCI, Energy chemical, Adams and Sinopharm Chem. Dry tetrahydrofuran (THF), dichloromethane (DCM), toluene and N,N- dimethylformamide (DMF) were collected fresh from an Innovative Technology PS-MD-5 solvent purification system. All other dry solvents used were dried over 4 Å molecular sieves and stored under argon.

Synthesis of sub-monomers (0-bit and 1-bit)

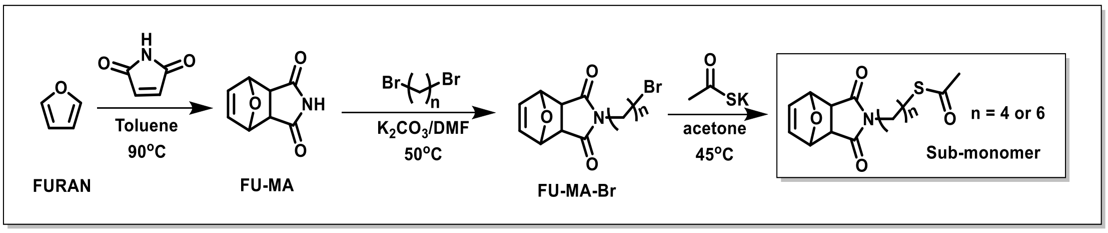

Supplementary Figure 1. Synthesis of sub-monomers**.**

FU-MA[1]**:** To a 5.0 L Parr stainless pressure reactor were added with maleimide (120.0 g, 1.24 mol) and furan (153.6 mL, 2.48 mol). The mixture was dissolved in 1600 mL of toluene. The vessel was sealed and heated at 90 °C for 12 h. After cooling the mixture to room temperature, the product precipitated as a white solid during the process. The mixture was filtered and the filter cake was washed with 3×150 mL cold toluene. The product was dried under vacuum at 25 °C overnight to afford **FU-MA** (169.6 g, yield 83.1%) as a white crystal. ^{1}H NMR (300 MHz, $CDCl_3$, ppm): δ 8.00 (s, 1H), 6.52 (s, 2H), 5.32 (d, J = 0.8 Hz, 2H), 2.89 (s, 2H).

FU-MA-Br (n = 4)[2]**:** **FU-MA** (5.0 g, 0.031 mol) was added to dry DMF (20.0 mL) in an oven-dried 100 mL three-necked round-bottom flask attached to a 50 mL slow-addition apparatus. Next, K_2CO_3 (8.6 g, 0.062 mol) was added to the mixture, the mixture was stirred

under argon flow and heated to 50 ºC for 1 h, then 1,4-dibromobutane (13.2 g, 0.062 mol) was added dropwise. The mixture was stirred at 50 ºC overnight. The mixture was cooled to room temperature and filtered. The filter cake was washed with 3×100 mL ethyl acetate (EA). Then the filtrate was combined and washed water 3×100 mL to remove DMF. The organic layer was then dried with anhydrous Na_2SO_4 and evaporated. The crude product was then purified by flash column chromatography on silica gel. Eluting with a mixed solvent of PE/EA (v/v = 2/1 to 1/1) to afford **FU-MA-Br (n = 4)** (8.0 g, yield 86.0%) as a pale yellow solid. ^{1}H NMR (300 MHz, $CDCl_3$, ppm): δ 6.51 (s, 2H), 5.26 (s, 2H), 3.51 (t, J = 6.7 Hz, 2H), 3.41 (t, J = 6.4 Hz, 2H), 2.85 (s, 2H), 2.01 – 1.49 (m, 4H)

Sub-monomer (n = 4): FU-MA-Br (n = 4) (8.0 g, 0.027 mol) was dissolved in 80 mL of dry acetone in a 100 mL round-bottom flask with a condenser. Then potassium thioacetate (4.6 g, 0.041 mol) was added. The mixture was heated to 45 ºC and kept under this temperature for 24 h. After cooling to room temperature, the mixture was filtered. The filter cake was washed with 3×50 mL acetone and the combined filtrate was concentrated. The residue was re-dissolved in 100 mL $CHCl_3$ and this solution was washed with 3×50 mL water, the organic phase was dried with anhydrous Na_2SO_4, and concentrated under vacuum to afford a deep yellow oil. The crude product was purified by flash column on silica gel. Eluting with a mixed solvent of PE/EA (v/v = 4/1 to 1/1) to afford **Sub-monomer (n=4)** (5.5 g, yield 84.1%) as a pale yellow solid. ^{1}H NMR (300 MHz, $CDCl_3$, ppm): δ 6.51 (s, 2H), 5.26 (s, 2H), 3.49 (t, J = 6.9 Hz, 2H), 2.95 – 2.72 (m, 4H), 2.31 (s, 3H), 1.70 – 1.47 (m, 4H).

The procedure of **FU-MA-Br (n = 6)** and **Sub-monomer (n = 6)** see the supporting information of our previous work.[3]

Synthesis of binary coded monomers

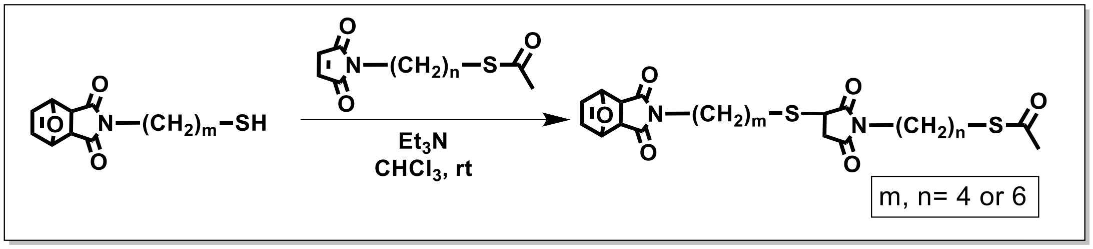

Supplementary Figure 2. Synthesis of the dimers**.**

C4-MA: Sub-monomer (n=4) (6.0 g, 20.3 mmol) and 60 mL of toluene were added to a three-neck flask equipped with a condenser. The mixture was stirred and refluxed at 110 ºC under argon flow for about 6 h. TLC showed the reaction was complete. After cooling to room temperature, toluene was evaporated under vacuum. And the residue was dried under vacuum at 25 ºC for 24 h to afford **C4-MA** (4.6 g, yield 98.7%) a pale yellow solid. ^{1}H NMR (300 MHz, $CDCl_3$, ppm): δ 6.70 (s, 2H), 3.53 (t, *J* = 6.9 Hz, 2H), 2.88 (t, *J* = 7.0 Hz, 2H), 2.42 – 2.18 (m, 4H), ^{13}C NMR (75 MHz, $CDCl_3$, ppm) δ 195.61, 170.74, 134.08, 37.21, 30.60, 28.39, 27.58, 26.78.

C4-SH: Sub-monomer (n=4) (0.5 g, 1.69 mmol) was dissolved in 14.6 mL of anhydrous methanol (MeOH) in a 25.0 mL three-neck round-bottom flask equipped with a condenser under argon atmosphere. The mixture was heated to 70 ºC. Then, concentrated HCl (0.64 mL, 7.69 mmol) was added dropwise to the solution *via* a 1.0 mL syringe. The mixture was refluxed at this condition for 2 h. TLC was used to monitor the reaction. After cooling to the room temperature, the mixture was quenched with 30.0 mL water and extracted with $CHCl_3$ (3×20 mL). The organic layer was combined and washed with 30 mL water and dried with anhydrous Na_2SO_4. $CHCl_3$ was concentrated under vacuum to afford **C4-SH** (0.42 g, yield 96.9%) a pale yellow oil. ^{1}H NMR (300 MHz, $CDCl_3$, ppm) δ 6.52 (s, 2H), 5.27 (s, 2H), 3.49 (t, *J* = 6.9 Hz, 2H), 2.84 (s, 2H), 2.53 (dd, *J* = 14.7, 6.9 Hz, 2H), 1.77 – 1.52 (m, 6H). ^{13}C NMR (75 MHz, $CDCl_3$, ppm) δ 176.24, 136.54, 80.93, 47.39, 38.25, 30.85, 26.22, 24.03.

00-dimer: C4-MA (4.23 g, 18.6 mmol) and **C4-SH** (4.50 g, 18.1 mmol) were dissolved in 60 mL dry $CHCl_3$ in a 100 mL three-neck round-bottom flask under argon atmosphere at 25 ºC. TEA (4.0 mL, 29.1 mmol) was added dropwise to the solution and the mixture was stirred for about 12 h.

The reaction mixture was quenched with 30 mL water and washed with 30 mL saturated $NaHCO_3$ (aq.). The combined organic layer was dried with anhydrous Na_2SO_4 and the solvent was evaporated to afford the crude product which was purified by column chromatography over silica gel eluting with PE/EA (1/1) to give the (7.4 g, yield 85.1%) as a colorless to pale yellow oil. ^{1}H NMR (300 MHz, $CDCl_3$, ppm) δ 6.52 (s, 2H), 5.26 (s, 2H), 3.70 (dd, *J* = 9.0, 3.6 Hz, 1H), 3.60 – 3.39 (m, 4H), 3.20 – 3.01 (m, 1H), 3.04 – 2.64 (m, 6H), 2.59 – 2.40 (m, 1H), 2.32 (s, 3H), 1.85 – 1.46 (m, 8H). ^{13}C NMR (75 MHz, $CDCl_3$, ppm) δ 195.62, 176.57, 176.26, 174.70, 136.52, 80.92, 47.39, 38.73, 38.31, 38.18, 35.99, 31.09, 30.62, 28.38, 26.74, 26.55, 25.95.

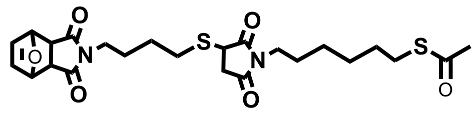

C4C6-dimer

The procedure is similar to C4-dimer mentioned above. **10-dimer** as a pale-yellow oil (isolated yield 84.3%). ^{1}H NMR (300 MHz, $CDCl_3$, ppm) δ 6.59 (s, 2H), 5.26 (s, 2H), 3.69 (dd, *J* = 9.0, 3.6 Hz, 1H), 3.57 – 3.41 (m, 4H), 3.11 (dd, *J* = 18.7, 9.0 Hz, 1H), 2.98 – 2.65 (m, 6H), 2.56 – 2.39 (m, 1H), 2.32 (s, 3H), 1.74 – 1.26 (m, 12H). ^{13}C NMR (75 MHz, $CDCl_3$, ppm) δ 195.92 , 176.62 , 176.26 , 174.76 , 136.53 , 80.93 , 47.39 , 38.86 , 38.72 , 38.20 , 36.00 , 31.11 , 30.64 , 29.27 , 28.90 , 28.15 , 27.35 , 26.56 , 26.16 , 25.98 .

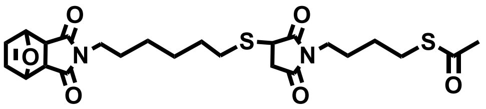

C6C4-dimer

01-dimer as a pale-yellow oil (isolated yield 86.7%). ^{1}H NMR (300 MHz, $CDCl_3$, ppm) δ 6.51 (d, *J* = 1.0 Hz, 2H), 5.26 (d, *J* = 1.0 Hz, 2H), 3.70 (dd, *J* = 9.0, 3.6 Hz, 1H), 3.49 (dt, *J* = 14.3, 7.0 Hz, 4H), 3.12 (dd, *J* = 18.7, 9.0 Hz, 1H), 3.00 – 2.64 (m, 6H), 2.49 (dd, *J* = 18.7, 3.6 Hz, 1H), 2.32 (s, 3H), 1.73 – 1.06 (m, 12H). ^{13}C NMR (75 MHz, $CDCl_3$, ppm) δ 195.65 , 176.61 , 176.29 , 174.76 , 136.54 , 80.91 , 47.39 , 38.87 , 38.77 , 38.33 , 36.03 , 31.58 , 30.63 , 28.69 , 28.40 , 28.11 , 27.33 , 26.76 , 26.59 , 26.08 .

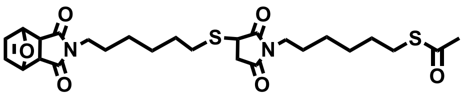

C6-dimer

11-dimer as a pale-yellow oil (isolated yield 84.9%) .^{1}H NMR (300 MHz, $CDCl_3$, ppm) δ 6.52 (s, 2H), 5.26 (s, 2H), 3.69 (dt, *J* = 10.1, 5.1 Hz, 1H), 3.48 (q, *J* = 7.3 Hz, 4H), 3.12 (dd, *J* = 18.6, 9.0 Hz, 1H), 2.97 – 2.78 (m, 5H), 2.72 (dt, *J* = 12.5, 7.4 Hz, 1H), 2.49 (dd, *J* = 18.6, 3.5 Hz, 1H), 2.32 (s, 3H), 1.72 – 1.47 (m, 8H), 1.47 – 1.16 (m, 8H). ^{13}C NMR (75 MHz,

$CDCl_3$, ppm) δ 195.85, 176.63, 176.25, 174.79, 136.52, 80.88, 47.36, 38.87, 38.81, 38.72, 36.02, 31.51, 30.63, 29.26, 28.88, 28.68, 28.13, 28.08, 27.34, 27.30, 26.14, 26.05.

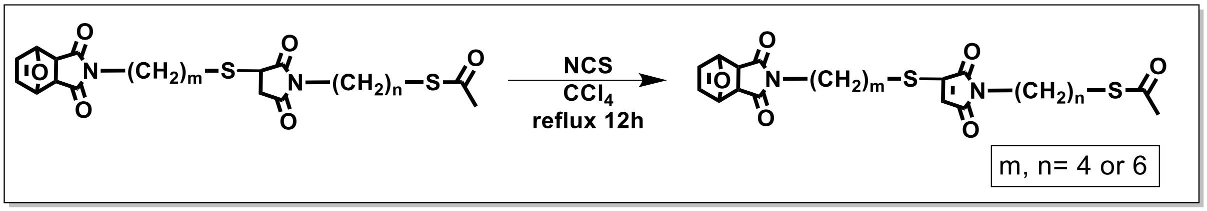

Supplementary Figure 3. Regenerate the double bone of the maleimide**.**

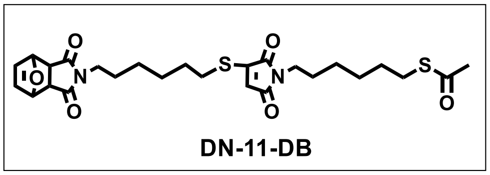

DN-11-DB: 11-dimer (2.0 g, 3.7 mmol) and carbon tetrachloride (100 mL) were added to a three-neck flask equipped with a condenser. Then, N-chlorosuccinimide (0.6 g, 4.4 mmol) was added in one portion to the stirring solution. The mixture was allowed to reflux at 80 ºC under argon atmosphere for about 12 h. TLC showed that the reaction was complete. After cooling to room temperature, the reaction mixture was concentrated. The residue was re-dissolved in 50.0 mL of dichloromethane and washed with saturated $NaHCO_3$ (aq.) (20.0 mL) and water (20.0 mL). The combined organic layer was dried with anhydrous Na_2SO_4 and the solvent was evaporated to afford the crude product which was purified by column chromatography on silica gel eluting with PE/EA (1/1) to give **DN-11-DB** (1.3 g, yield 66.0%) as a pale yellow solid ^{1}H NMR (300 MHz, $CDCl_3$, ppm): δ 6.52 (s, 2H), 6.02 (s, 1H), 5.26 (s, 2H), 3.48 (dd, J = 10.2, 3.9 Hz, 4H), 2.95 – 2.78 (m, 6H), 2.32 (s, 3H), 1.65 –1.17 (m, 16H). It should be mentioned that the main by-product is de-furan of the target molecule which can convert to the target by react with furan again.[3] Therefore, the target product can be produced in high yield.

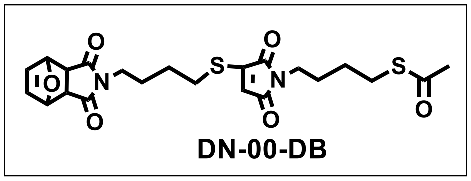

The procedure is similar to DN-11-DB mentioned above. **DN-00-DB** as a pale-yellow solid (isolated yield 55.1%). ^{1}H NMR (300 MHz, $CDCl_3$, ppm) δ 6.52 (d, J = 1.0 Hz, 2H), 6.04 (s, 1H), 5.26 (d, J = 1.0 Hz, 2H), 3.52 (dt, J = 10.6, 6.5 Hz, 4H), 2.99 – 2.75 (m, 6H), 2.32 (s, 3H), 1.90 – 1.46 (m, 8H). ^{13}C NMR (75 MHz, $CDCl_3$, ppm) δ 195.69 , 176.28 , 169.58 , 167.91 , 150.94 , 136.51 , 117.44 , 80.97 , 47.40 , 37.87 , 37.52 , 30.96 , 30.62 , 28.44 , 27.63 , 26.73, 24.64 .

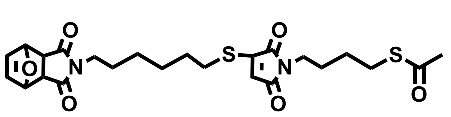

DN-01-DB

DN-01-DB as a pale-yellow solid (isolated yield 58.4%). ^{1}H NMR (300 MHz, $CDCl_3$, ppm) δ 6.52 (s, 2H), 6.02 (s, 1H), 5.26 (s, 2H), 3.50 (q, J = 7.1 Hz, 4H), 3.02 – 2.66 (m, 6H), 2.32 (s, 3H), 1.81 – 1.14 (m, 12H). ^{13}C NMR (75 MHz, $CDCl_3$, ppm) δ 195.73 , 176.33 , 169.67 , 167.97 , 151.44 , 136.54 , 117.20 , 80.95 , 47.39 , 38.64 , 37.52 , 31.57 , 30.63 , 28.45 , 28.20 , 27.64 , 27.45 , 27.24 , 26.78 , 25.88 .

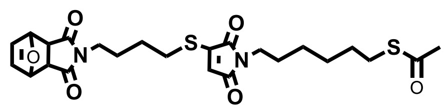

DN-10-DB

DN-10-DB as a pale-yellow solid (isolated yield 53.9%). ^{1}H NMR (300 MHz, $CDCl_3$, ppm) δ 6.52 (s, 2H), 6.04 (s, 1H), 5.26 (s, 2H), 3.51 (dt, J = 17.5, 6.7 Hz, 4H), 2.92– 2.74 (m, 6H), 2.32 (s, 3H), 1.81 – 1.65 (m, 4H), 1.64 – 1.45 (m, 4H), 1.33 (ddd, J = 19.7, 9.8, 4.5 Hz, 4H). ^{13}C NMR (75 MHz, $CDCl_3$, ppm) δ 195.97 , 176.30 , 169.71 , 168.00 , 150.88 , 136.52 , 117.45 , 80.97 , 47.40 , 38.03 , 37.88 , 30.94 , 30.64 , 29.32 , 28.99 , 28.38 , 28.24 , 26.71 , 26.22 , 24.66 .

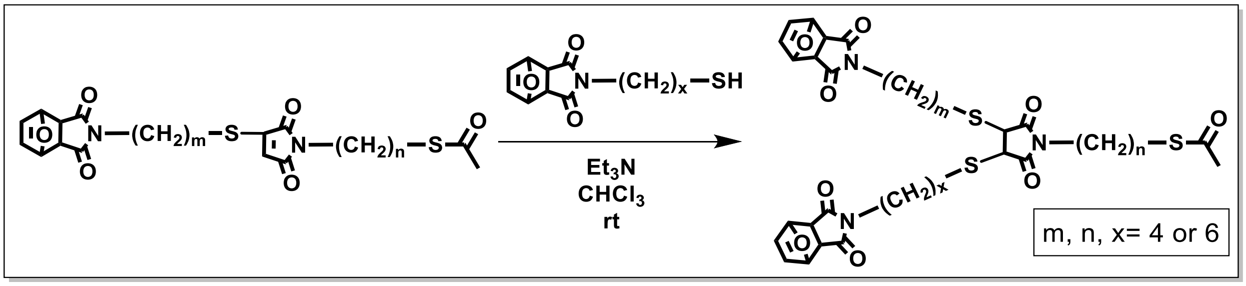

Supplementary Figure 4. Synthesis of the binary coded monomers

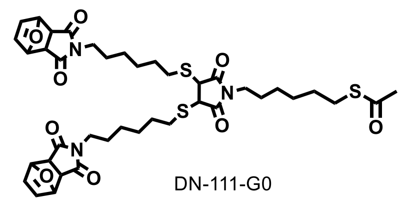

DN-111-G0

DN-111-G0: DN-11-DB (4.0 g, 7.5 mmol) and **C6-SH** (2.1 g, 7.3 mmol) were dissolved in 45.0 mL of dry $CHCl_3$ in a 100 mL three-neck round-bottom flask under argon atmosphere at 25 °C. TEA (1.8 mL, 10.0 mmol) was added *via* a 2 mL syringe over 5 min. The mixture was stirred for about 12 h. ^{1}H NMR indicated that the reaction was complete. The reaction mixture was washed with saturated $NaHCO_3$ (aq.) (20.0 mL) and water (20.0 mL). The combined organic layer was dried with anhydrous Na_2SO_4 and the solvent was evaporated to afford the crude product, which was purified by column chromatography over silica gel eluting with PE/EA (5/1-1/2) to give the **DN-111-G0** (3.9 g, yield 65.0%) as a yellow oil. ^{1}H

NMR (300 MHz, $CDCl_3$, ppm): δ 6.51 (s, 4H), 5.26 (s, 4H), 3.61 – 3.31 (m, 8H), 2.96 – 2.68 (m, 10H), 2.32 (s, 3H), 1.71 – 1.47 (m, 12H), 1.44 – 1.25 (m, 12H). ^{13}C NMR (75 MHz, $CDCl_3$, ppm): δ 195.86, 176.25, 174.60, 136.52, 80.89, 47.37, 46.72, 39.04, 38.75, 31.97, 30.63, 29.28, 28.92, 28.75, 28.16, 28.11, 27.35, 27.24, 26.07. MALDI-TOF for **DN-111**, Calcd: *m/z* = 702.23 $[M + Na\text{-}2Fu]^+$; Found: 702.29 $[M + Na\text{-}2Fu]^+$.

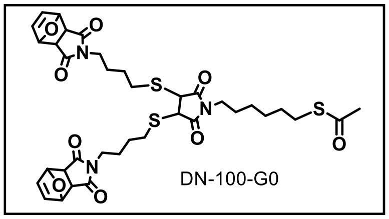

The procedure is similar to **DN-111-G0** mentioned above. **DN-100-G0** as a yellow oil (isolated yield 63.5%). ^1H NMR (300 MHz, $CDCl_3$, ppm) δ 6.52 (s, 4H), 5.27 (s, 4H), 3.51 (t, *J* = 6.7 Hz, 6H), 3.43 (s, 2H), 2.93 – 2.57 (m, 10H), 2.32 (s, 3H), 2.07 – 1.03 (m, 16H). ^{13}C NMR (75 MHz, $CDCl_3$, ppm) δ 195.90 , 176.25 , 174.51 , 136.53 , 80.93 , 47.41 , 46.55 , 38.15 , 31.50 , 30.64 , 29.28 , 28.93 , 28.15 , 27.22 , 26.53 , 26.08 , 25.99. MALDI-TOF for **DN-100-G0,** Calcd: *m/z* = 646.17 $[M + Na\text{-}2Fu]^+$; Found: 646.23 $[M + Na\text{-}2Fu]^+$,

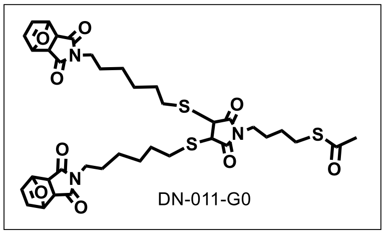

DN-011-G0 as a yellow oil (isolated yield 67.2%). ^1H NMR (300 MHz, $CDCl_3$, ppm) δ 6.51 (s, 4H), 5.26 (s, 4H), 3.72 – 3.28 (m, 8H), 3.01 – 2.62 (m, 10H), 2.32 (s, 3H), 1.83 – 1.08 (m, 20H). ^{13}C NMR (75 MHz, $CDCl_3$, ppm) δ 195.58 , 176.28 , 174.58 , 136.54 , 80.91 , 47.39 , 46.69 , 38.77 , 38.50 , 32.00 , 30.63 , 28.74 , 28.39 , 28.11 , 27.36 , 26.63 , 26.49 , 26.07. MALDI-TOF for **DN-011-G0**, Calcd: *m/z* = 674.20 $[M + Na\text{-}2Fu]^+$; Found: 674.30 $[M + Na\text{-}2Fu]^+$,

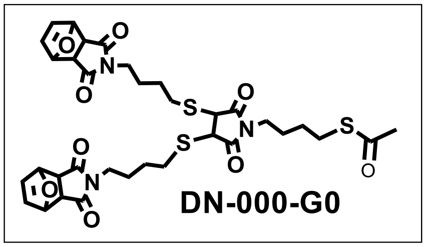

DN-000-G0 as a yellow oil (isolated yield 51.0%). ^1H NMR (300 MHz, $CDCl_3$, ppm) δ 6.52 (s, 4H), 5.27 (s, 4H), 3.51 (t, *J* = 6.4 Hz, 6H), 3.43 (s, 2H), 3.07 – 2.62 (m, 10H), 2.32 (s, 3H), 1.64 (m, 12H). ^{13}C NMR (75 MHz, $CDCl_3$, ppm) δ 195.60 , 176.25 , 174.46 , 136.52 , 80.93 , 47.41 , 46.52 , 38.55 , 38.15 , 31.51 , 30.64 , 28.38 , 26.63 , 26.52 , 25.98. **DN-000-G0**, Calcd: *m/z* = 618.14 $[M + Na\text{-}2Fu]^+$; Found: 618.21 $[M + Na\text{-}2Fu]^+$

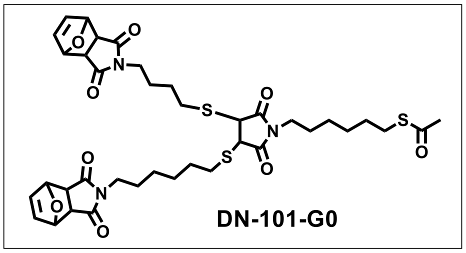

DN-101-G0 as a yellow oil (isolated yield 63.3%). ^{1}H NMR (300 MHz, $CDCl_3$, ppm) δ 6.51 (s, 4H), 5.26 (s, 4H), 3.60 – 3.27 (m, 8H), 2.96 – 2.63 (m, 10H), 2.32 (s, 3H), 1.60 – 1.19 (m, 20H). ^{13}C NMR (75 MHz, $CDCl_3$, ppm) δ 195.90 , 176.26 , 174.57 , 136.54 , 80.91 , 47.39 , 46.71 , 46.55 , 39.07 , 38.77 , 38.15 , 31.98 , 31.51 , 30.64 , 29.29 , 28.93 , 28.75 , 28.16 , 28.12 , 27.36 , 27.23 , 26.53 , 26.08 , 26.00. MALDI-TOF for **DN-101-G0**, Calcd: *m/z* = 674.20 $[M + Na\text{-}2Fu]^{+}$; Found: 674.18 $[M + Na\text{-}2Fu]^{+}$

Synthesis of Digital dendrons and dendrimers (G1-G2)

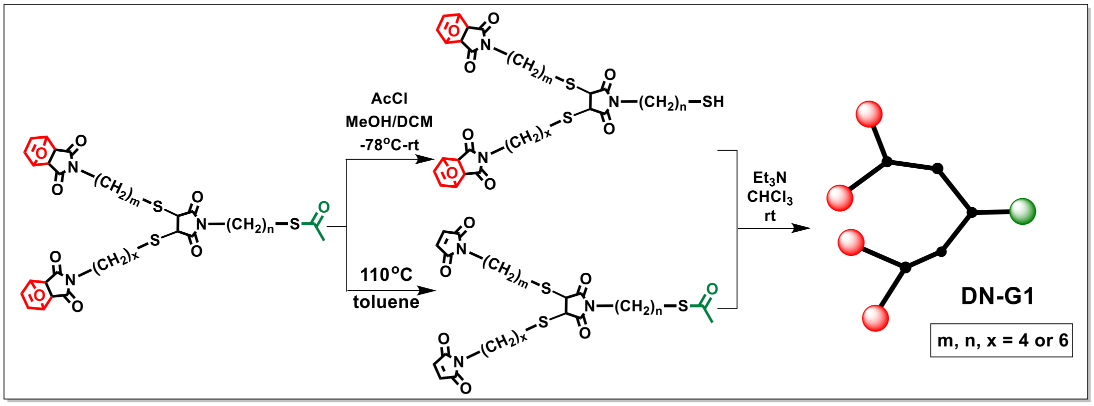

Supplementary Figure 5. The general divergent strategy used to synthesis of the digital dendrons (G1)

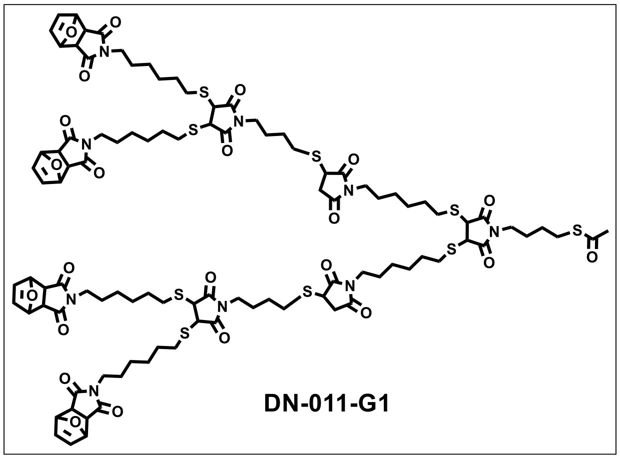

DN-011-G1: DN-011-G0-MA and **DN-011-G0-SH** precursors to **DN-011-G1** were prepared using the procedures described in the literature.[3]。

DN-011-G0-MA (0.20 g, 0.3 mmol) and **DN-011-G0-SH** (0.67 g, 0.9 mmol) were dissolved in 45.0 mL of dry $CHCl_3$ in a 100 mL three-neck round-bottom flask under argon atmosphere at

25 ºC. TEA (0.4 mL, 2.2 mmol) was add *via* a 1 mL syringe over 5 min. The mixture was stirred for about 18 h. ^{1}H NMR indicated that the reaction was complete. The reaction mixture was washed with saturated $NaHCO_3$ (aq.) (15.0 mL) and water (15.0 mL). The combined organic layer was dried with anhydrous Na_2SO_4 and the solvent was evaporated to afford the crude product which was purified by column chromatography on silica gel eluting with DCM/MeOH (100/1-80/1) to give the **DN-011-G1** (0.48 g, yield 74.5%) as a yellow sticky oil. ^{1}H NMR (300 MHz, $CDCl_3$, ppm): δ 6.52 (s, 8H), 5.26 (s, 8H), 3.72 (dd, *J* = 8.9, 3.3 Hz, 2H), 3.63 – 3.36 (m, 24H), 3.13 (dd, *J* = 18.6, 9.0 Hz, 2H), 3.01 – 2.63 (m, 26H), 2.55 – 2.41 (m, 2H), 2.32 (s, 3H), 1.80 – 1.18 (m, 60H). ^{13}C NMR (75 MHz, $CDCl_3$, ppm) δ 195.54 , 176.56 , 176.27 , 174.72 , 174.59 , 174.52 , 136.52 , 80.88 , 47.35 , 46.68 , 38.70 , 35.97 , 31.98 , 31.05 , 30.63 , 28.71 , 28.35 , 28.06 , 27.31 , 26.60 , 26.46 , 26.18 , 26.01. MALDI-TOF for **DN-011-G1**, Calcd: *m/z* = 1892.60 [M + Na-4Fu] $^{+}$; Found: 1892.69 [M + Na-4Fu] $^{+}$.

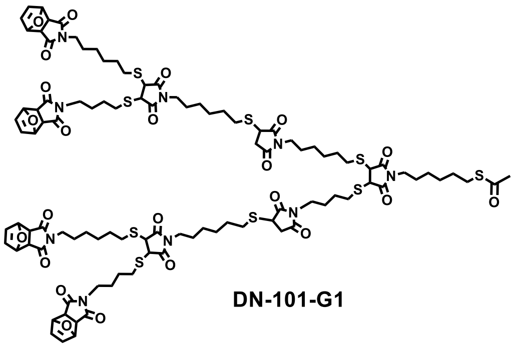

DN-101-G1

The procedure is similar to **DN-011-G1** mentioned above. **DN-101-G1** as a yellow sticky oil (isolated yield 67.3%). ^{1}H NMR (300 MHz, $CDCl_3$, ppm): δ 6.51 (s, 8H), 5.26 (s, 8H), 3.71 (dt, *J* = 9.0, 3.5 Hz, 2H), 3.65 – 3.20 (m, 24H), 3.13 (ddd, *J* = 18.6, 9.0, 4.2 Hz, 2H), 3.01 – 2.63 (m, 26H), 2.50 (ddd, *J* = 18.7, 3.7, 2.0 Hz, 2H), 2.05 (s, 3H), 1.80 – 1.08 (m, 60H). ^{13}C NMR (75 MHz, $CDCl_3$, ppm) δ 195.57, 176.56 ,176.28 , 174.81 , 174.62, 174.57, 136.53, 80.92, 47.39, 46.58, 38.76, 38.14, 31.99, 31.52, 30.66, 29.68, 29.29, 28.92, 28.75, 28.16, 28.12, 27.36 , 26.53 , 26.12, 26.08. MALDI-TOF for **DN-101-G1**, Calcd: *m/z* = 1892.60 [M + Na-4Fu] $^{+}$; Found: 1892.70 [M + Na-4Fu] $^{+}$.

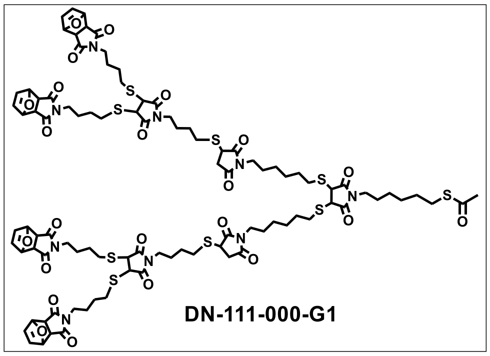

DN-111-000-G1 as a yellow sticky oil (isolated yield 70.4%). ^{1}H NMR (300 MHz, $CDCl_3$, ppm): δ 6.45 (s, 8H), 5.20 (s, 8H), 3.64 (dd, *J* = 8.2, 3.6 Hz, 2H), 3.56 – 3.26 (m, 24H), 3.06 (dd, *J* = 18.7, 8.1 Hz, 2H), 2.94 – 2.50 (m, 26H), 2.42 (d, *J* = 18.4, 3.6 Hz, 2H), 2.25 (s, 3H), 1.83 – 0.93 (m, 48H). ^{13}C NMR (75 MHz, $CDCl_3$, ppm) δ 194.88 , 175.57 , 175.24 , 173.73 , 173.59 , 173.50 , 135.53 , 79.93 , 46.41 , 45.68 , 45.53 , 37.81 , 37.50 , 37.14 , 35.02 , 31.02 , 30.53 , 30.10 , 29.66 , 28.67 , 28.30 , 27.93 , 27.80 , 27.17 , 26.38 , 26.25 , 25.51. MALDI-TOF for **DN-111-000-G1**, Calcd: *m/z* = 1808.51 $[M + Na\text{-}4Fu]^{+}$; Found: 1808.49 $[M + Na\text{-}4Fu]^{+}$

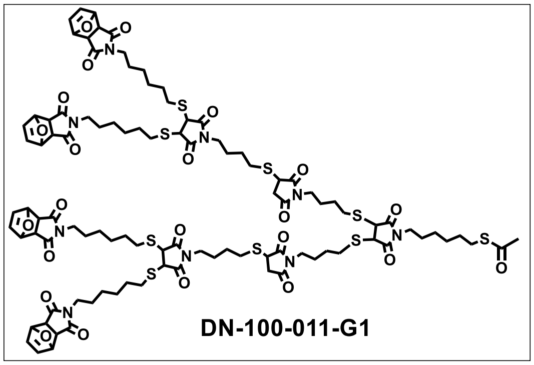

DN-100-011-G1 as a yellow sticky oil (isolated yield 59.1%). ^{1}H NMR (300 MHz, $CDCl_3$, ppm): δ 6.51 (s, 8H), 5.26 (s, 8H), 3.72 (dd, *J* = 9.0, 3.6 Hz, 2H), 3.59 – 3.34 (m, 24H), 3.14 (dd, *J* = 18.7, 9.1 Hz, 2H), 3.00 – 2.65 (m, 26H), 2.49 (dd, *J* = 18.6, 3.6 Hz, 2H), 2.32 (s, 3H), 1.99 – 1.08 (m, 56H). MALDI-TOF for **DN-100-011-G1**, Calcd: *m/z* = 1864.57 $[M + Na\text{-}4Fu]^{+}$; Found: 1864.72 $[M + Na\text{-}4Fu]^{+}$

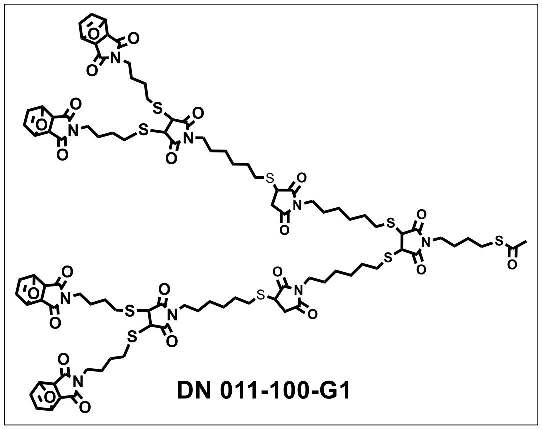

DN-011-100-G1 as a yellow sticky oil (isolated yield 56.4%). ^{1}H NMR (300 MHz, $CDCl_3$, ppm): δ 6.52 (s, 8H), 5.27 (s, 8H), 3.70 (dd, *J* = 9.0, 3.6 Hz, 2H), 3.58 – 3.34 (m, 24H), 3.13 (dd, *J* = 18.7, 9.0 Hz, 2H), 2.92 – 2.62 (m, 26H), 2.50 (dd, *J* = 18.6, 3.6 Hz, 2H), 2.32 (s, 3H), 1.87 – 1.06 (m, 48H). MALDI-TOF for **DN-011-100-G1**, Calcd: *m/z* = 1836.54 $[M + Na\text{-}4Fu]^{+}$; Found: 1836.61 $[M + Na\text{-}4Fu]^{+}$.

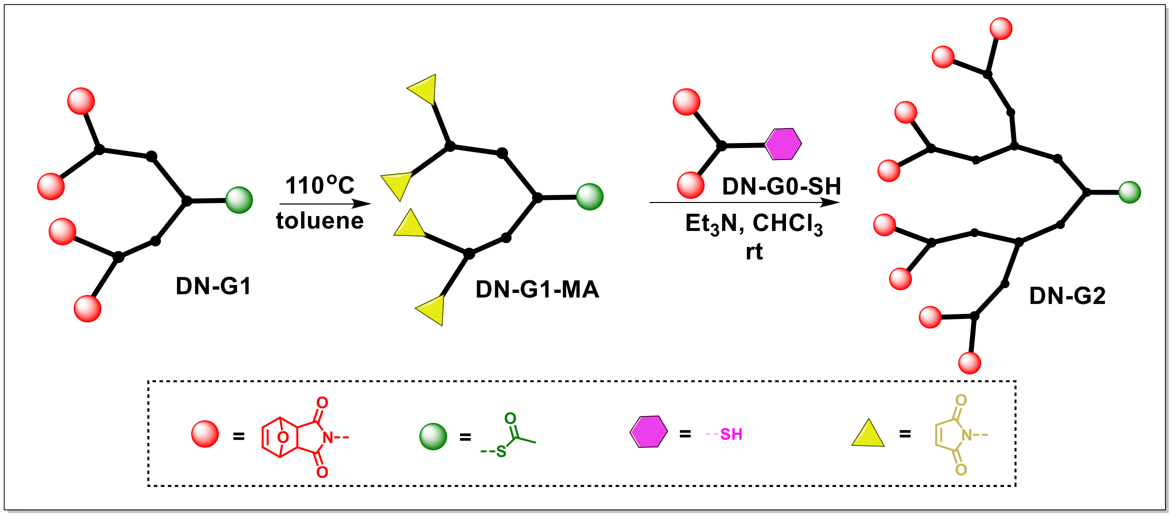

Supplementary Figure 6. The general divergent strategy used to synthesis of the digital dendrons (G2)

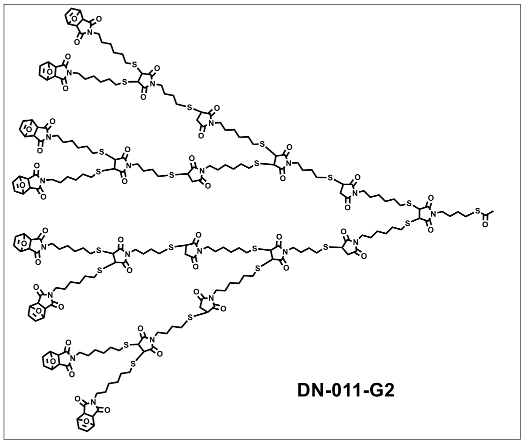

DN-011-G2: DN-011-G1-MA and **DN-011-G0-SH** precursors to **DN-011-G2** were prepared using the procedures described in the literature.[3]

DN-011-G1-MA (93.5mg, 0.05 mmol) and **DN-011-G0-SH** (233.5 mg, 0.30 mmol) were dissolved in 25.0 mL dry $CHCl_3$ in a 50 mL three-neck round-bottom flask under argon atmosphere at 25 °C. TEA (0.5 mL, 2.8 mmol) was add *via* a 1 mL syringe over 5 min. The mixture was stirred for about 48 h. ^1H NMR indicated the reaction was complete. The reaction mixture was washed with saturated $NaHCO_3$ (aq.) (15.0 mL) and water (15.0 mL). The combined organic layer was dried with anhydrous Na_2SO_4 and the solvent was evaporated to afford the crude product, which was purified by column chromatography on silica gel eluting with DCM/MeOH (80/1-30/1) to give the **DN-011-G2** (130.0 mg, yield 51.7%) as a yellow sticky oil. ^1H NMR (300 MHz, $CDCl_3$, ppm): δ 6.51 (s, 16H), 5.26 (s, 16H), 3.71 (dd, J = 8.9, 3.4 Hz, 6H), 3.65 – 3.38 (m, 56H), 3.12 (dd, J = 18.6, 9.0 Hz, 6H), 2.95 – 2.68 (m, 58H), 2.48 (dd, J = 18.6, 3.5 Hz, 6H), 2.32 (s, 3H), 1.82 – 0.79 (m, 140H). ^{13}C NMR (75 MHz, $CDCl_3$, ppm): δ 195.50, 176.68 , 176.62 , 176.32 ,174.84, 174.77 , 174.64 , 136.55 ,

80.91 , 47.39 , 46.69 , 46.28 , 39.06, 38.77 , 38.48 , 36.02 , 32.51 , 32.05 , 31.65 , 31.11 , 30.67 , 30.30 , 29.68 , 29.30 , 28.93 , 28.75 , 28.16,28.14, 27.36 , 26.48 , 26.25 , 26.07. MALDI-TOF for **DN-011-G2**, Calcd: *m/z* = 4329.40 $[M + Na\text{-}8Fu]^+$; Found: 4329.32 $[M + Na\text{-}8Fu]^+$.

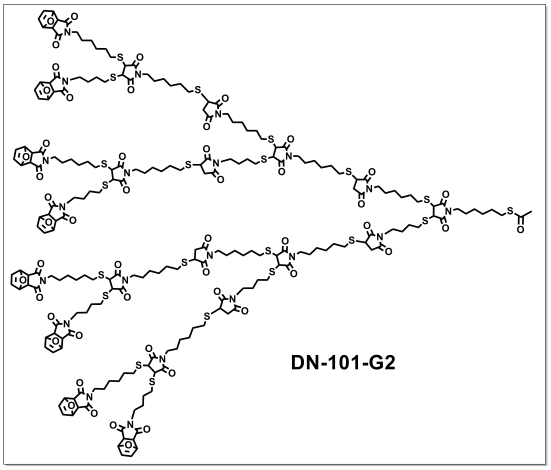

The procedure is similar to **DN-011-G2** mentioned above. **DN-101-G2** as a yellow sticky oil (isolated yield 40.2%). ^{1}H NMR (300 MHz, $CDCl_3$, ppm): δ 6.51 (s, 16H), 5.26 (s, 16H), 3.70 (dd, *J* = 8.8, 3.9 Hz, 6H), 3.56 – 3.30 (m, 56H), 3.13 (ddd, *J* = 18.8, 9.4, 4.3 Hz, 6H), 3.03 – 2.65 (m, 58H), 2.50 (d, *J* = 18.4 Hz, 6H), 2.32 (s, 3H), 1.83 – 1.14 (m, 140H). ^{13}C NMR (75 MHz, $CDCl_3$, ppm): δ 194.88, 175.64 , 175.26 , 173.79 , 173.55 , 135.52 , 79.91 , 46.39 , 45.72 , 45.57 , 38.03 , 37.90 , 37.76 , 37.14 , 35.05 , 31.00 , 30.63 , 30.51 , 29.66 , 29.30 , 28.68 , 28.30 , 28.13 , 27.74 , 27.13 , 26.36 , 26.21 , 25.53. MALDI-TOF for **DN-101-G2**, Calcd: *m/z* = 4329.40 $[M+Na\text{-}8Fu]^+$; Found: 4329.34 $[M+Na\text{-}8Fu]^+$.

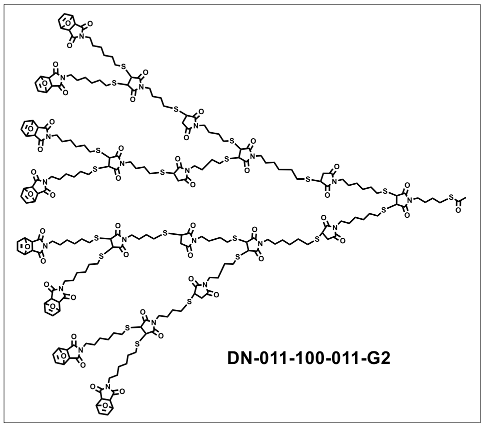

The procedure is similar to **DN-011-G2** mentioned above. **DN-011-100-011-G2** as a yellow sticky oil (isolated yield 40.0%). ^{1}H NMR (300 MHz, $CDCl_3$, ppm): δ 6.51 (s, 16H), 5.26 (s, 16H), 3.72 (dt, *J* = 7.4, 3.7 Hz, 6H), 3.64 – 3.33 (m, 56H), 3.14 (ddd, *J* = 18.7, 9.0, 3.2 Hz, 6H), 3.04 – 2.62 (m, 58H), 2.49 (dd, *J* = 18.7, 3.5 Hz, 6H), 2.32 (s, 3H), 1.79 – 1.24 (m, 132H). ^{13}C NMR (75 MHz, $CDCl_3$, ppm): δ 195.56, 176.63 , 176.57 , 174.80 , 174.70 , 174.60 , 174.47 , 136.53 , 80.90 , 47.38 , 46.70 , 46.28 , 39.09 , 38.79, 38.47 , 38.24 , 36.01 , 32.51 , 32.04 , 31.61 , 31.13 , 30.65 , 29.63, 29.30 , 28.10 , 27.35 , 27.20 , 26.55 , 26.49 , 26.06. MALDI-TOF for **DN-011-100-011-G2**, Calcd: *m/z* = 4273.34 $[M+Na\text{-}8Fu]^+$; Found: 4272.96 $[M+Na\text{-}8Fu]^+$.

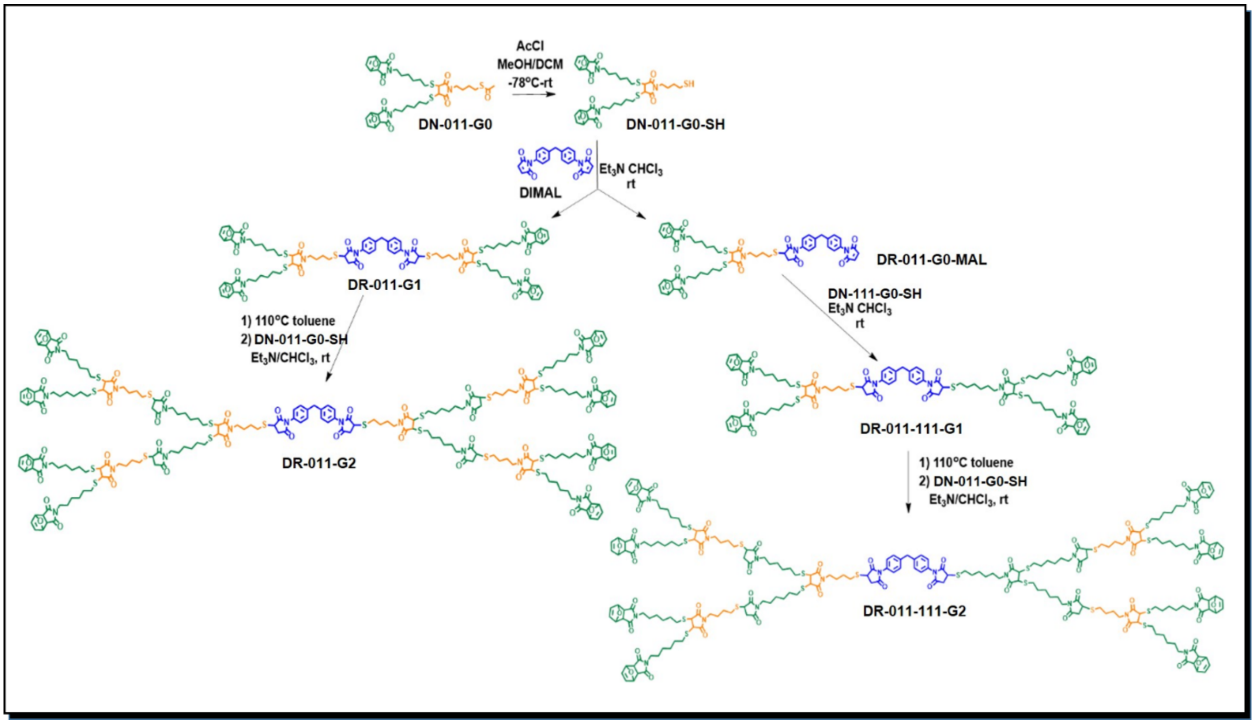

Supplementary Figure 7. The general divergent strategy used to synthesis of the digital dendrimers (G1 and G2)

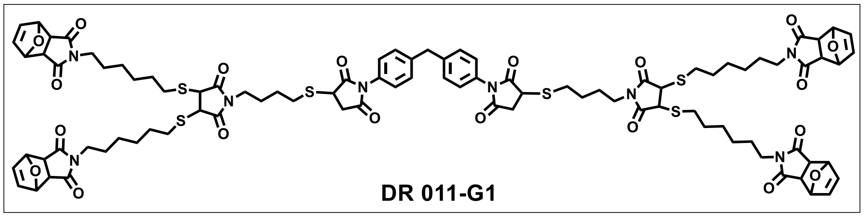

DR-011-G1: DN-011-G0-SH precursor to **DR-011-G1** was prepared using the procedures described in the literature.[3]

DIMAL (60.9 mg, 0.17 mmol) and **DN-011-G0-SH** (253.6 mg, 0.34 mmol) were dissolved in 20.0 mL dry $CHCl_3$ in a 50 mL three-neck round-bottom flask under argon atmosphere at 25 ºC. TEA (0.2 mL, 1.12 mmol) was add *via* a 1 mL syringe over 5 min. The mixture was stirred for about 12 h. ^{1}H NMR indicated the reaction was complete. The reaction mixture was washed with saturated $NaHCO_3$ (aq.) (15.0 mL) and water (15.0 mL). The combined organic layer was dried with anhydrous Na_2SO_4 and the solvent was evaporated to afford the crude product, which was purified by column chromatography on silica gel eluting with DCM/MeOH (100/1-80/1) to give the **DR-011-G1** (211.7 mg, yield 67.3%) as a yellow sticky oil. ^{1}H NMR (300 MHz, $CDCl_3$, ppm): δ 7.38 – 7.13 (m, 8H), 6.50 (s, 8H), 5.26 (s, 8H), 4.04 (s, 2H), 3.87 (dd, *J* = 8.6, 3.0 Hz, 2H), 3.59 – 3.39 (m, 16H), 3.30 (dd, *J* = 18.5, 9.2 Hz, 2H), 3.12 – 2.54 (m, 20H), 1.83 – 1.09 (m, 40H). ^{13}C NMR

(75 MHz, $CDCl_3$, ppm): δ 176.30 , 175.48 , 174.66 , 173.75 , 136.55 , 129.79 , 126.50 , 80.92 , 47.39 , 46.70 , 38.77 , 36.10 , 32.06 , 28.75 , 28.11 , 27.37 , 26.07. MALDI-TOF for **DR-011-G1,** Calcd: *m/z* = 1599.49 [M + Na-4Fu] $^+$; Found: 1599.85 [M + Na-4Fu] $^+$.

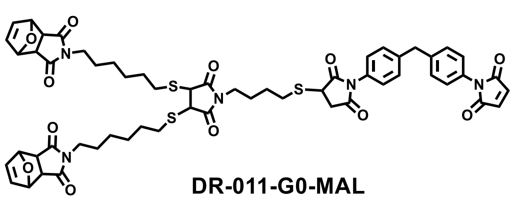

DR-011-G0-MAL: DIMAL (182.8 mg, 0.51 mmol) and **DN-011-G0-SH** (253.6 mg, 0.34 mmol) were dissolved in 25.0 mL dry $CHCl_3$ in a 50 mL three-neck round-bottom flask under argon atmosphere at 25 ºC. TEA (0.1 mL, 0.56 mmol) was add *via* a 1 mL syringe over 5 min. The mixture was stirred for about 12 h. ^{1}H NMR indicated the reaction was complete. The reaction mixture was washed with saturated $NaHCO_3$ (aq.) (15.0 mL) and water (15.0 mL). The combined organic layer was dried with anhydrous Na_2SO_4 and the solvent was evaporated to afford the crude product, which was purified by column chromatography on silica gel eluting with DCM/MeOH (100/1-80/1) to give the **DR-011-G0-MAL** (267.3 mg, yield 71.2%) as a yellow sticky oil. ^{1}H NMR (300 MHz, $CDCl_3$, ppm): δ 7.40 – 7.09 (m, 8H), 6.84 (s, 2H), 6.50 (s, 4H), 5.25 (s, 4H), 4.04 (s, 2H), 3.95 – 3.82 (m, 1H), 3.59 – 3.37 (m, 8H), 3.31 (dd, *J* = 18.7, 9.0 Hz, 1H), 3.08 – 2.58 (m, 11H), 1.82 – 1.20 (m, 20H). ^{13}C NMR (75 MHz, $CDCl_3$, ppm): δ 176.31 , 175.51 , 174.64 , 173.78 , 169.55 , 141.16 , 140.23 , 136.53 , 134.21 , 129.73 , 126.49 , 126.18 , 80.90 , 47.37 , 46.69 , 41.10 , 38.96 , 38.74 , 38.44 , 36.10 , 32.02 , 31.21 , 28.73 , 28.08 , 27.35 , 26.04.

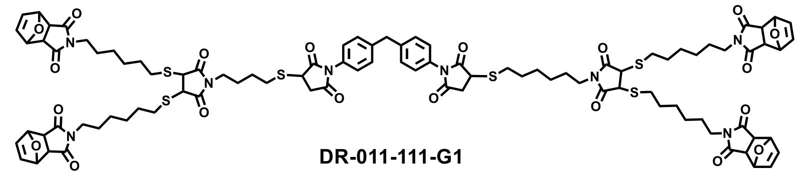

DR-011-111-G1: DR-011-G0-MAL (364.4 mg, 0.33 mmol) and **DN-111-G0-SH** (232.2 mg, 0.30 mmol) were dissolved in 20.0 mL dry $CHCl_3$ in a 50 mL three-neck round-bottom flask under argon atmosphere at 25 ºC. TEA (0.1 mL, 0.56 mmol) was add *via* a 1 mL syringe over 5 min. The mixture was stirred for about 12 h. ^{1}H NMR indicated the reaction was complete. The reaction mixture was washed with saturated $NaHCO_3$ (aq.) (15.0 mL) and water (15.0 mL). The combined organic layer was dried with anhydrous Na_2SO_4 and the solvent was evaporated to afford the crude product, which was purified by column chromatography on silica gel eluting with DCM/MeOH (100/1-80/1) to give the **DR-011-111-G1** (433.3 mg, yield 76.9%) as a yellow sticky oil. ^{1}H NMR (300 MHz, $CDCl_3$,

ppm): δ 7.26 (m, 8H), 6.50 (s, 8H), 5.25 (s, 8H), 4.04 (s, 2H), 3.93 – 3.79 (m, 2H), 3.64 – 3.39 (m, 16H), 3.31 (dd, J = 18.7, 9.1 Hz, 2H), 3.07 – 2.55 (m, 20H), 1.86 – 0.96 (m, 44H). ^{13}C NMR (75 MHz, $CDCl_3$, ppm): δ 176.29, 175.54, 174.64, 173.83, 136.54 , 129.78 , 126.49 , 80.91 , 47.39 , 46.68 , 38.77 , 36.15 , 32.00 , 29.31 , 28.76 , 28.12 , 27.37 , 26.08. MALDI-TOF for **DR-011-111-G1**, Calcd: *m/z* = 1627.52 [M + Na-4Fu]$^+$; Found: 1627.95 [M + Na-4Fu]$^+$.

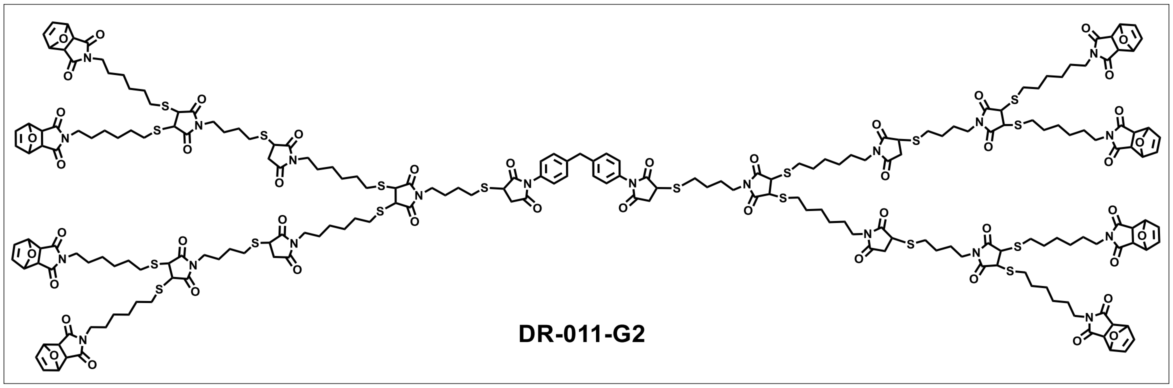

DR-011-G2: DN-011-G0-SH and **DR-011-G1-MA** precursors to **DR-011-G2** was prepared using the procedures described in the literature.[3]

DR-011-G1-MA (60.0 mg, 0.038 mmol) and **DN-011-G0-SH** (170.1 mg, 0.228 mmol) were dissolved in 10.0 mL dry $CHCl_3$ in a 50 mL three-neck round-bottom flask under argon atmosphere at 25 °C. TEA (0.2 mL, 1.12 mmol) was add *via* a 1 mL syringe over 5 min. The mixture was stirred for about 12 h. ^{1}H NMR indicated the reaction was complete. The reaction mixture was washed with saturated $NaHCO_3$ (aq.) (15.0 mL) and water (15.0 mL). The combined organic layer was dried with anhydrous Na_2SO_4 and the solvent was evaporated to afford the crude product, which was purified by column chromatography on silica gel eluting with DCM/MeOH (100/1-60/1) to give the **DR-011-G2** (99.0 mg, yield 57.1%) as a yellow sticky oil. ^{1}H NMR (300 MHz, $CDCl_3$, ppm) δ 7.29 (m, 8H), 6.51 (s, 16H), 5.25 (s, 16H), 4.04 (d, J = 2.8 Hz, 2H), 3.91 – 3.82 (m, 2H), 3.73 – 3.65 (m, 4H), 3.67 – 3.28 (m, 48H), 3.32 (dd, J = 19.0, 9.3 Hz, 2H), 3.12 (dd, J = 18.7, 9.0 Hz, 4H), 3.06 – 2.57 (m, 54H), 2.48 (dd, J = 18.6, 3.5 Hz, 4H), 1.95 – 0.99 (m, 120H). ^{13}C NMR (75 MHz, $CDCl_3$, ppm) δ 176.27 , 174.60 , 136.53 , 129.75 , 126.48 , 80.90 , 47.38 , 46.70 , 38.75 , 36.00 , 32.04 , 31.09 , 28.74 , 28.10 , 27.35 , 26.05. MALDI-TOF for **DR-011-G2**, Calcd: *m/z* = 4036.29 [M + Na-8Fu]$^+$; Found: 4036.58 [M + Na-8Fu]$^+$.

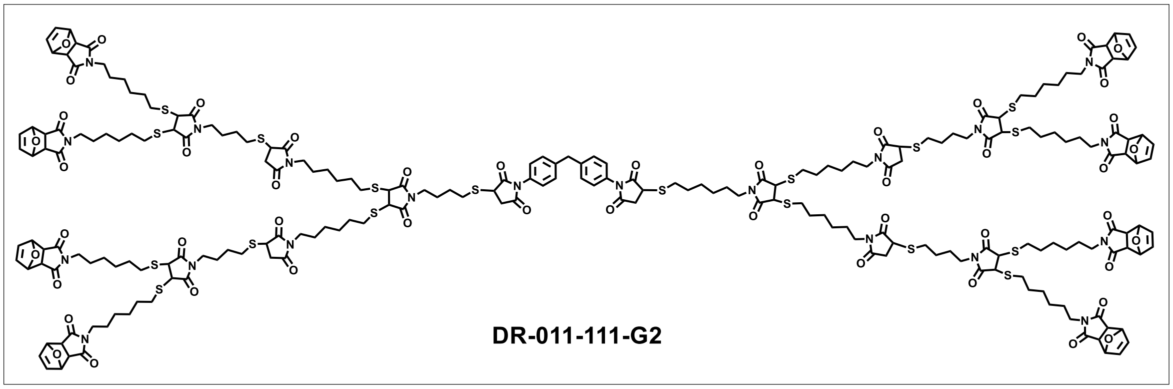

DR-011-111-G2: DN-011-G0-SH and **DR-011-111-G1-MA** precursors to **DR-011-111-G2** was prepared using the procedures described in the literature.[3]

DR-110-111-G1-MA (170.8 mg, 0.11 mmol) and **US-011-G0-SH** (492.3 mg, 0.66 mmol) were dissolved in 15.0 mL dry $CHCl_3$ in a 50 mL three-neck round-bottom flask under argon atmosphere at 25 ^{o}C. TEA (0.6 mL, 3.36 mmol) was add *via* a 1 mL syringe over 5 min. The mixture was stirred for about 12 h. ^{1}H NMR indicated the reaction was complete. The reaction mixture was washed with saturated $NaHCO_3$ (aq.) (15.0 mL) and water (15.0 mL). The combined organic layer was dried with anhydrous Na_2SO_4 and the solvent was evaporated to afford the crude product, which was purified by column chromatography on silica gel eluting with DCM/MeOH (100/1-60/1) to give the **DR-011-111-G2** (282.2 mg, yield 55.9%) as a yellow sticky oil. ^{1}H NMR (300 MHz, $CDCl_3$, ppm) δ 7.36 – 7.12 (m, 8H), 6.51 (s, 16H), 5.26 (s, 16H), 4.04 (d, *J* = 3.9 Hz, 2H), 3.87 (dd, *J* = 9.0, 3.3 Hz, 2H), 3.70 (dd, *J* = 8.9, 3.5 Hz, 4H), 3.64 – 3.21 (m, 48H), 3.31 (dd, *J* = 18.7, 9.2 Hz, 2H), 3.11 (dd, *J* = 18.7, 9.0 Hz, 4H), 3.02 – 2.60 (m, 54H), 2.48 (dd, *J* = 18.7, 3.6 Hz, 4H), 1.91 – 1.13 (m, 124H). ^{13}C NMR (75 MHz, $CDCl_3$, ppm) δ 176.28 , 174.62 , 136.54 , 129.77, 126.48 , 80.91 , 47.39 , 46.71 , 38.77 ,35.99, 32.06 , 31.12, 28.75 , 28.12 , 27.37 , 26.08. MALDI-TOF for **DR-011-111-G2**, Calcd: *m/z* = 4064.32 $[M + Na\text{-}8Fu]^{+}$; Found: 4064.64 $[M + Na\text{-}8Fu]^{+}$

Measurements and Analysis

SEC: The number-average molecular weight (M_n) and polydispersity ($Đ = M_w/M_n$) of the polymers were determined using a size exclusion column TOSOH HLC-8320 equipped with refractive index and UV detectors using two TSKgel Super Mutipore HZ-N (4.6 × 150 mm, 3 μm beads size) columns arranged in series, and it can separate polymers in the molecular weight range 500-1.9 × 10^5 g/mol. THF was used as the eluent at a flow rate of 0.35 mL/min at 40 °C. Data acquisition

was performed using EcoSEC software, and molecular weights were calculated with polystyrene (PS) standards.

NMR: All ^{1}H NMR, ^{13}C NMR spectra were collected using a Bruker nuclear magnetic resonance instrument (300 MHz) using tetramethylsilane (TMS) as the internal standard at room temperature. The ^{1}H NMR spectra were referenced to 7.26 ppm in $CDCl_3$, and ^{13}C NMR spectra were referenced to 77.00 ppm in $CDCl_3$.

DSC. Differential scanning calorimetry (DSC) was performed with heating and cooling at a rate of 10 °C/min with a limited temperature from -80 °C – 100 °C on a Q200 differential scanning calorimeter (TA Instruments), and the glass transition temperature (T_g) was measured on the third cycle of a heat/cool/heat experiment.

TGA. Thermal characterization of all the generations of unimolecule macromolecules was carried out using thermogravimetric analysis (TGA) on a PerkinElmer Pyris 1 instruments. Samples were run in a platinum TGA pans at a ramp rate of 10 ^{o}C per minute from 30 to 800 ^{o}C under the nitrogen atmosphere. (Note: In order to completely remove the protecting group of furan, we hold the temperature at 100 ^{o}C for 1 min).

Matrix barcode reader application on the smart phone was designed and programmed by Beijing Power-software Co., Ltd.

Computer Simulation of Bond Dissociation Energy (BDE)

Model molecule / BDE (kcal/mol)	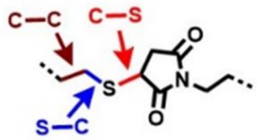 STE	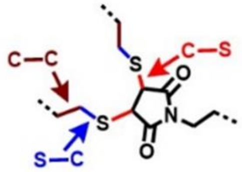 STE-1
C-S	57.16	56.80
S-C	69.24	68.70
C-C	~79.9	

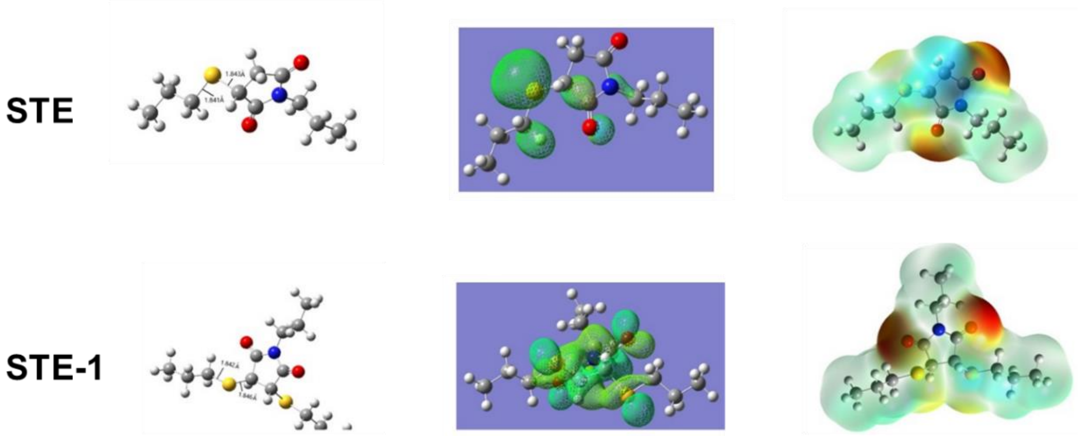

Supplementary Figure 8. Computer simulations of bond dissociation energy (BDE) of chemical bonds of succinimide thioether and its derivate. The molecular geometry optimized using the Density Functional Theory method of the three parameter Becke-style hybrid functional (B3LYP) with the basis set of 6-311+g (d. p). All calculations were performed using GAUSSIAN 2009 package.

Thermal Characterization

a) *Differential Scanning Calorimetry*

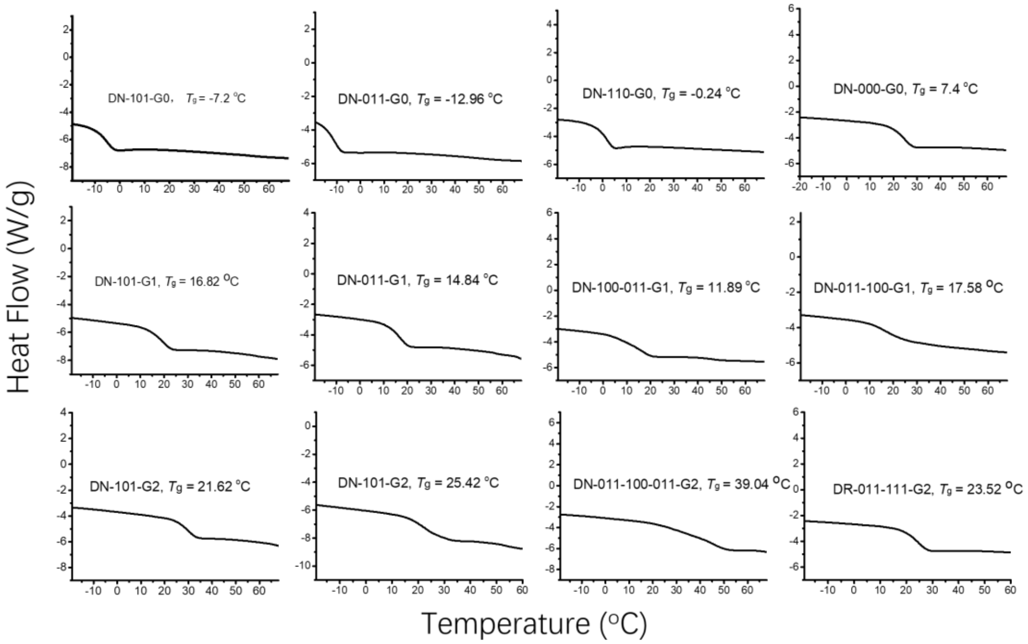

Supplementary Figure 9. DSC traces of monomer (G0), 1st generation (G1) and 2nd generation (G2) of the dendrimers. (-80 °C – 100 °C, and the glass transition temperature T_g was measured on the third cycle of a heat/cool/heat experiment).

b) *Thermal Gravimetric Analysis*

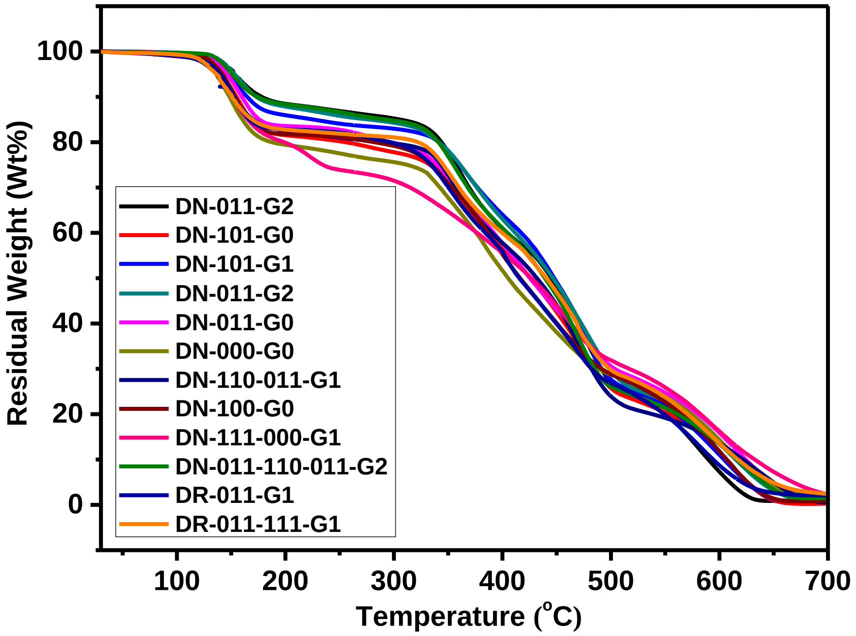

Supplementary Figure 10. TGA traces for monomers (G0), 1st generation (G1) and 2nd generation (G2) of the dendrimers.

Algorithm of Binary Tree based Calculation

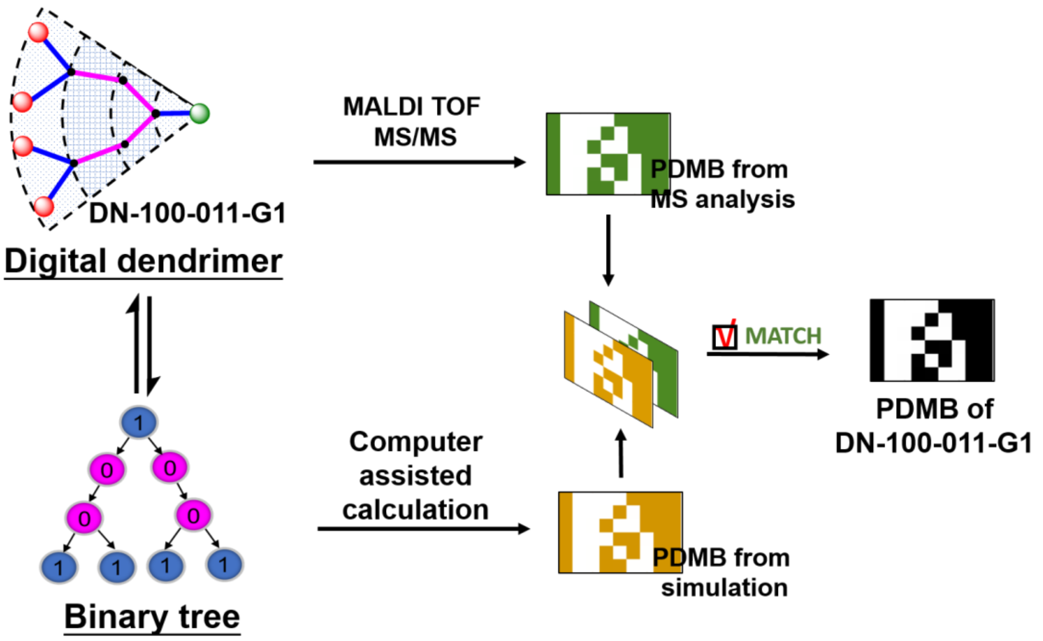

Algorithm 1: ExtractPath

Input: gene structure $\boldsymbol{G}$
Global Variable: paths ← []
Build tree based on the molecule structure: T ← **BuildTree**(G)
Traverse the tree and collect all the possible paths: **TraverseTree**($T, path$ = "")
Sort and visualize all the paths

ExtractPath is the main function. Given the structure description of a structure of dendron or dendrimer, this function first constructs the corresponding tree structure, and then calls **TraverseTree** to extract all the possible paths. The extracted paths are then sorted and visualized.

Algorithm 2: TraverseTree

Input: tree structure T, partially generated path: $path$
Get leaf nodes: $leaves$ ← T.GetLeaves()
for each leaf node v **in** leaves:
    Append v to $path$
    if v is the ROOT of T:
        Add $path$ to $\boldsymbol{paths}$ and **return**
    else:
        Make a copy of T: T' ← $copy(T)$
        Update T' by deleting v: T' ← $DeleteNode(T', v)$
        TraverseTree($T', path$)

TraverseTree is a recursively executed function which traverses the tree structure from leaves to the root node and extract all the possible paths.

Supplementary Figure 11. Schematic representation of computer assisted construction of data matrix barcode and algorithm of binary-tree based calculation (marked with grey).

Applications Based on Readable Dendrimer Data Matrix Barcodes

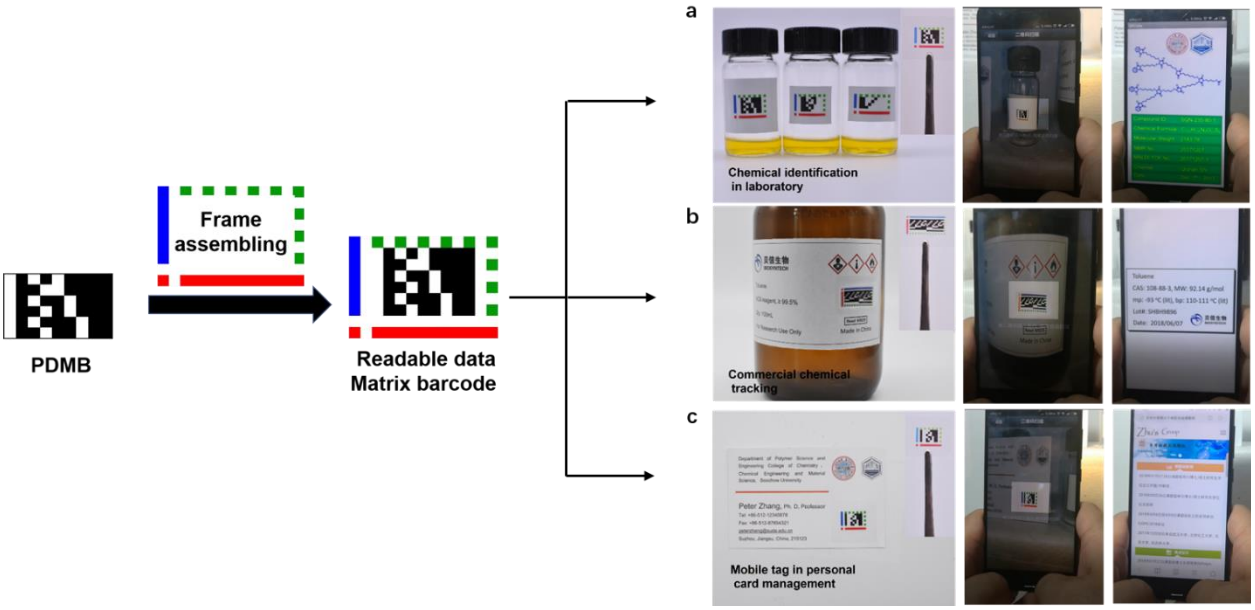

Supplementary Figure 12. Equipping PDMB with finder patterns (blue and pink) and a dashed pattern (green) to a readable data matrix barcode, coded specific information in the data matrix barcode can be extracted with a smartphone via a specific application. Therefore, they could be used for (a) chemical identification in laboratory, (b) commercial chemical tracking and (c) mobile tag in personal card management (also watch the video in supporting information)

Dendrimer Extraction from the Anti-Counterfeit Tag

To an oven dried 50 mL beaker, small pieces of cut tag and ~10 mL anhydride THF was added. Stirred for ~1.5h, before the small pieces were almost completely dissolved in THF. Then the THF solvent was removed to obtain a colorless gel-like solid. 10mL of the mix solvent (chloroform: petrol ether (v/v) = 1:1) was added to the residue and stirred for 5 min to extract the dendrimer and precipitate the PVC. The mixture was filtered and filtrate was concentrated to obtain the dendrimer as a pale-yellow oil which was used directly in MALDI-TOF and MALDI-TOF MS/MS analysis without any further purification.

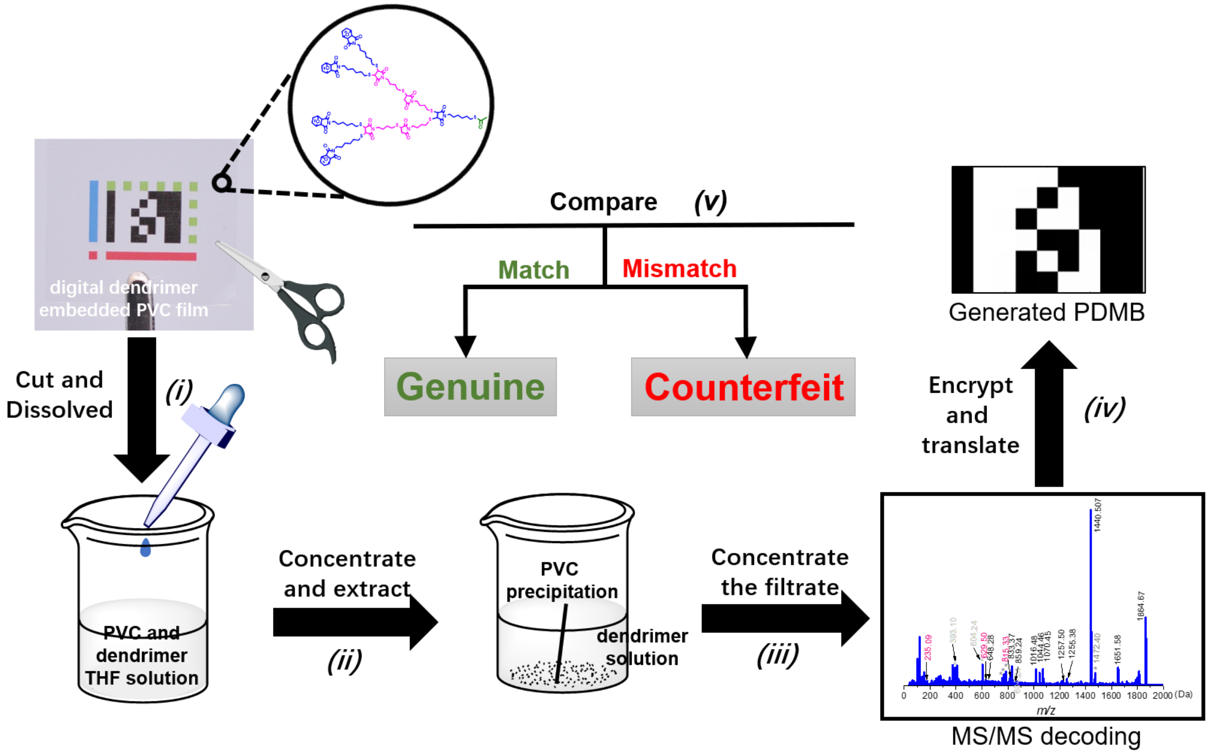

Supplementary Figure 13. Anticounterfeiting labeling by using the digital dendrimer **DN-100-011-G1**: (i) cut and dissolved in THF, (ii) concentrated and extracted by chloroform: petrol ether = 1:1, (iii) concentrated the filtrate to get the dendrimer (MS sample), (iv) data encrypted and translated to generate PDMB, (v) compare the PDMB with the printed data matrix barcode on PVC film.

Supplementary Figures

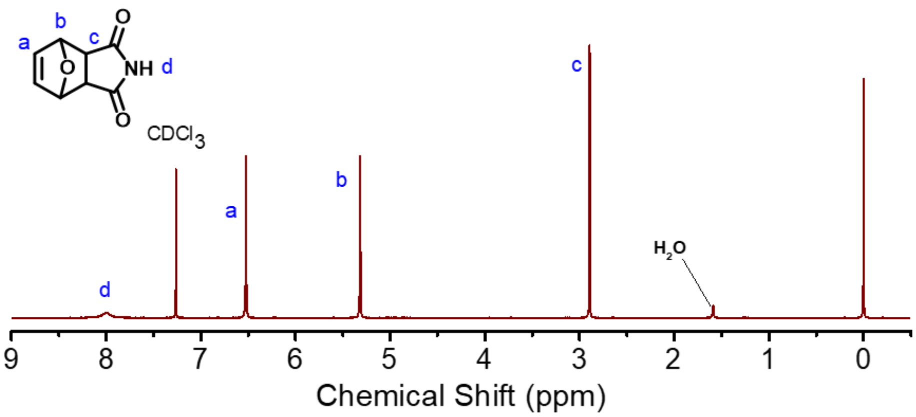

Supplementary Figure 14. ^{1}H NMR spectrum of compound **FU-MA** in $CDCl_3$ (Bruker, 300 MHz, TMS)

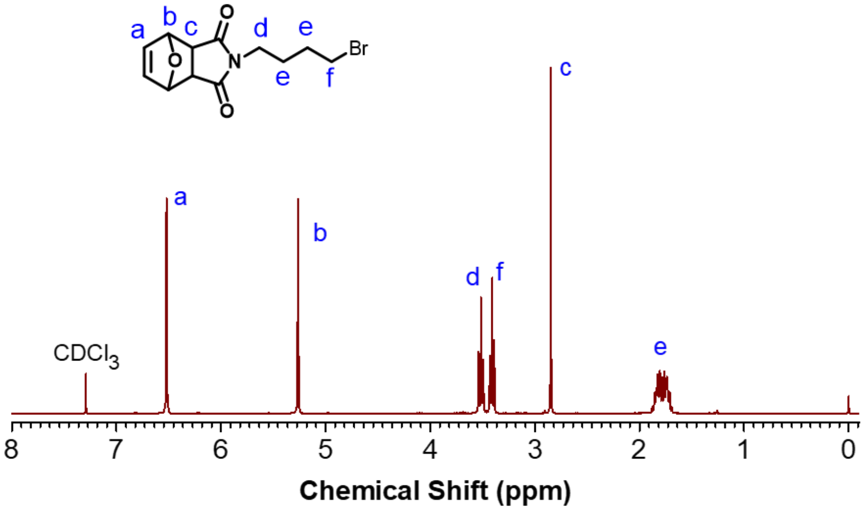

Supplementary Figure 15. ^{1}H NMR spectrum of compound **FU-MA –Br (n=4)** in $CDCl_3$ (Bruker, 300MHz, TMS).

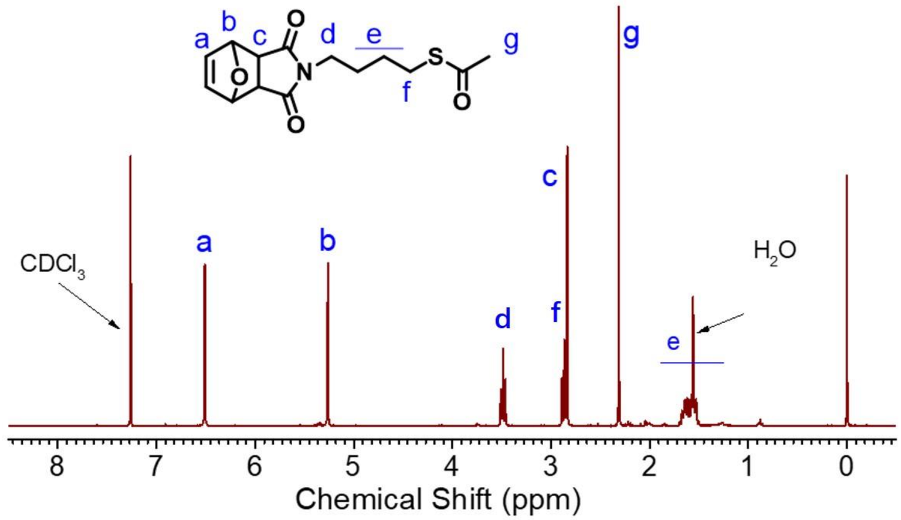

Supplementary Figure 16. ^{1}H NMR spectrum of compound **sub-monomer (n = 4)** in $CDCl_3$ (Bruker, 300 MHz, TMS)

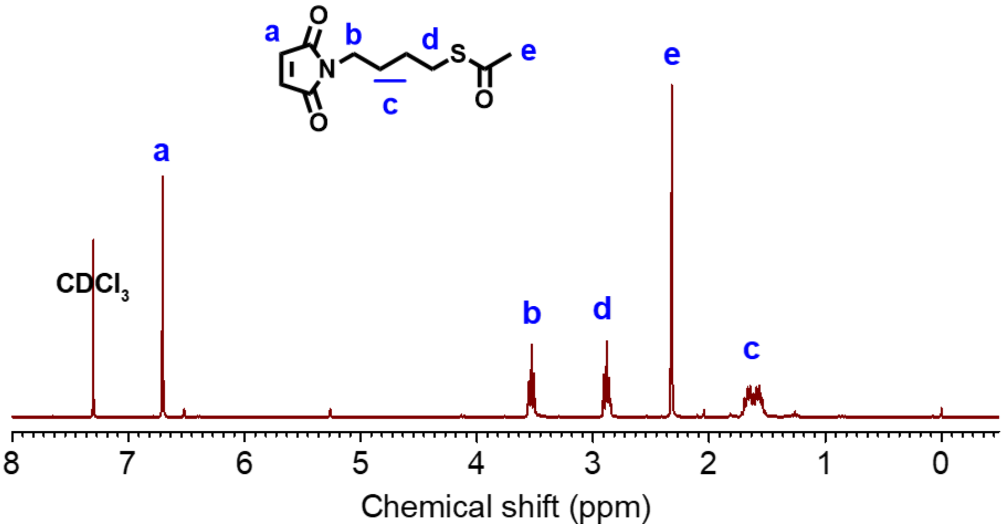

Supplementary Figure 17. ^{1}H NMR spectrum of compound **C4-MA** in $CDCl_3$ (Bruker, 300 MHz, TMS)

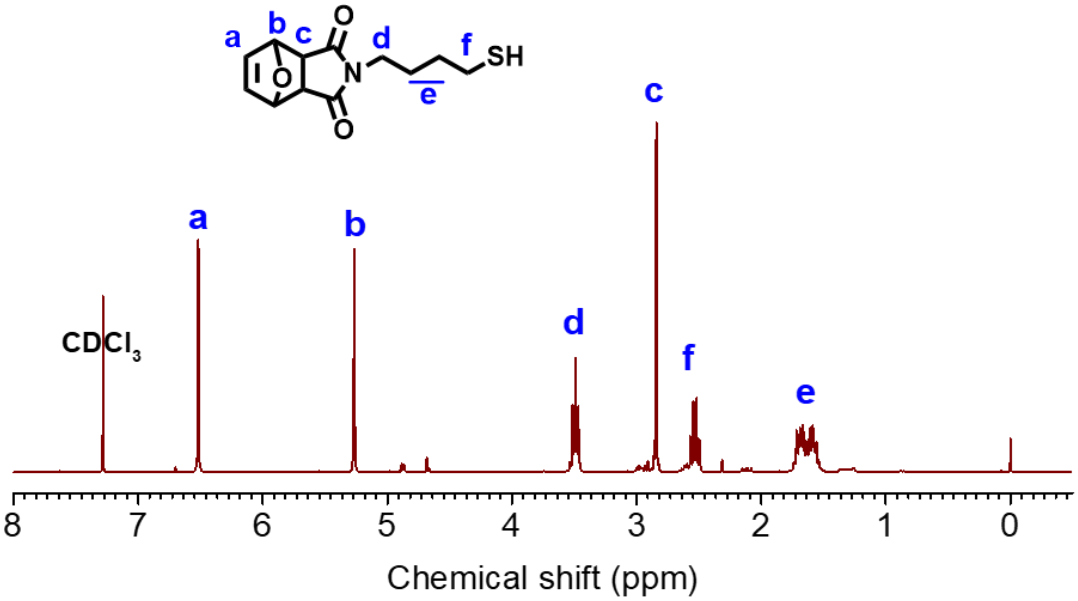

Supplementary Figure 18. ^{1}H NMR spectrum of compound **C4-SH** in $CDCl_3$ (Bruker, 300 MHz, TMS)

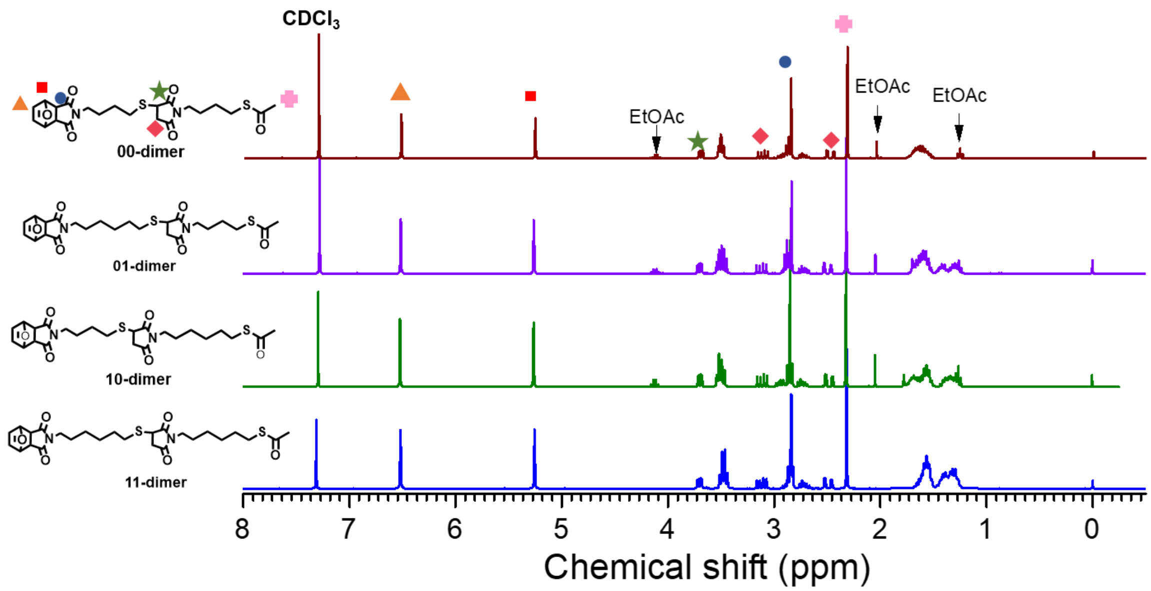

Supplementary Figure 19. ^{1}H NMR spectra of compound **dimers** in $CDCl_3$ (Bruker, 300 MHz, TMS)

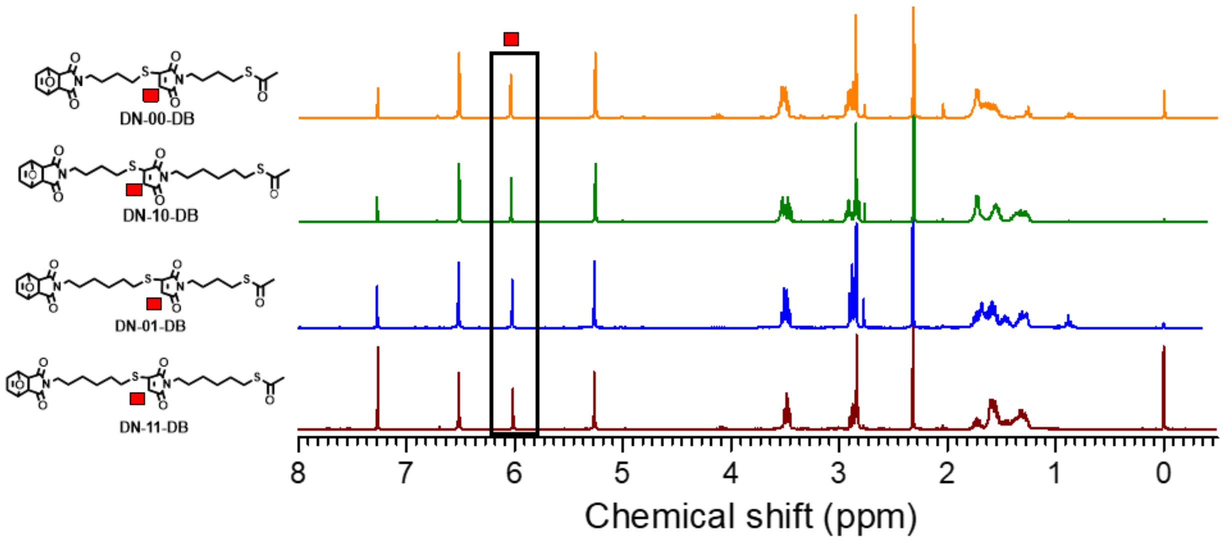

Supplementary Figure 20. ^{1}H NMR spectra of compound **DN-00-DB, DN-10-DB, DN-01-DB and DN-11-DB** in $CDCl_3$ (Bruker, 300 MHz, TMS)

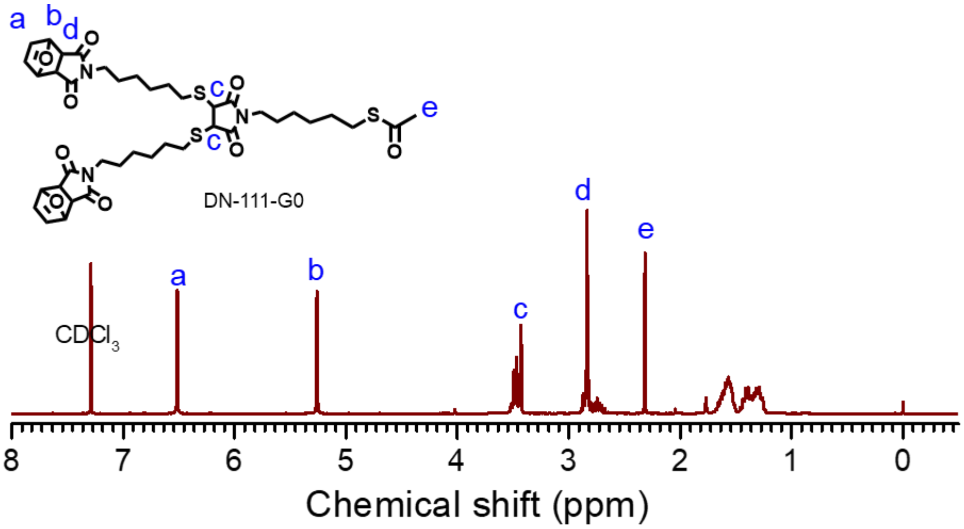

Supplementary Figure 21. ^{1}H NMR spectrum of compound **DN-111-G0** in $CDCl_3$ (Bruker, 300 MHz, TMS)

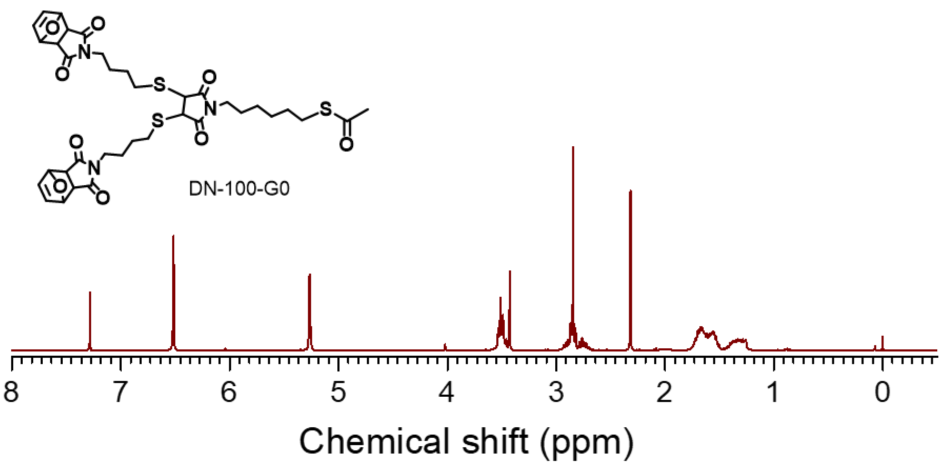

Supplementary Figure 22. ^{1}H NMR spectrum of compound **DN-100-G0** in $CDCl_3$ (Bruker, 300 MHz, TMS)

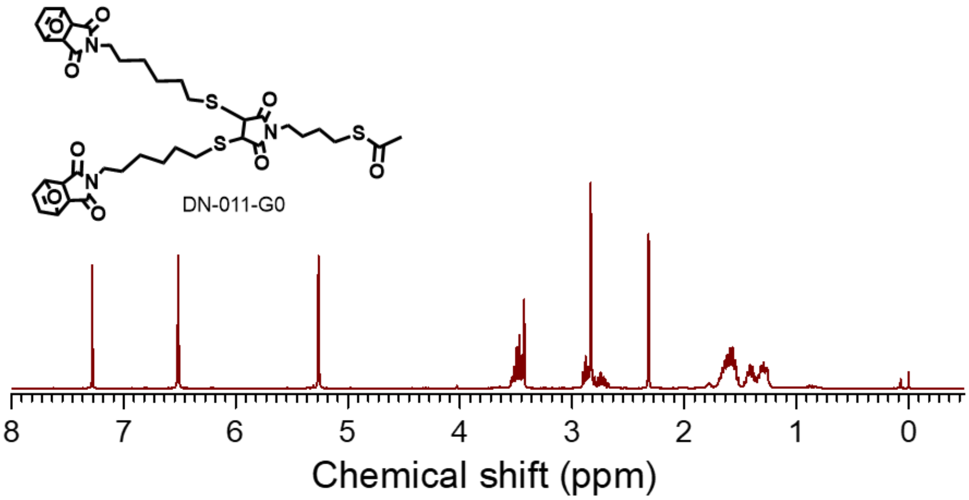

Supplementary Figure 23. ^{1}H NMR spectrum of compound **DN-011-G0** in $CDCl_3$ (Bruker, 300 MHz, TMS)

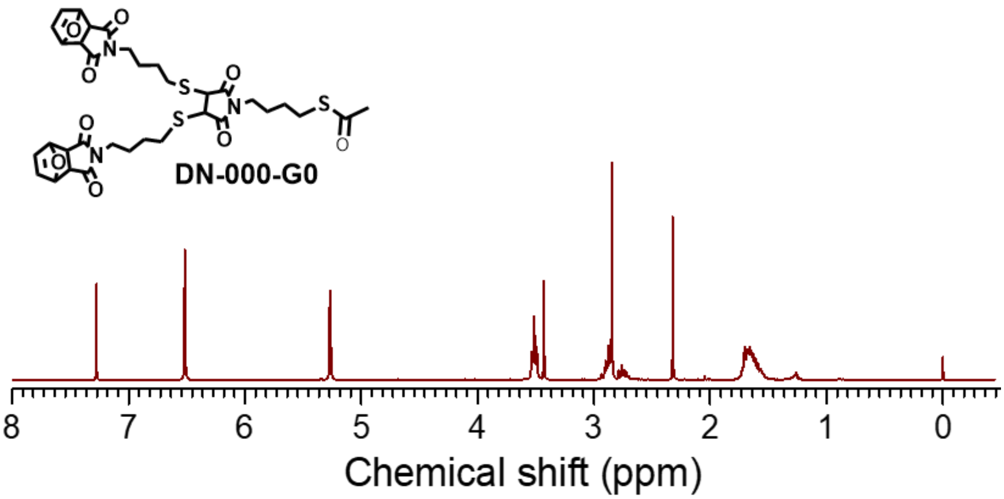

Supplementary Figure 24. ^{1}H NMR spectrum of compound **DN-000-G0** in $CDCl_3$ (Bruker, 300 MHz, TMS)

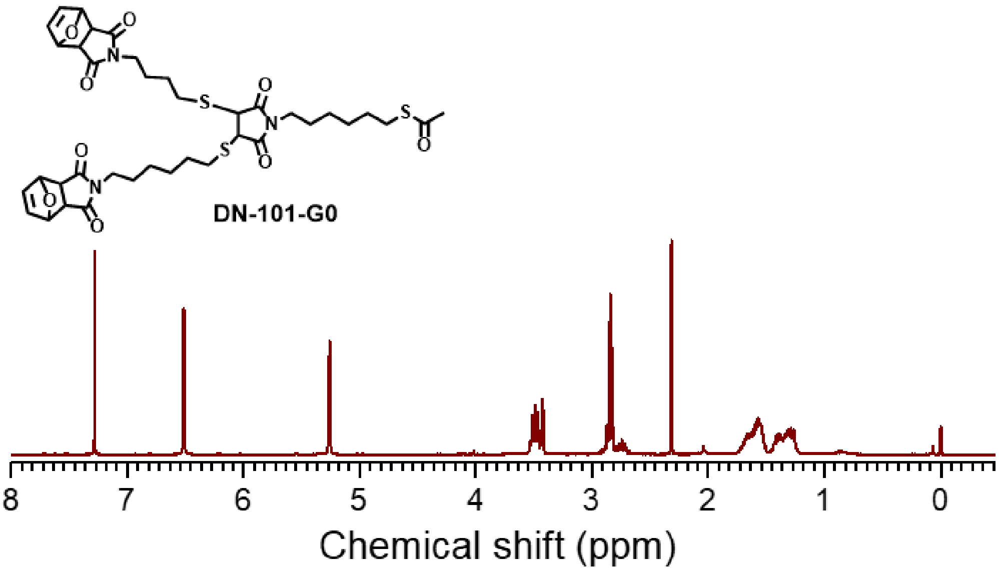

Supplementary Figure 25. ^{1}H NMR spectrum of compound **DN-101-G0** in $CDCl_3$ (Bruker, 300 MHz, TMS)

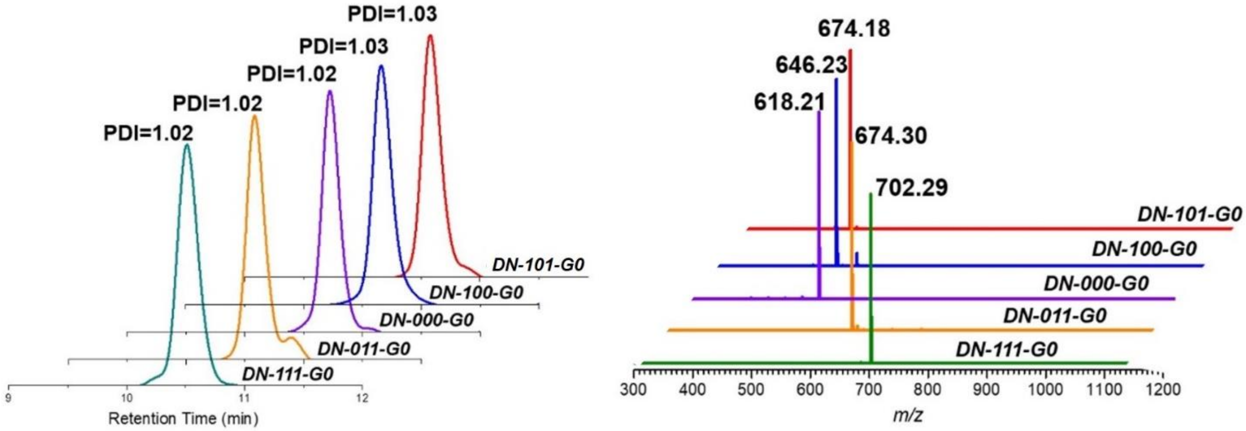

Supplementary Figure 26. SEC and MALDI-TOF spectra of the binary coded monomers

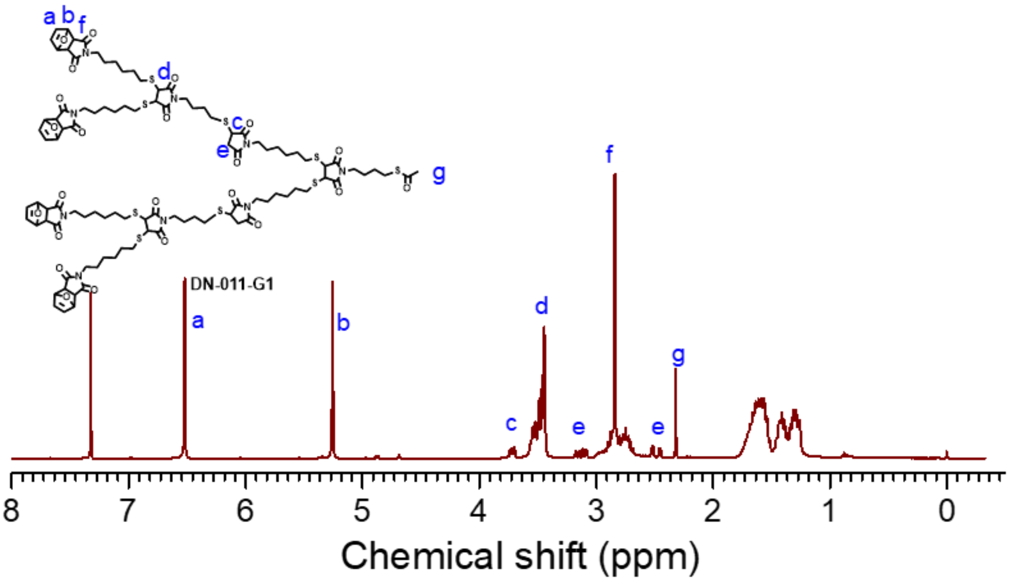

Supplementary Figure 27. ^{1}H NMR spectrum of compound **DN-011-G1** in $CDCl_3$ (Bruker, 300 MHz, TMS)

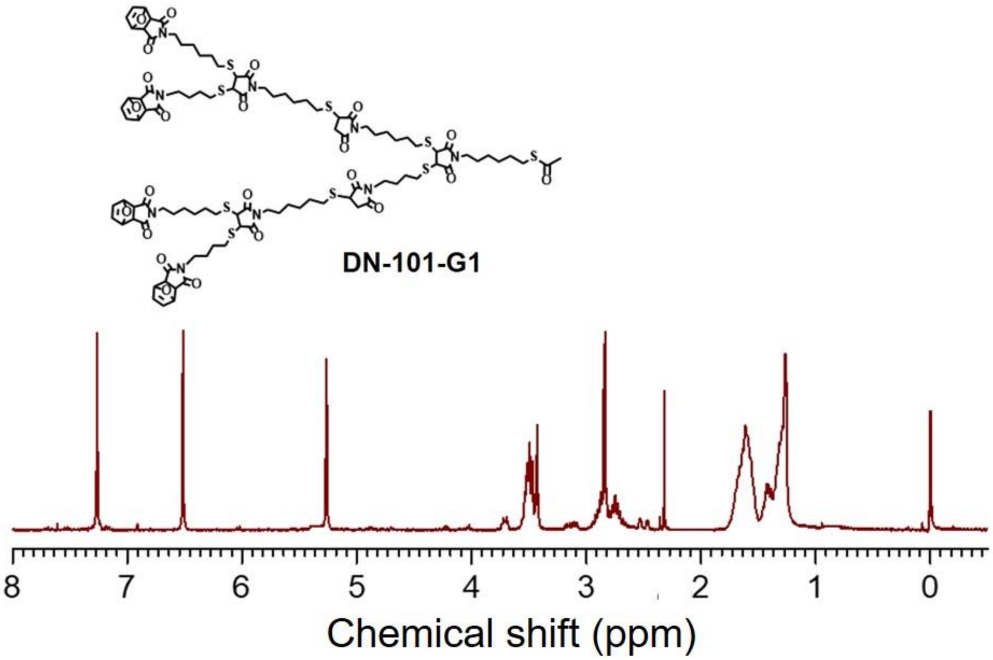

Supplementary Figure 28. ^{1}H NMR spectrum of compound **DN-101-G1** in $CDCl_3$ (Bruker, 300 MHz, TMS)

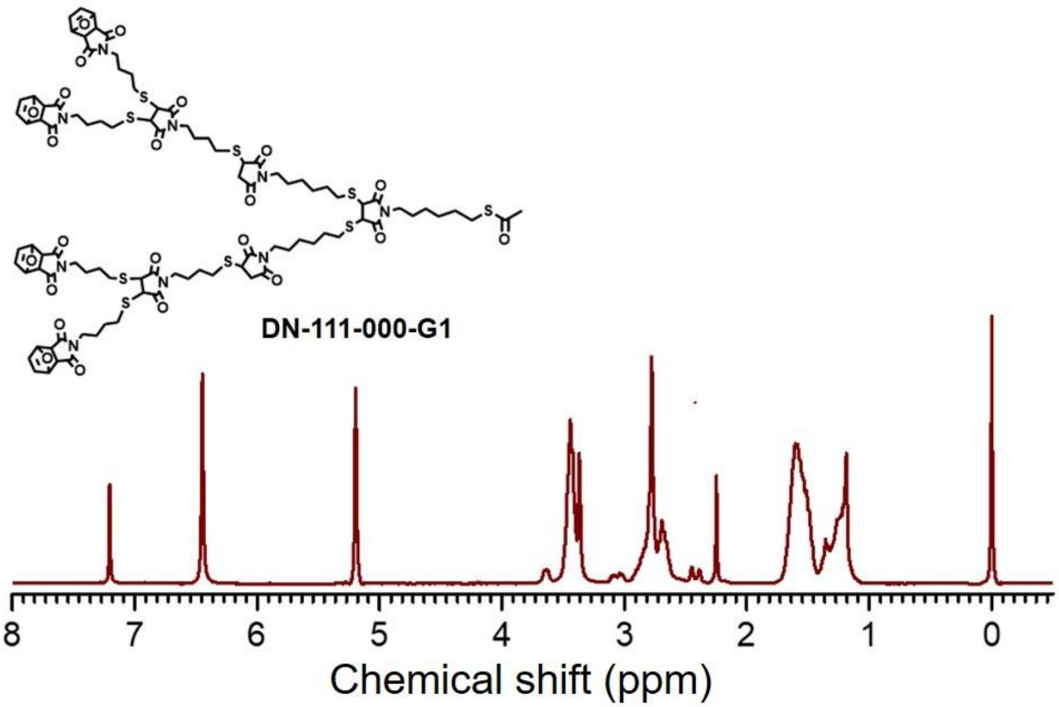

Supplementary Figure 29. ^{1}H NMR spectrum of compound **DN-111-000-G1** in $CDCl_3$ (Bruker, 300 MHz, TMS)

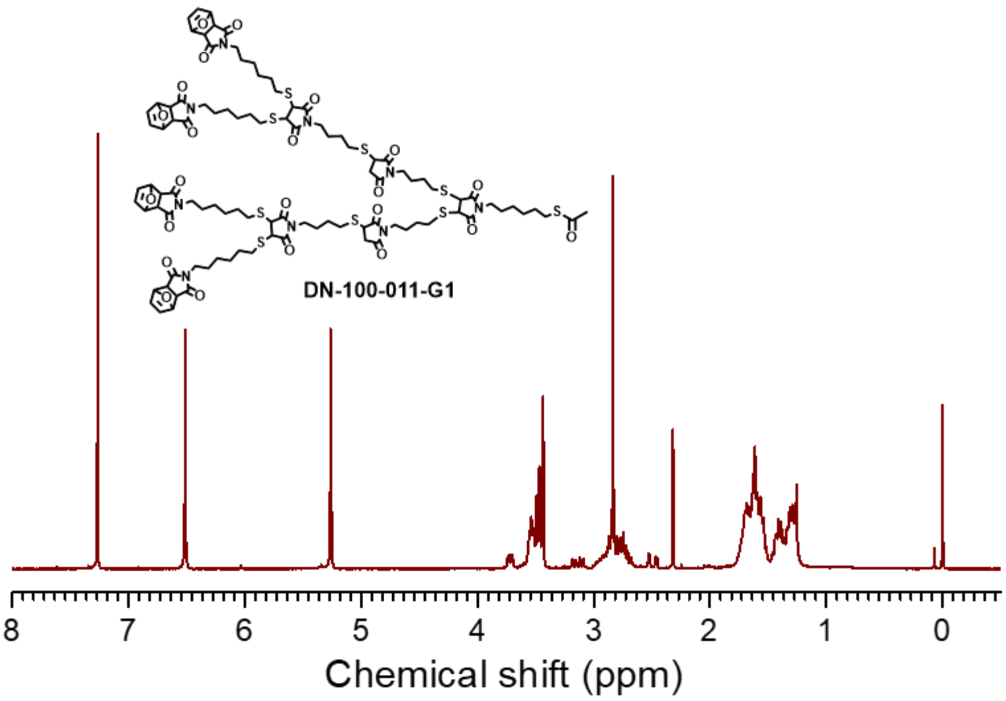

Supplementary Figure 30. ^{1}H NMR spectrum of compound **DN-100-011-G1** in $CDCl_3$ (Bruker, 300 MHz, TMS)

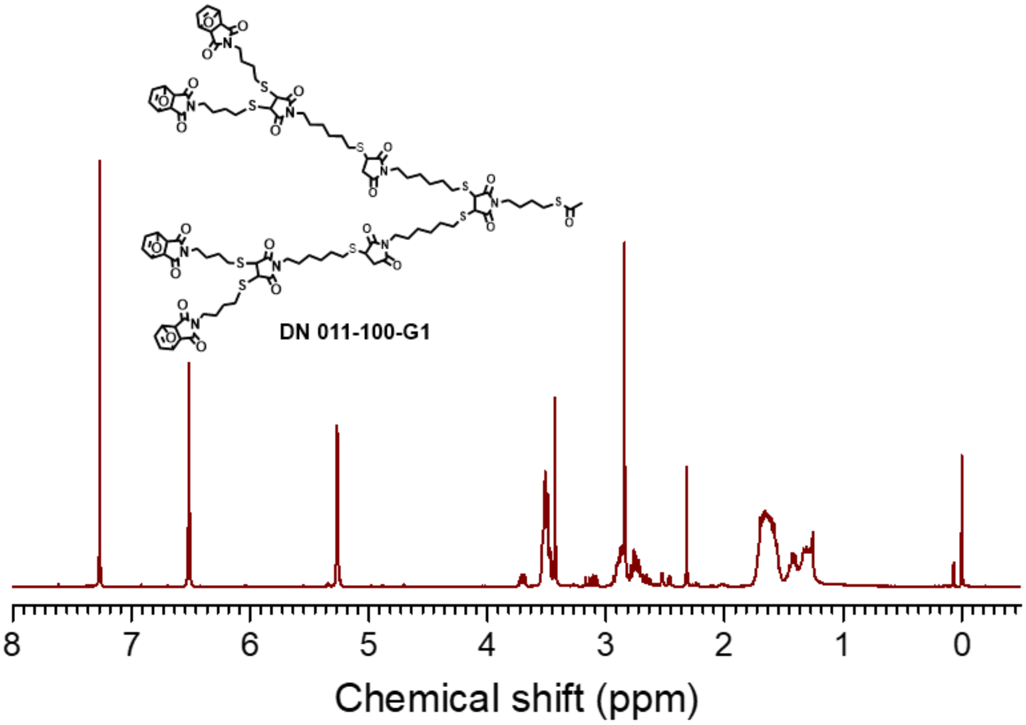

Supplementary Figure 31. ^{1}H NMR spectrum of compound **DN-011-100-G1** in $CDCl_3$ (Bruker, 300 MHz, TMS)

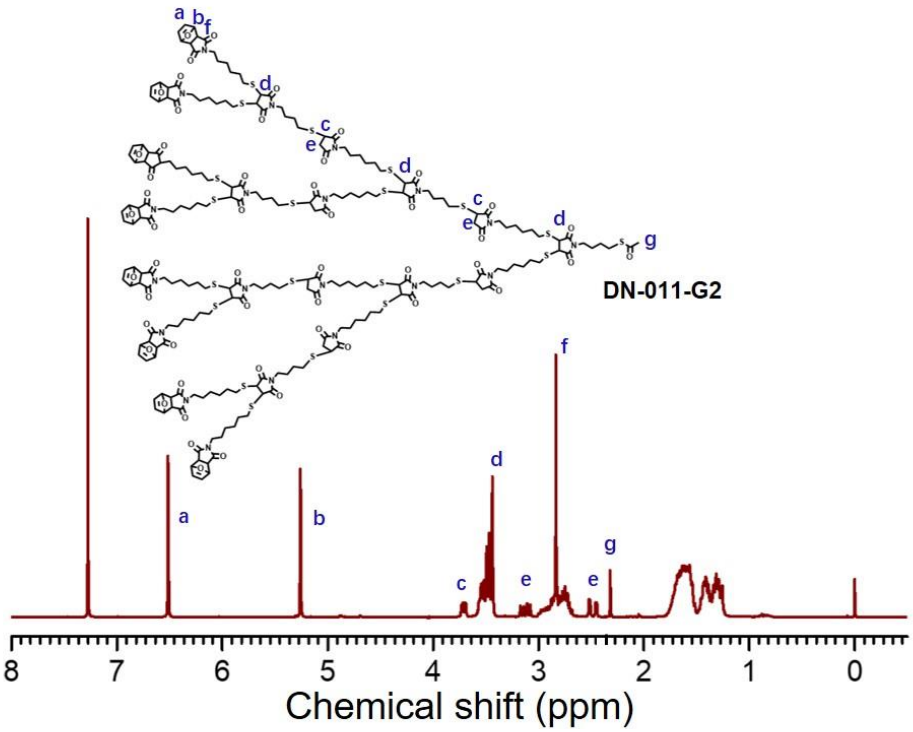

Supplementary Figure 32. ^{1}H NMR spectrum of compound **DN-011-G2** in $CDCl_3$ (Bruker, 300 MHz, TMS)

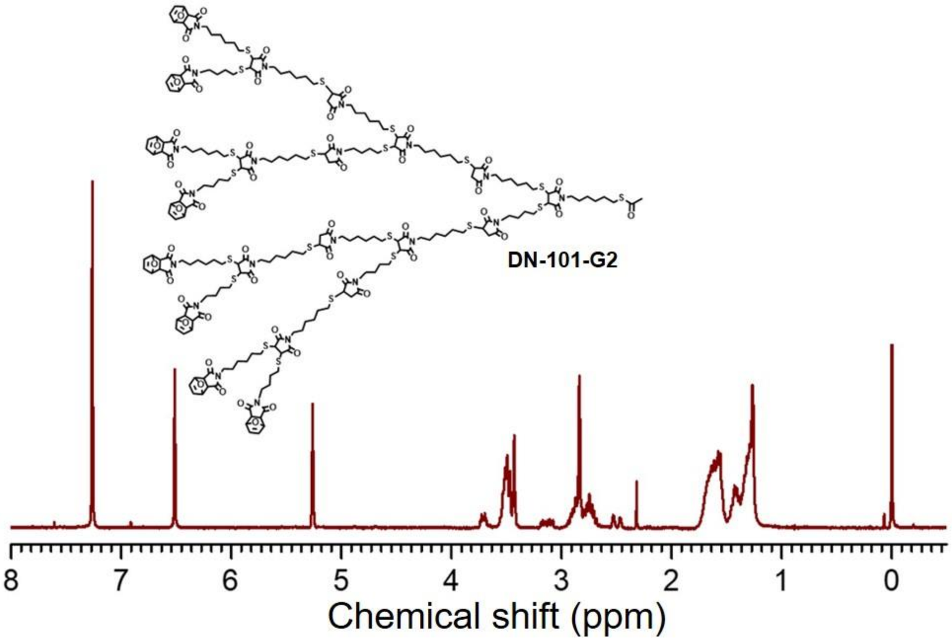

Supplementary Figure 33. ^{1}H NMR spectrum of compound **DN-101-G2** in $CDCl_3$ (Bruker, 300 MHz, TMS)

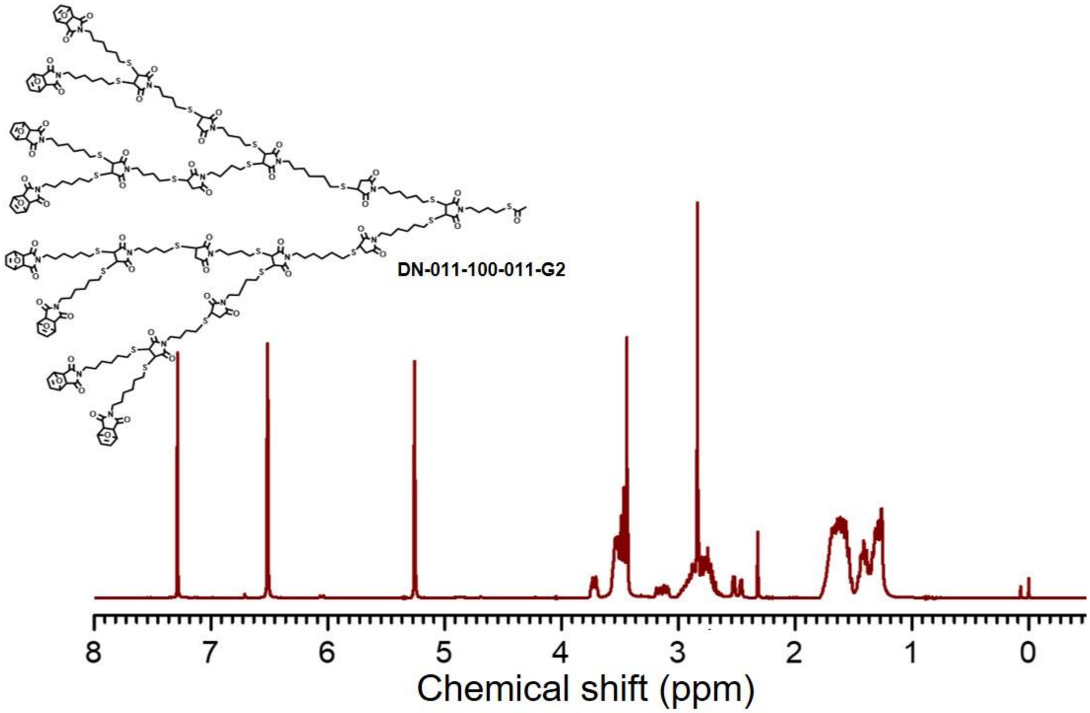

Supplementary Figure 34. ^{1}H NMR spectrum of compound **DN-011-100-011-G2** in $CDCl_3$ (Bruker, 300 MHz, TMS)

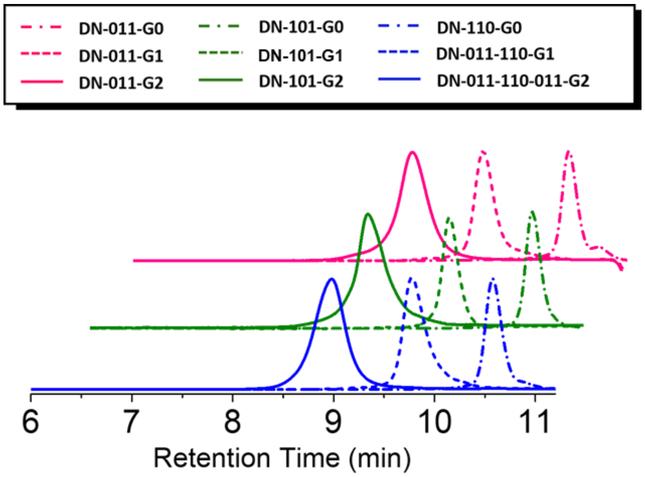

Supplementary Figure 35. SEC spectra of digital dendrimers (DN-011-G0 to G2, DN-101-G0 to G2 and alternating layers from G0-G2)

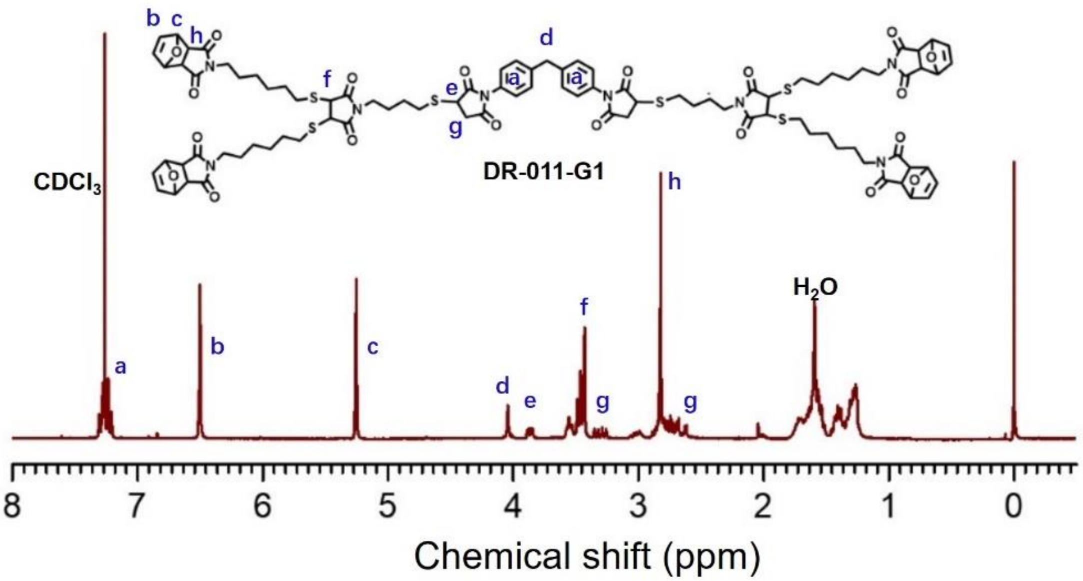

Supplementary Figure 36. ^{1}H NMR spectrum of compound **DR-011-G1** in $CDCl_3$ (Bruker, 300 MHz, TMS)

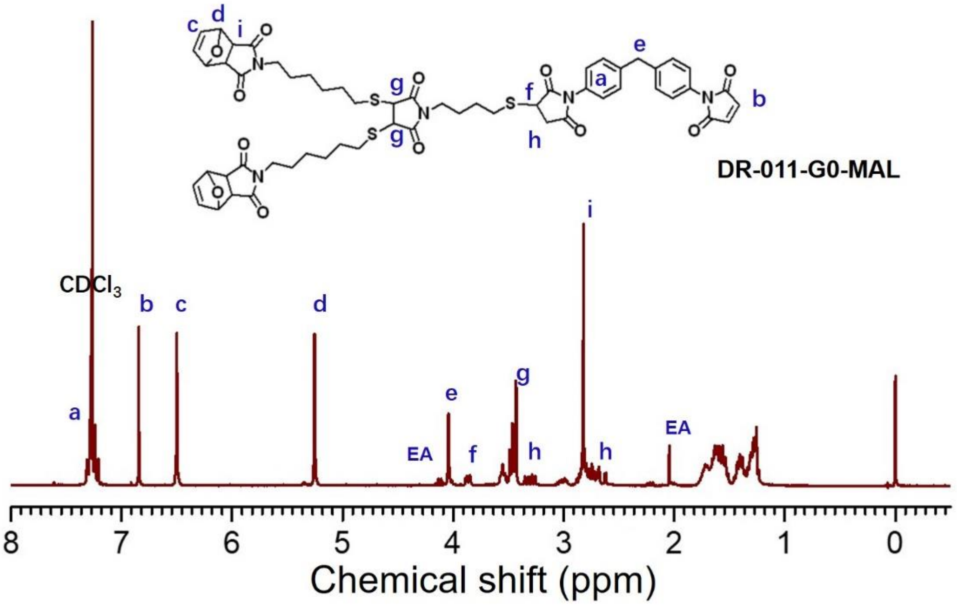

Supplementary Figure 37. ^{1}H NMR spectrum of compound **DR-011-G0-MAL** in $CDCl_3$ (Bruker, 300 MHz, TMS)

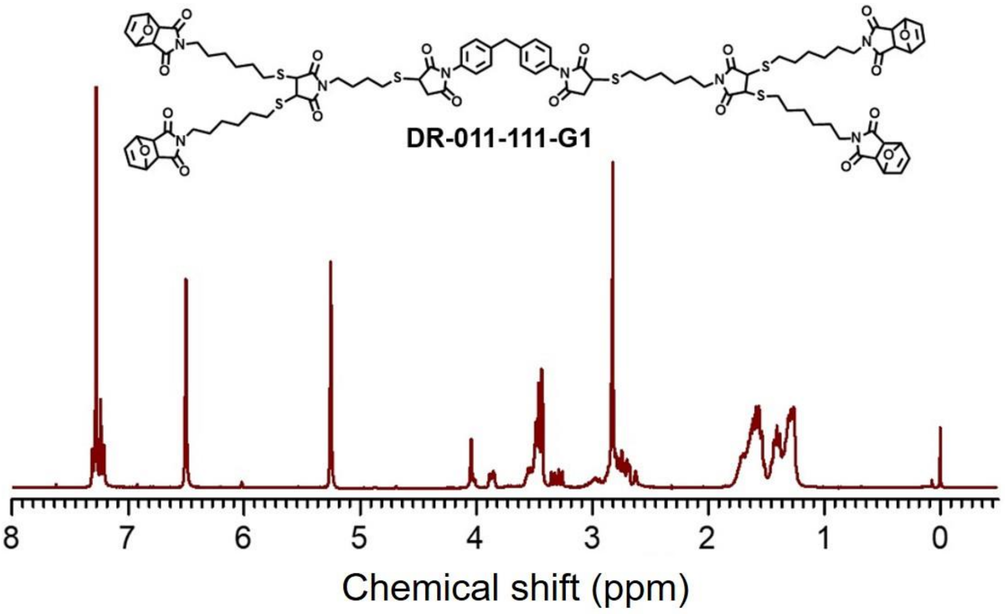

Supplementary Figure 38. ^{1}H NMR spectrum of compound **DR-011-111-G1** in $CDCl_3$ (Bruker, 300 MHz, TMS)

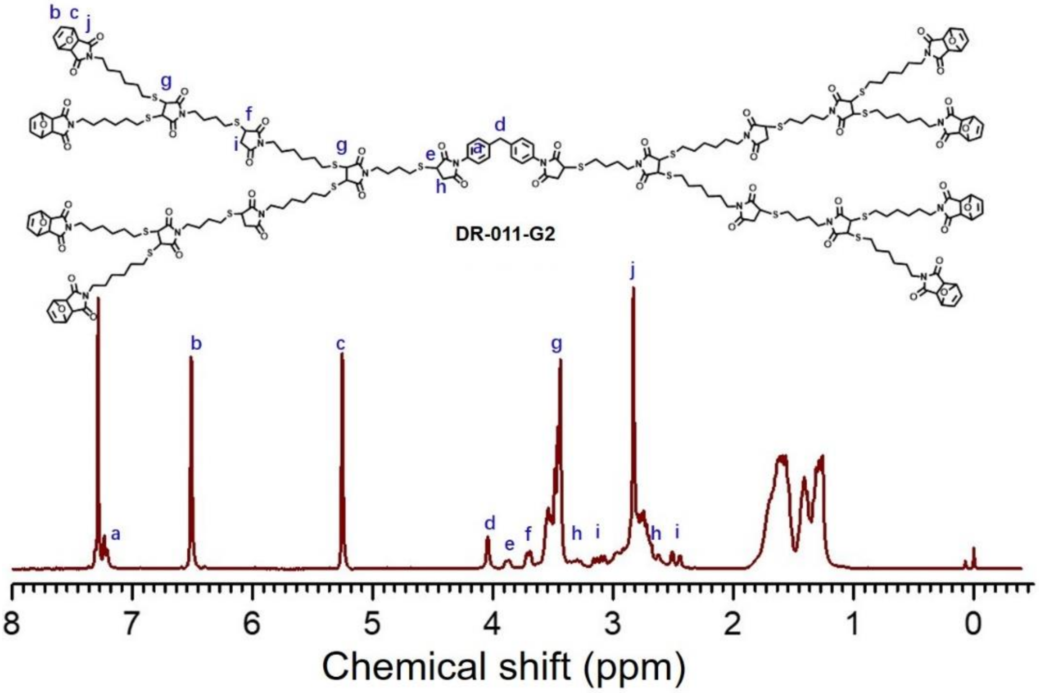

Supplementary Figure 39. ^{1}H NMR spectrum of compound **DR-011-G2** in $CDCl_3$ (Bruker, 300 MHz, TMS)

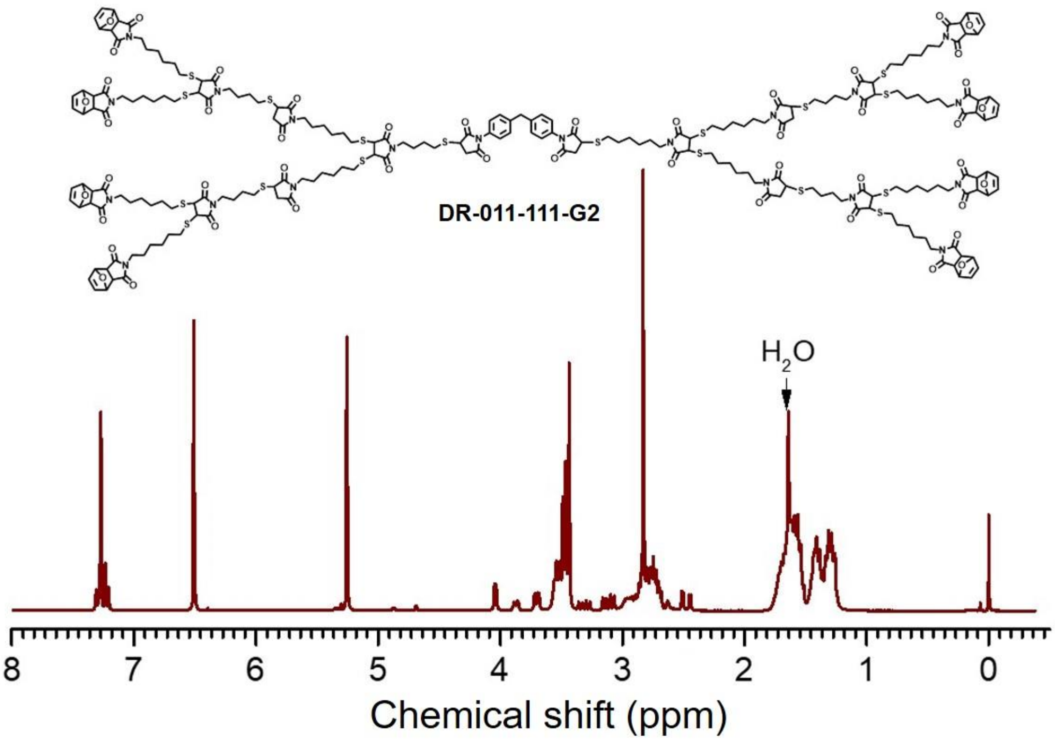

Supplementary Figure 40. ^{1}H NMR spectrum of compound **DR-011-111-G2** in $CDCl_3$ (Bruker, 300 MHz, TMS)

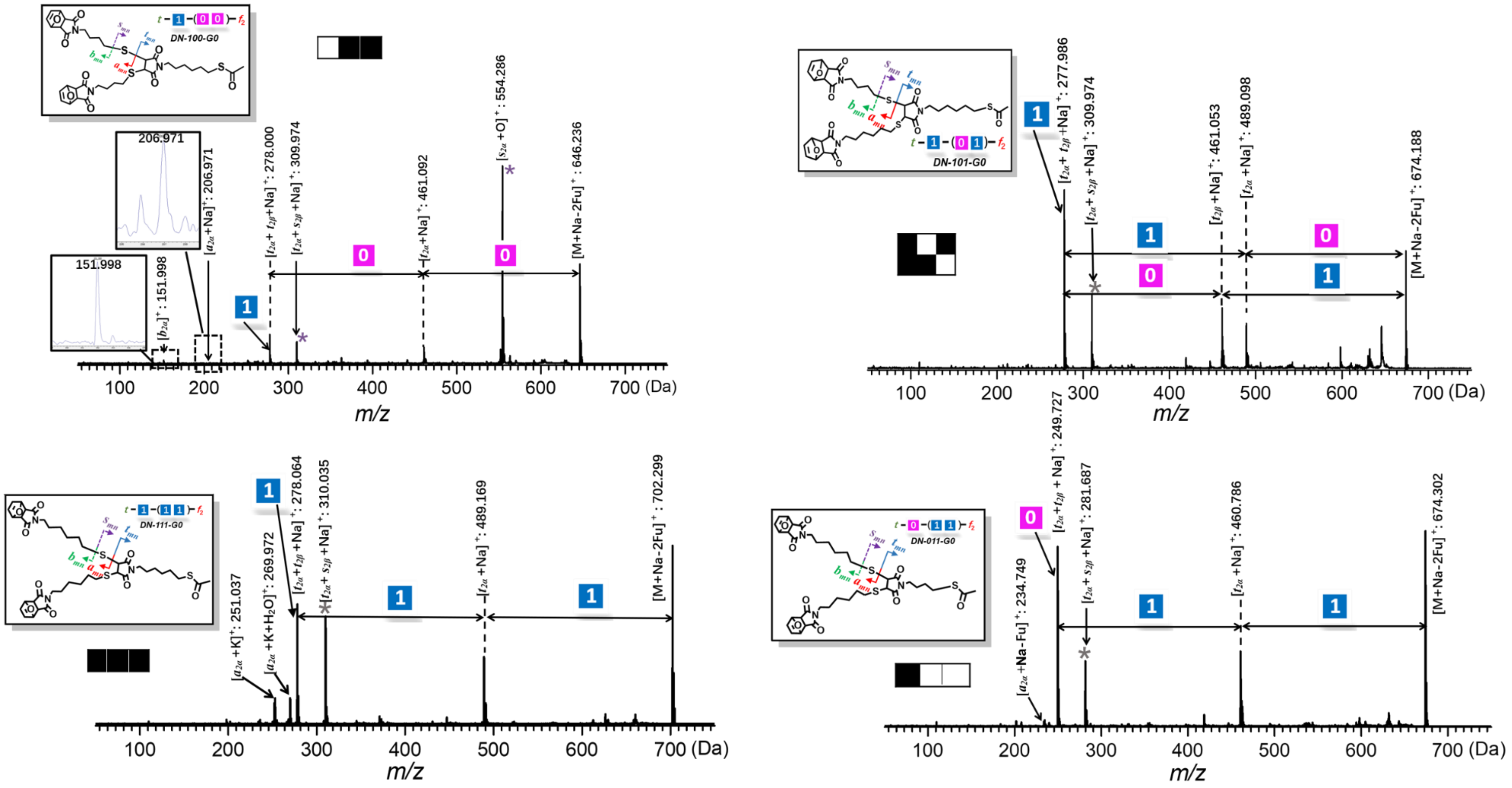

Supplementary Figure 41. MALDI TOF MS/MS of the binary coded monomers (**DN-100-G0**, **DN-101-G0**, **DN-1110-G0**, **DN-011-G0**). The MALDI TOF MS spectra of the monomers can be found in **Supplementary Figure 26**. * Internal fragments formed during secondary dissociation reactions.

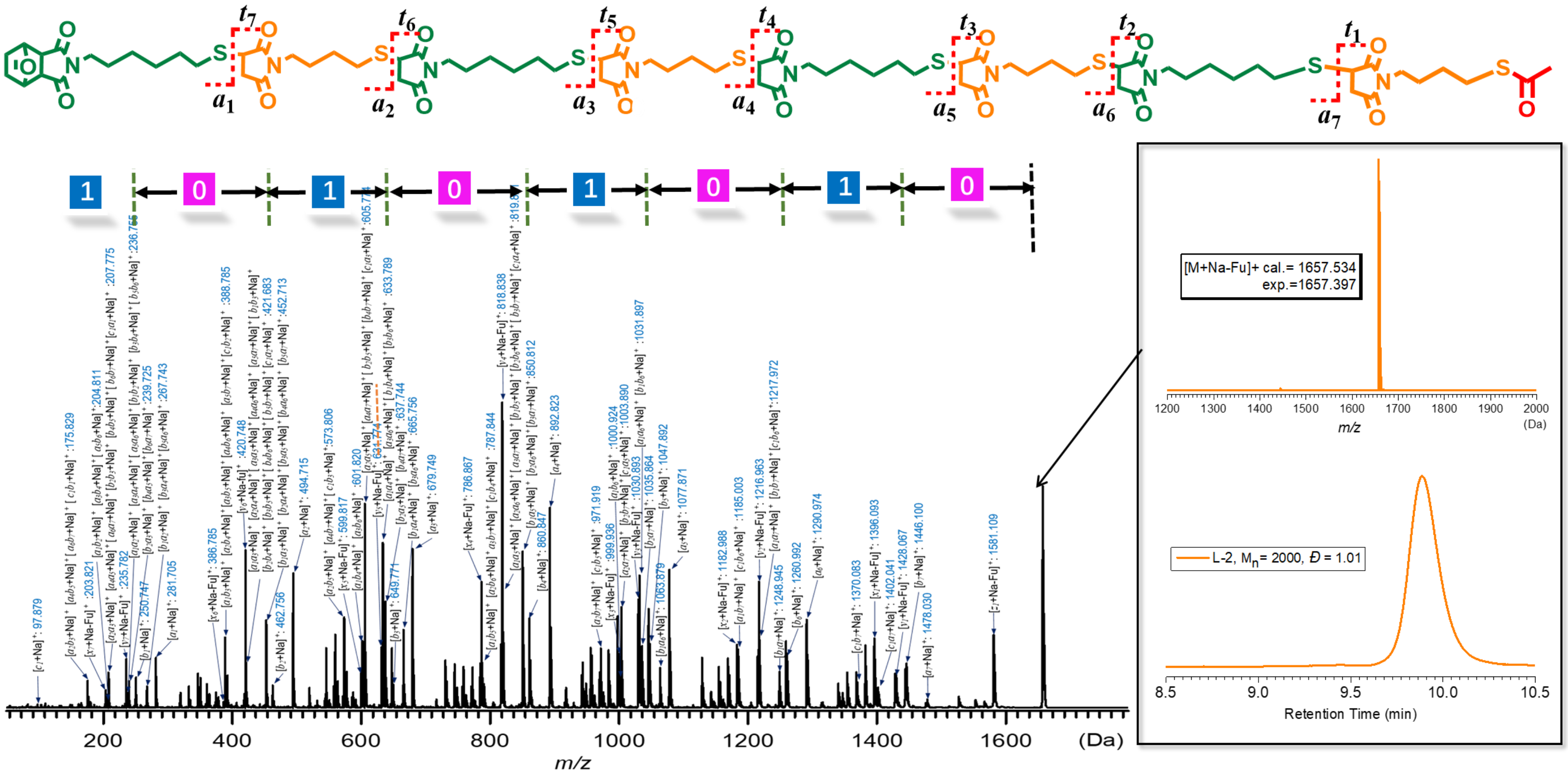

Supplementary Figure 42. Mass spectrometry analysis of the binary coded linear digital polymer **L-2**. MALDI TOF mass spectrum (right), showing the high monodispersity of the targeted species. MALDI TOF MS/MS mass spectrum (left), showing the assignment of dissociation fragments.

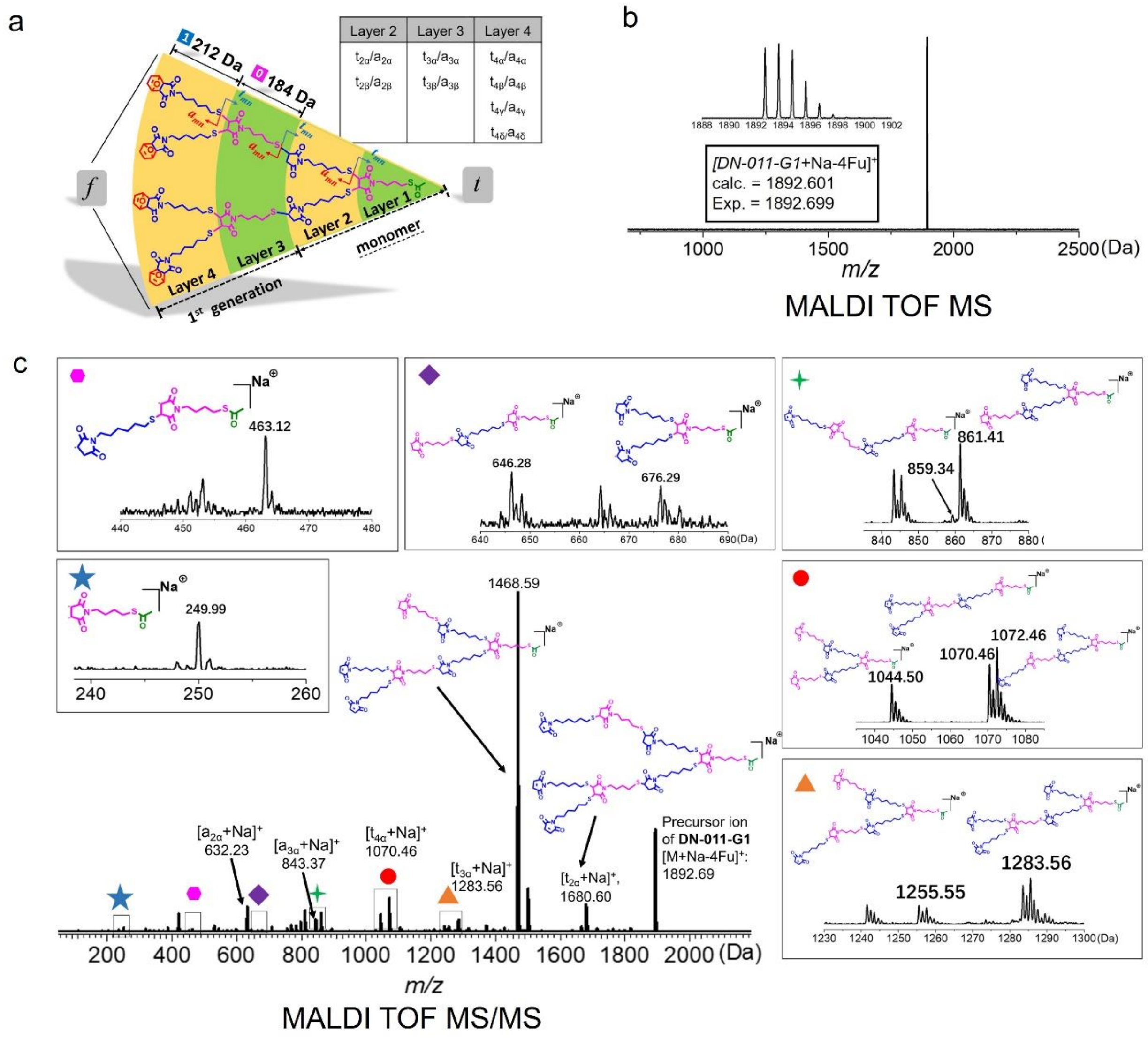

Supplementary Figure 43. a) Fragments with f end-group were named as a_{LB}, the complementary fragments with ***t*** terminals were named as t_{LB}**.** b) MALDI TOF MS and c) MALDI TOF MS/MS spectra of the binary coded monomers **DN-011-G1**. All the main fragments are carefully assigned.

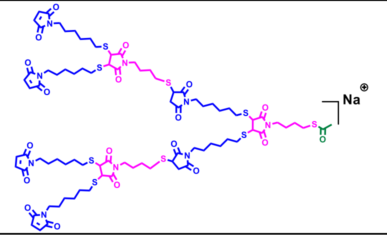	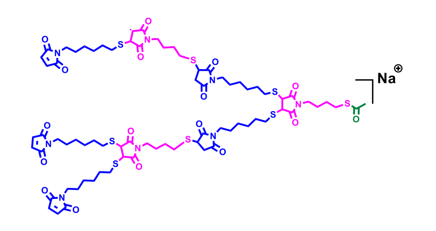	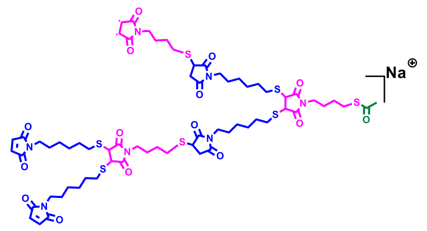	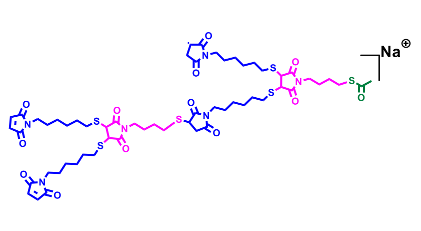
[M+Na-4Fu]$^{+}$<br>Cal.: 1892.60<br>Exp.: 1892.69	Fragment ion #1<br>Cal.: 1680.53<br>Exp.: 1680.60	Fragment ion #2<br>Cal.: 1468.45<br>Exp.: 1468.59	Fragment ion #3<br>Cal.: 1284.41<br>Exp.: 1283.56
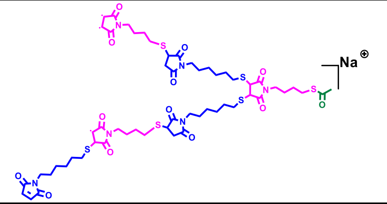	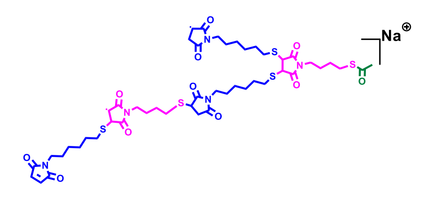	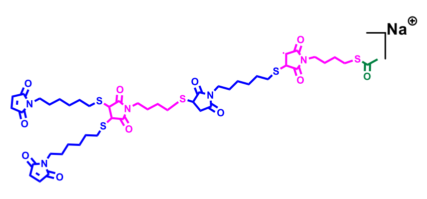	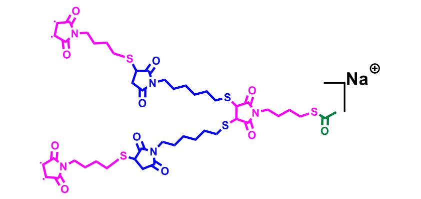
Fragment ion #4<br>Cal.: 1256.38<br>Exp.: 1255.55	Fragment ion #5<br>Cal.: 1072.33<br>Exp.: 1072.46	Fragment ion #6<br>Cal.: 1071.33<br>Exp.: 1070.46	Fragment ion #7<br>Cal.: 1044.30<br>Exp.: 1044.50
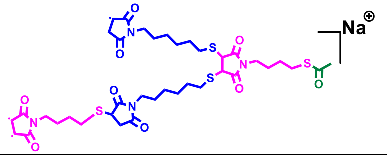	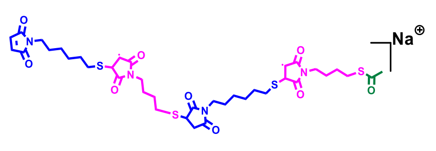	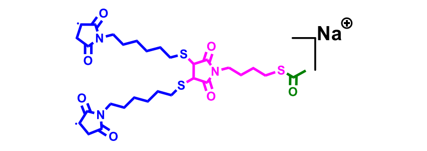	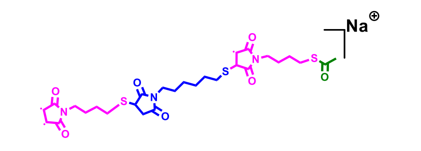
Fragment ion #8<br>Cal.: 860.26<br>Exp.: 861.41	Fragment ion #9<br>Cal.: 859.25<br>Exp.: 859.34	Fragment ion #10<br>Cal.: 676.22<br>Exp.: 676.29	Fragment ion #11<br>Cal.: 647.18<br>Exp.: 646.28
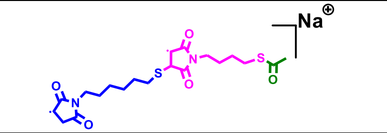	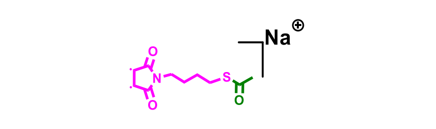		
Fragment ion #12<br>Cal.: 463.13<br>Exp.: 463.12	Fragment ion #13<br>Cal.: 250.05<br>Exp.: 249.99		

Supplementary Table 1. All the main fragment ions of the digital dendrimer **DN-011-G1**

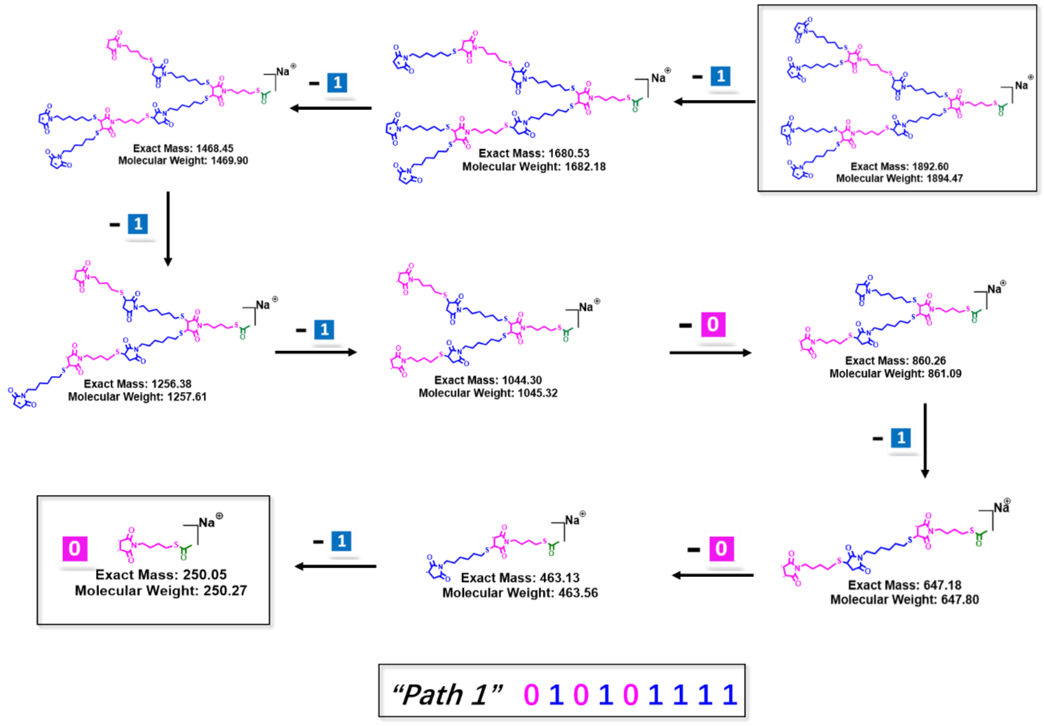

Supplementary Figure 44. Fragmentation pathway 1 of dendrimer **DN-011-G1**

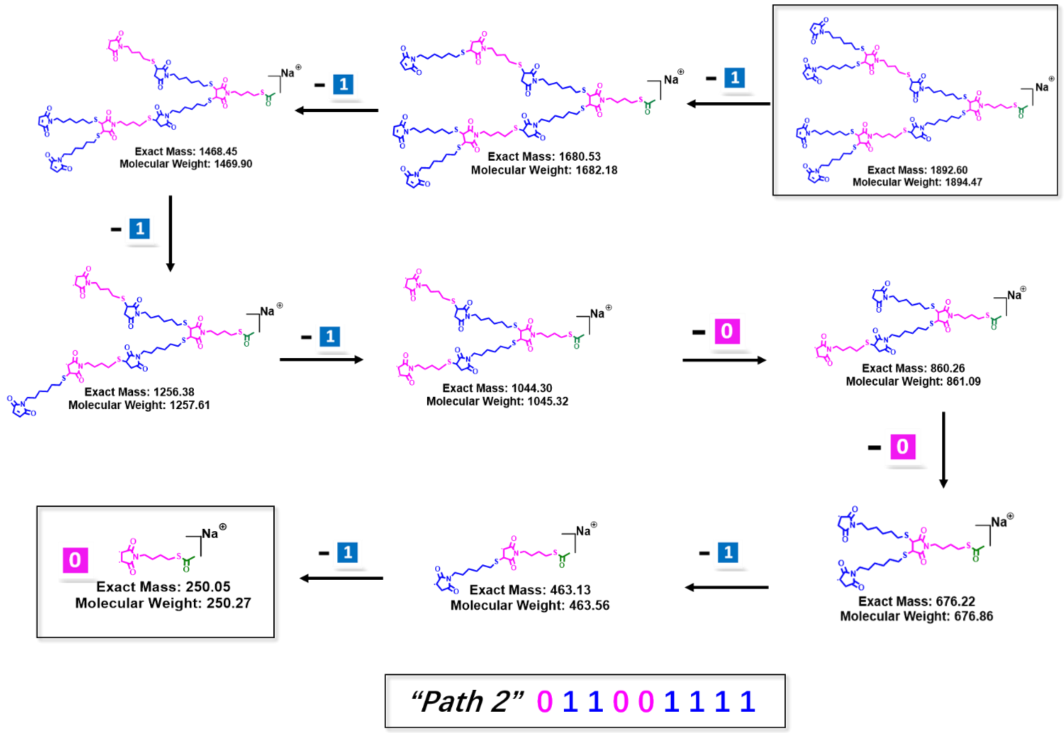

Supplementary Figure 45. Fragmentation pathway 2 of dendrimer **DN-011-G1**

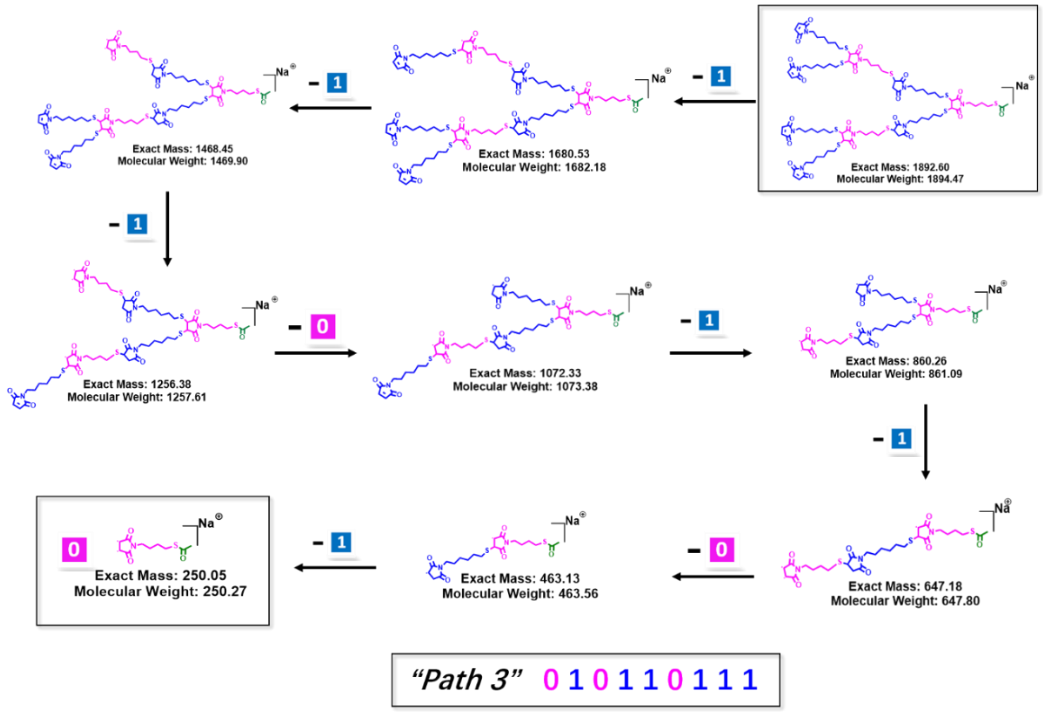

Supplementary Figure 46. Fragmentation pathway 3 of dendrimer **DN-011-G1**

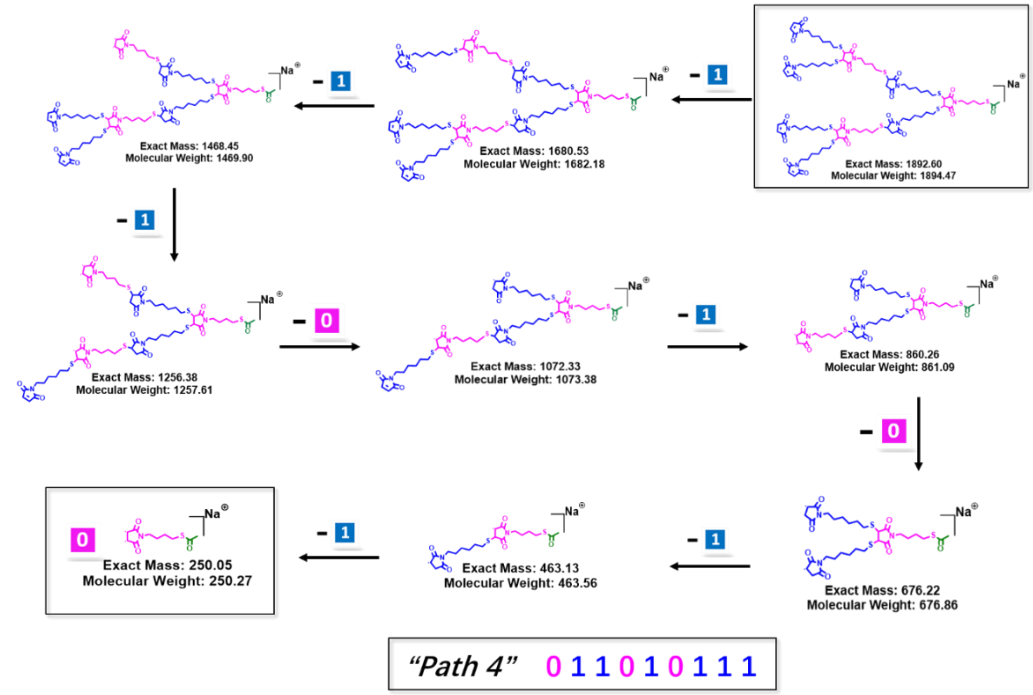

Supplementary Figure 47. Fragmentation pathway 4 of dendrimer **DN-011-G1**

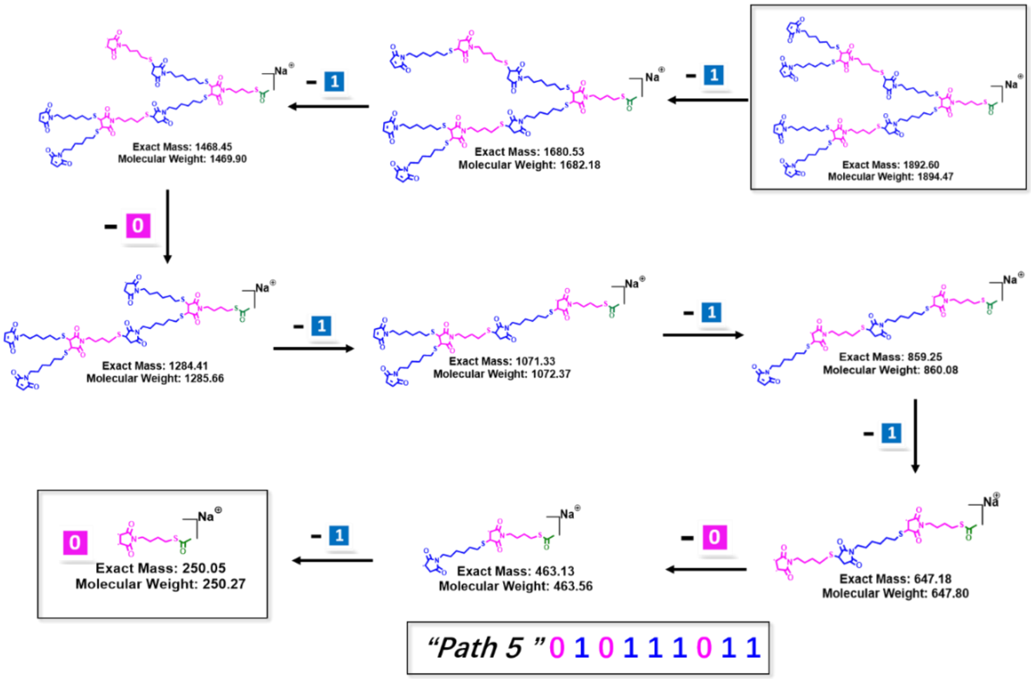

Supplementary Figure 48. Fragmentation pathway 5 of dendrimer **DN-011-G1**

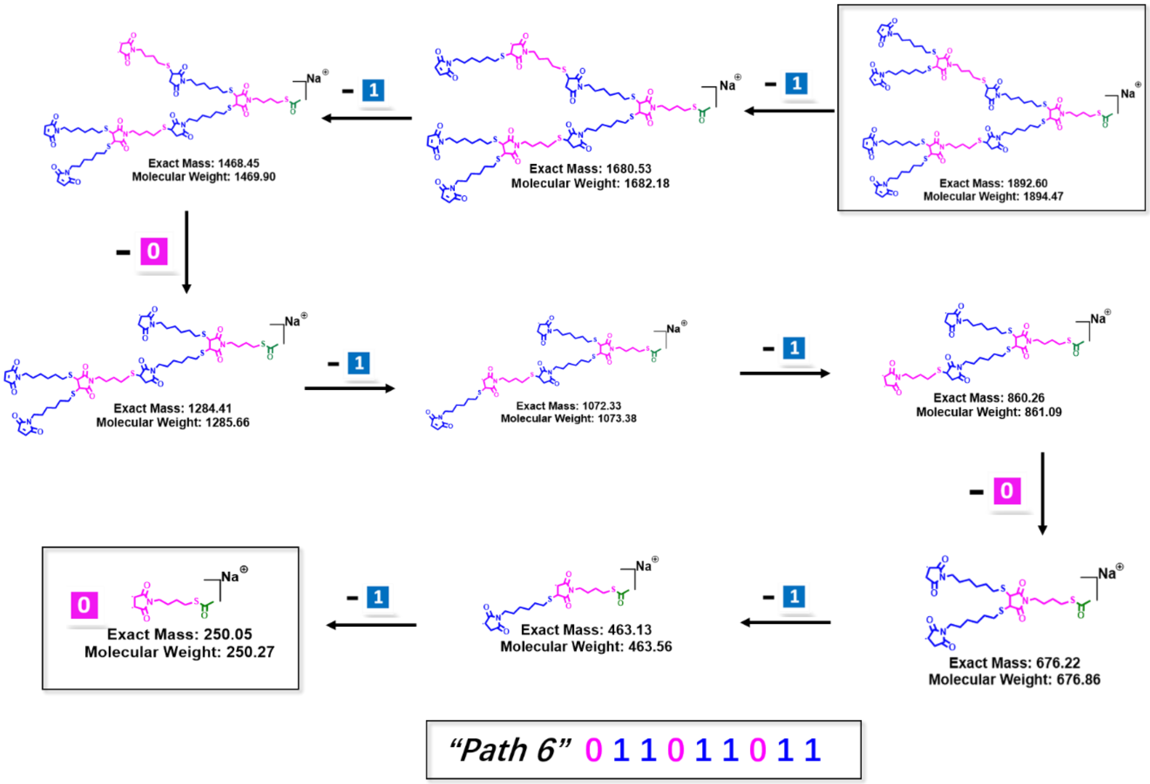

Supplementary Figure 49. Fragmentation pathway 6 of dendrimer **DN-011-G1**

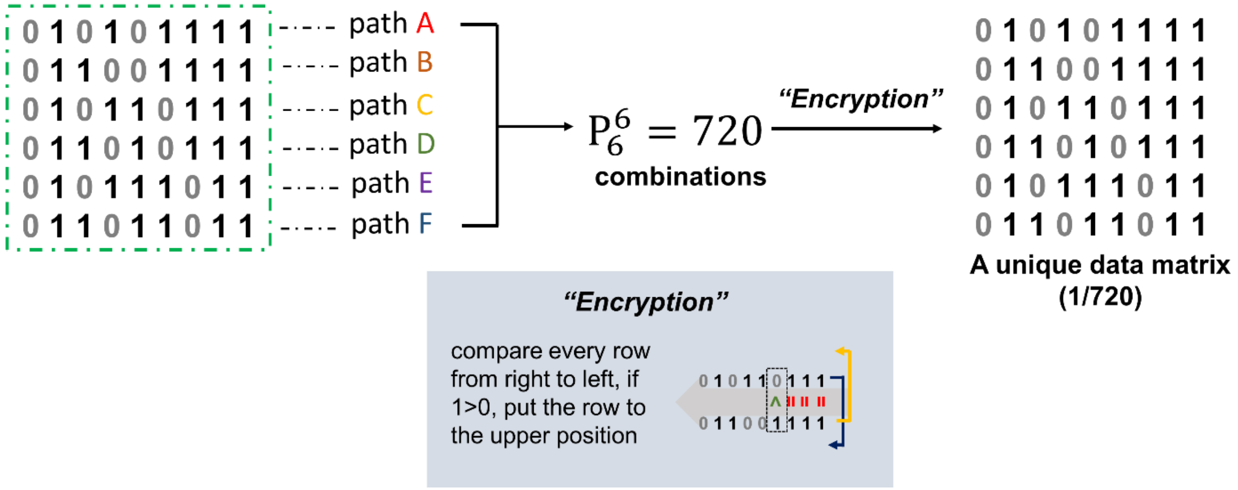

Supplementary Figure 50. The arrangement of the data matrix which generated from the MS/MS could be random. There were 720 combinations between these pathways from the top to the bottom. Therefore, an encryption was applied here, that is compare every row from right to left, if 1>0, put the row to the upper position. A unique data matrix could be obtained after the encryption. This sorting process could be considered as a second encryption, because the data matrix would be totally different if sorting with unauthorized regulation even correctly decoding the MS/MS.

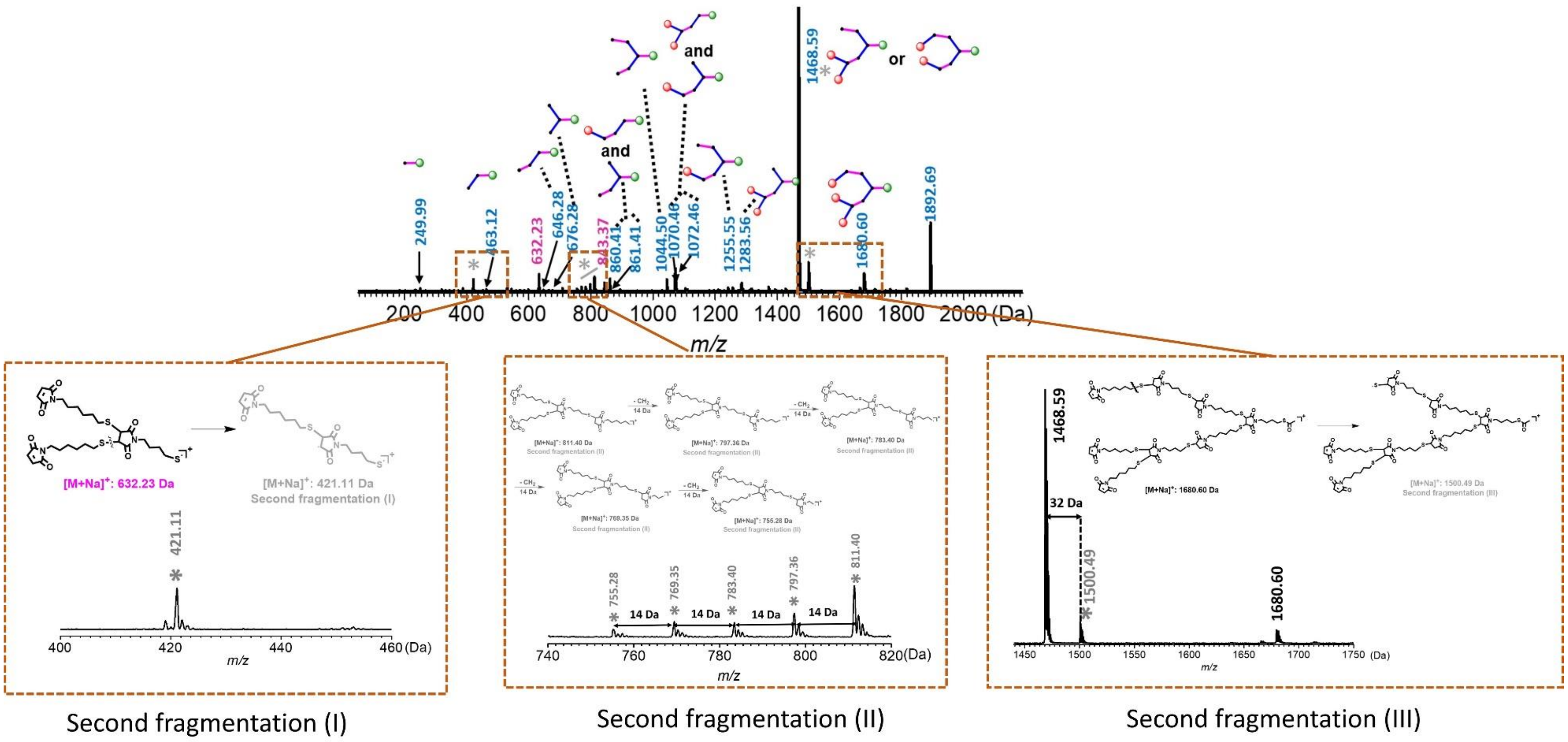

Supplementary Figure 51. Proposed secondary product ions formed during secondary dissociation reactions in MALDI TOF MS/MS of **DN-011-G1**

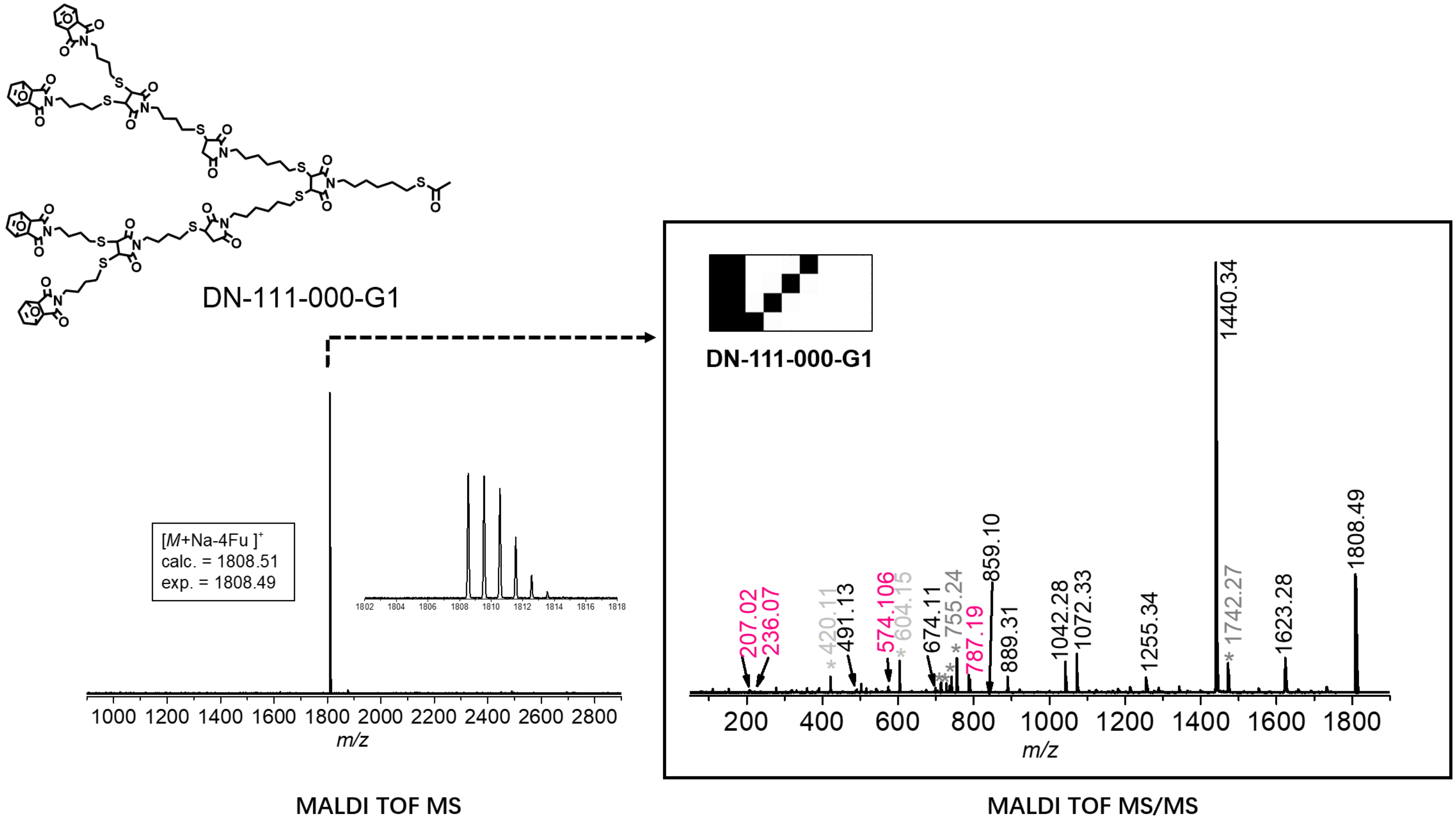

Supplementary Figure 52. Mass spectrometry analysis of the binary coded dendron **DN-111-000-G1**. MALDI TOF mass spectrum (left), showing the high monodispersity of the targeted species. MALDI TOF MS/MS mass spectrum (right), showing the assignment of dissociation fragments. * Internal fragments formed during secondary dissociation reactions.

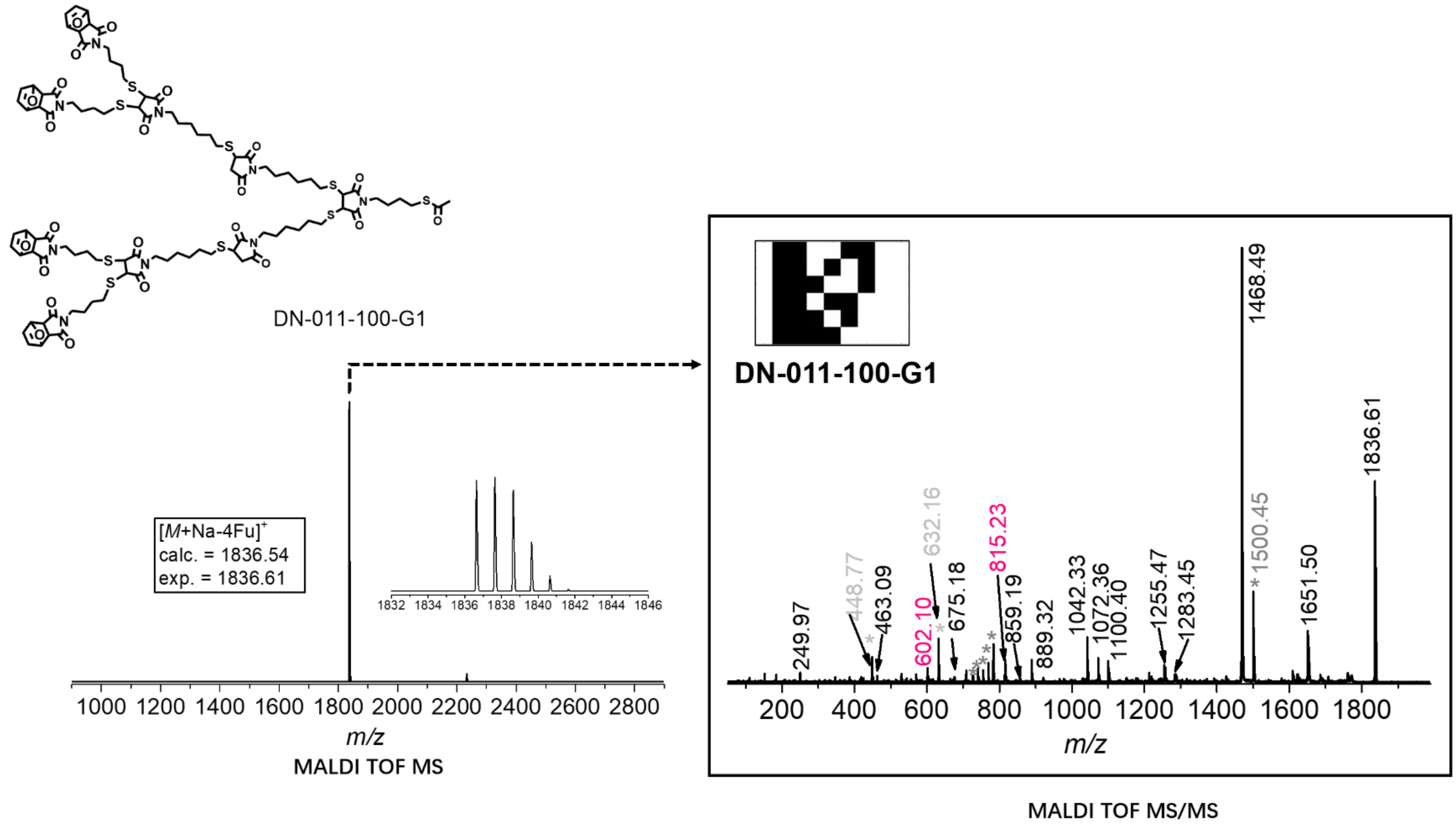

Supplementary Figure 53. Mass spectrometry analysis of the binary coded dendron **DN-011-100-G1**. MALDI TOF mass spectrum (left), showing the high monodispersity of the targeted species. MALDI TOF MS/MS mass spectrum (right), showing the assignment of dissociation fragments. * Internal fragments formed during secondary dissociation reactions.

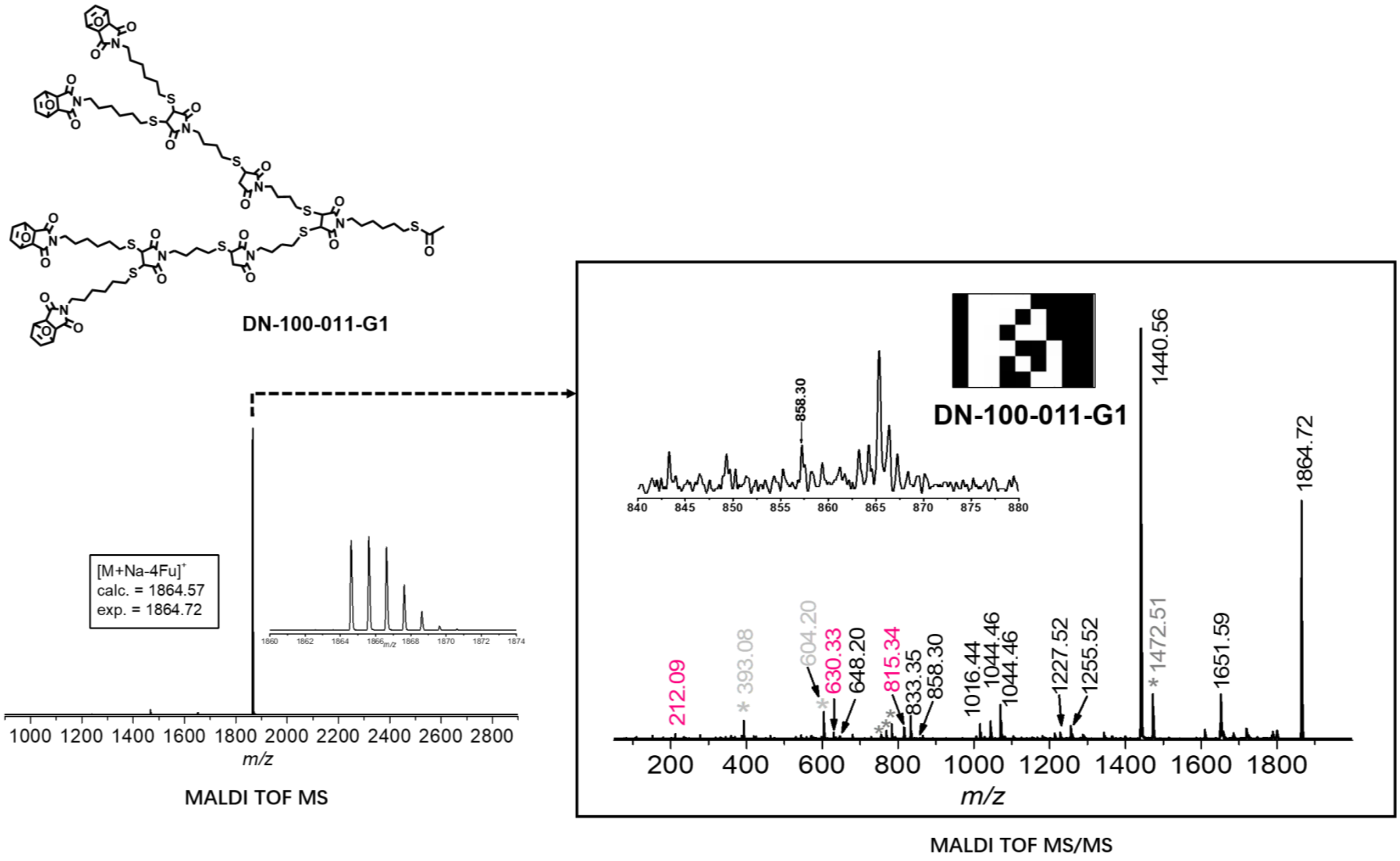

Supplementary Figure 54. Mass spectrometry analysis of the binary coded dendron **DN-100-011-G1**. MALDI TOF mass spectrum (left), showing the high monodispersity of the targeted species. MALDI TOF MS/MS mass spectrum (right), showing the assignment of dissociation fragments. * Internal fragments formed during secondary dissociation reactions.

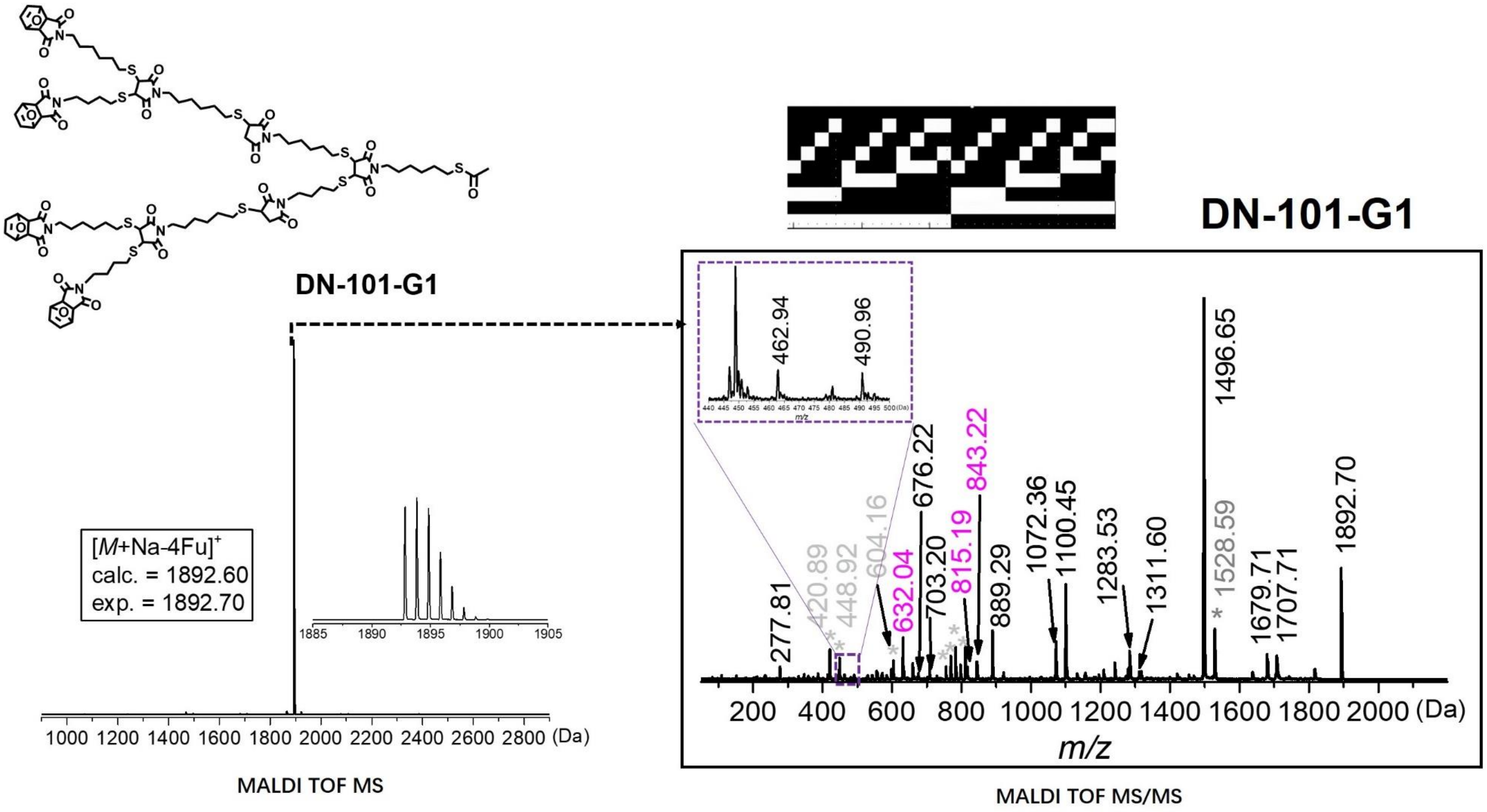

Supplementary Figure 55. Mass spectrometry analysis of the binary coded dendron **DN-101-G1**. MALDI TOF mass spectrum (left), showing the high monodispersity of the targeted species. MALDI TOF MS/MS mass spectrum (right), showing the assignment of dissociation fragments. * Internal fragments formed during secondary dissociation reactions.

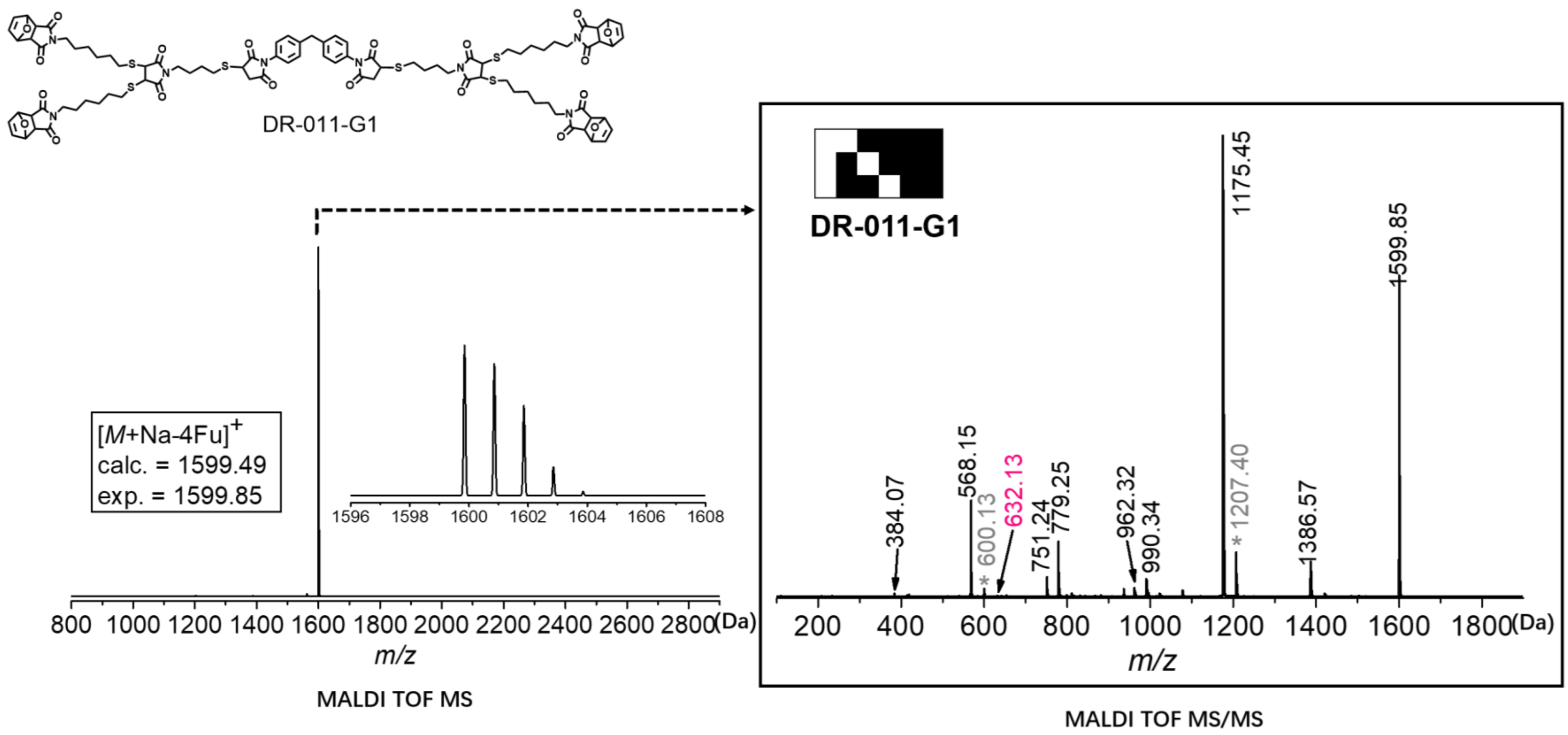

Supplementary Figure 56. Mass spectrometry analysis of the binary coded dendrimer **DR-011-G1**. MALDI TOF mass spectrum (left), showing the high monodispersity of the targeted species. MALDI TOF MS/MS mass spectrum (right), showing the assignment of dissociation fragments. * Internal fragments formed during secondary dissociation reactions.

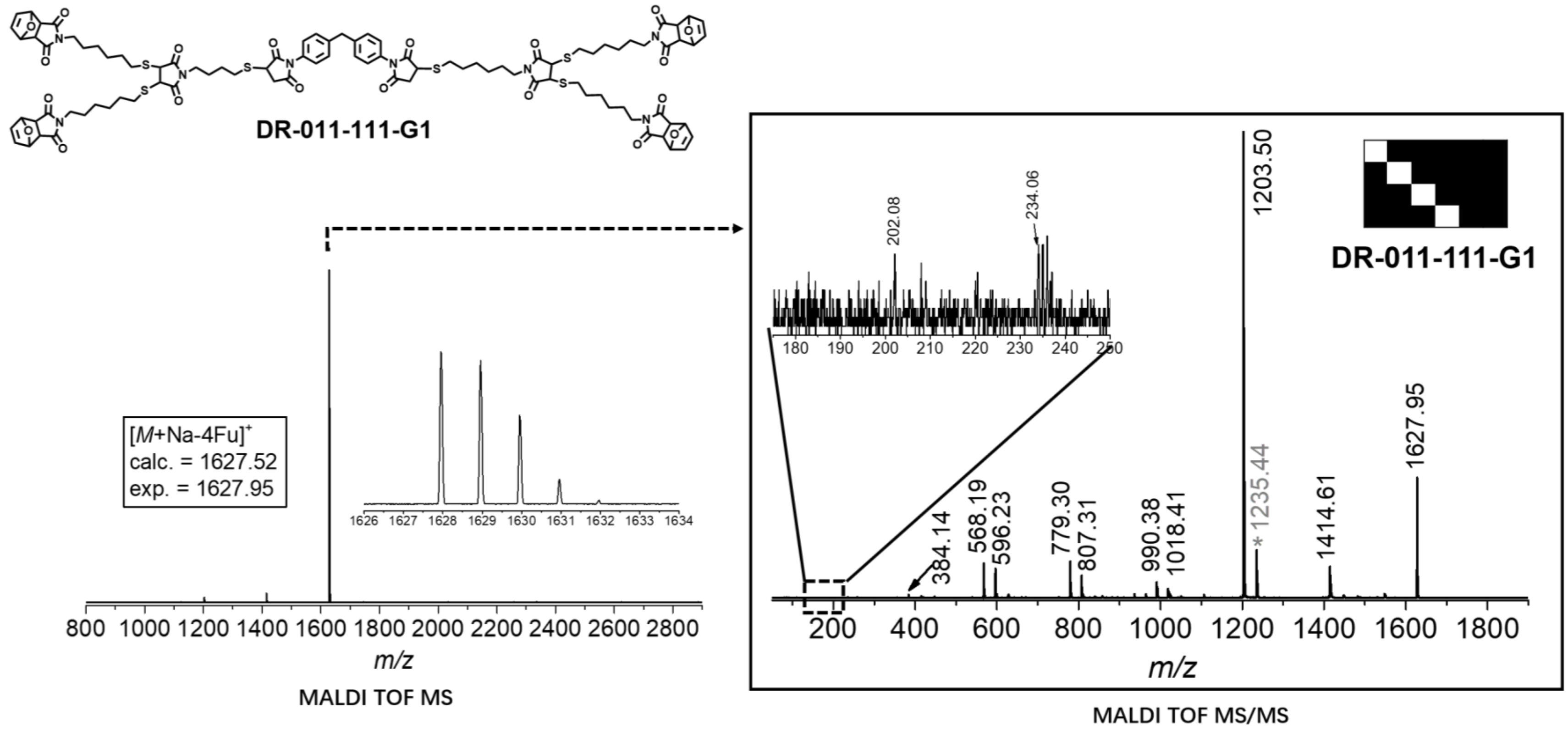

Supplementary Figure 57. Mass spectrometry analysis of the binary coded dendrimer **DR-011-111-G1**. MALDI TOF mass spectrum (left), showing the high monodispersity of the targeted species. MALDI TOF MS/MS mass spectrum (right), showing the assignment of dissociation fragments. * Internal fragments formed during secondary dissociation reactions.

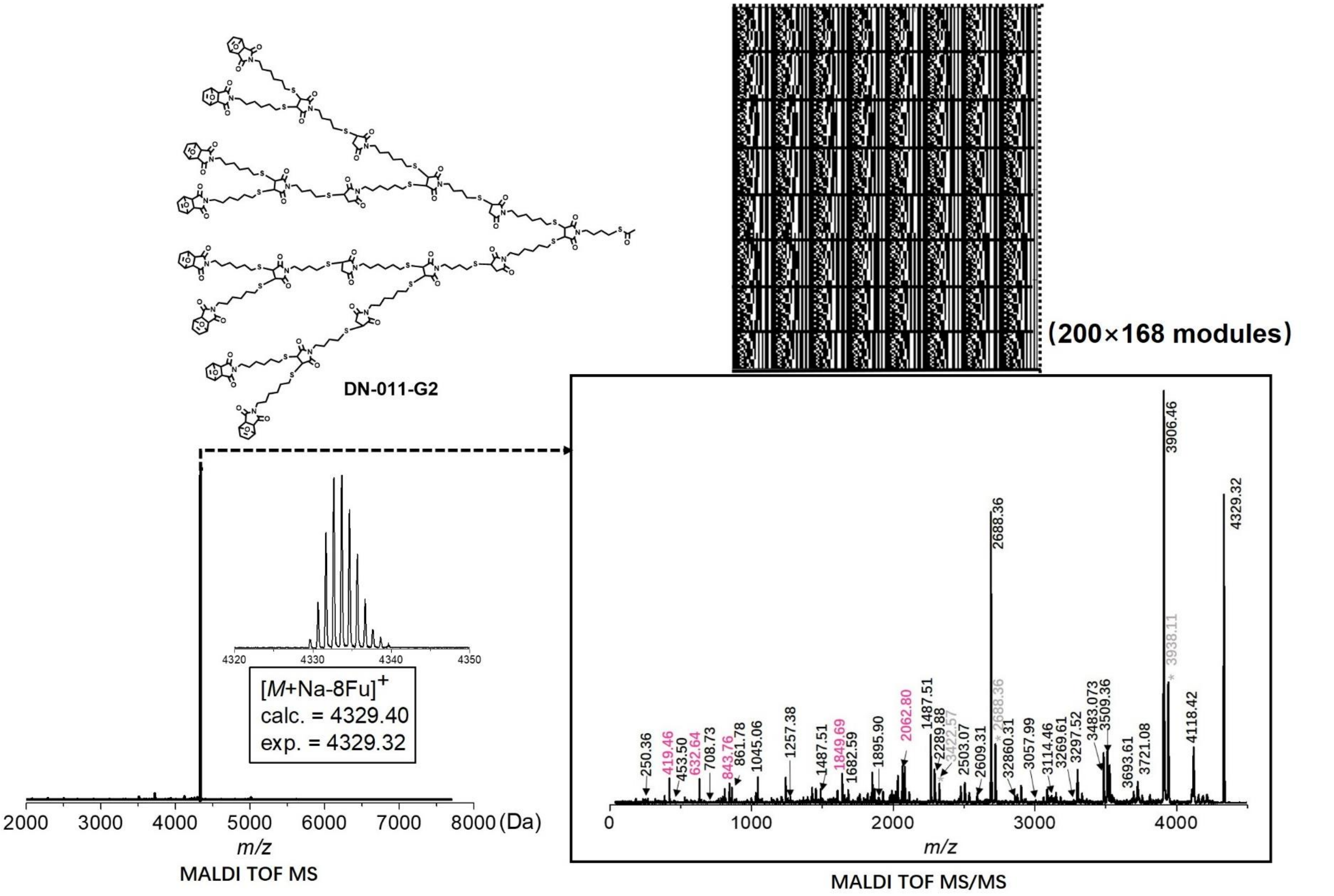

Supplementary Figure 58. Mass spectrometry analysis of the binary coded dendrimer **DN-011-G2**. MALDI TOF mass spectrum (left), showing the high monodispersity of the targeted species. MALDI TOF MS/MS mass spectrum (right), showing the assignment of dissociation fragments. * Internal fragments formed during secondary dissociation reactions.

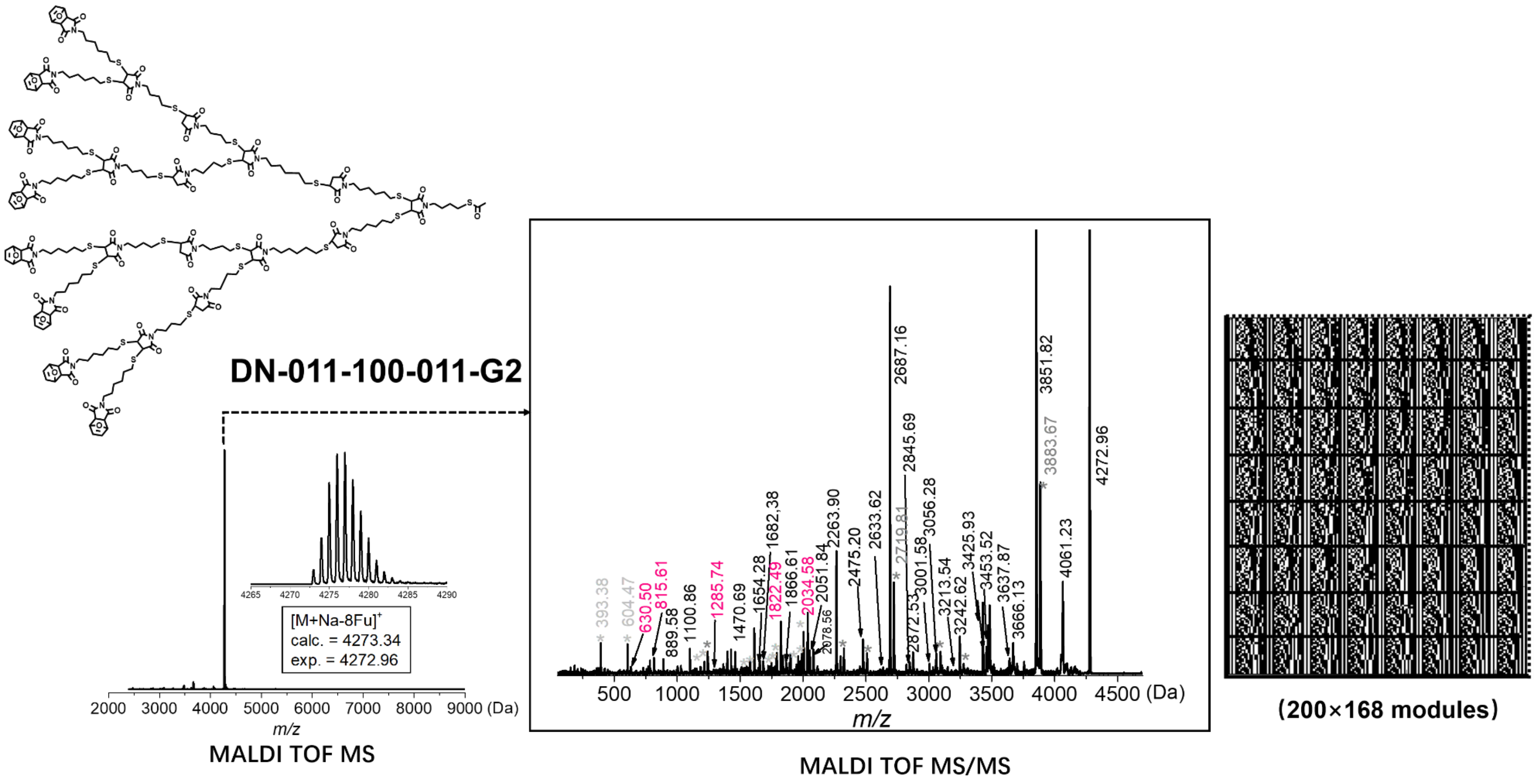

Supplementary Figure 59. Mass spectrometry analysis of the binary coded dendrimer **DN-011-100-011-G2**. MALDI TOF mass spectrum (left), showing the high monodispersity of the targeted species. MALDI TOF MS/MS mass spectrum (right), showing the assignment of dissociation fragments. * Internal fragments formed during secondary dissociation reactions.

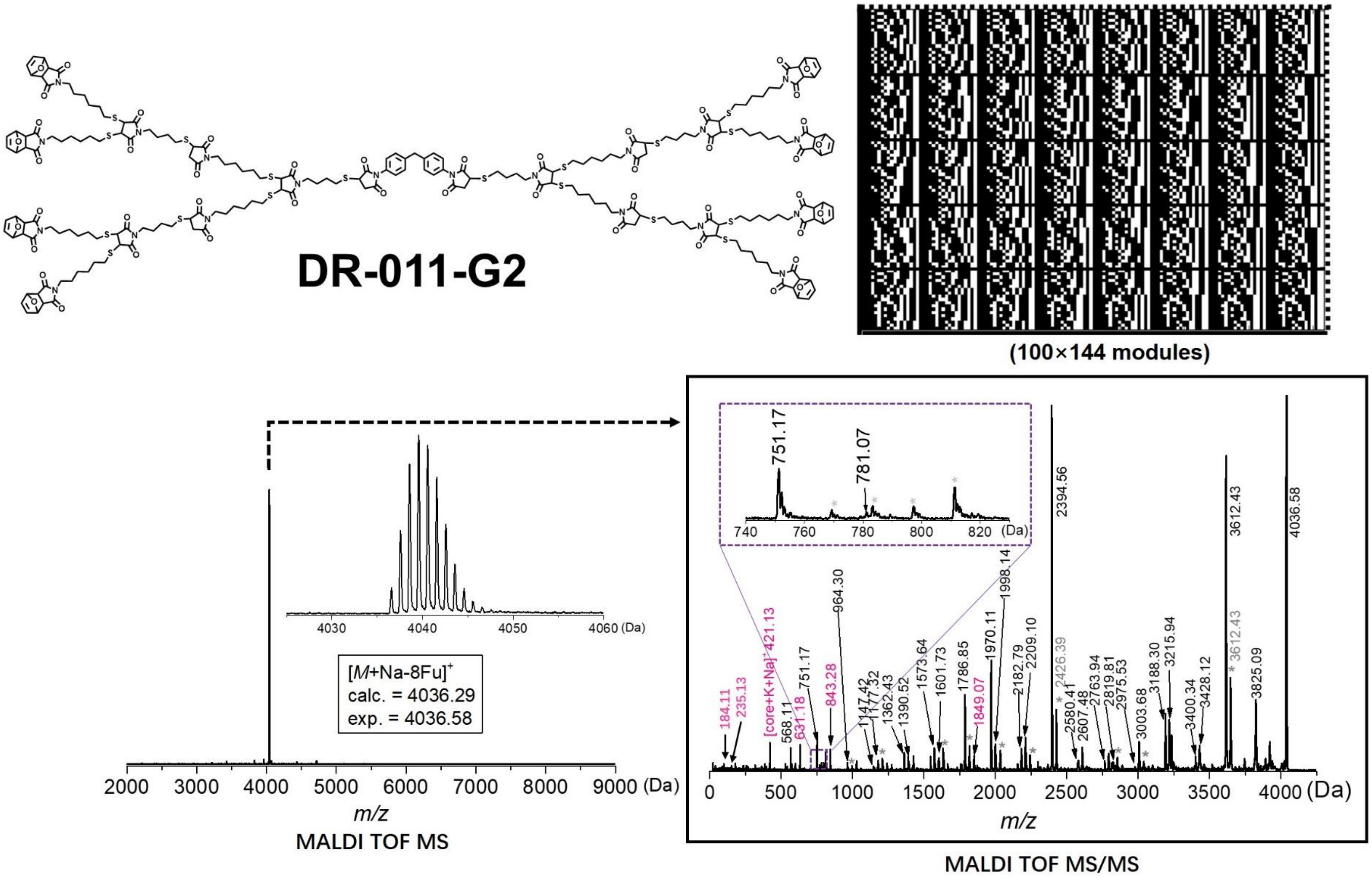

Supplementary Figure 60. Mass spectrometry analysis of the binary coded dendrimer **DR-011-G2**. MALDI TOF mass spectrum (left), showing the high monodispersity of the targeted species. MALDI TOF MS/MS mass spectrum (right), showing the assignment of dissociation fragments. * Internal fragments formed during secondary dissociation reactions.

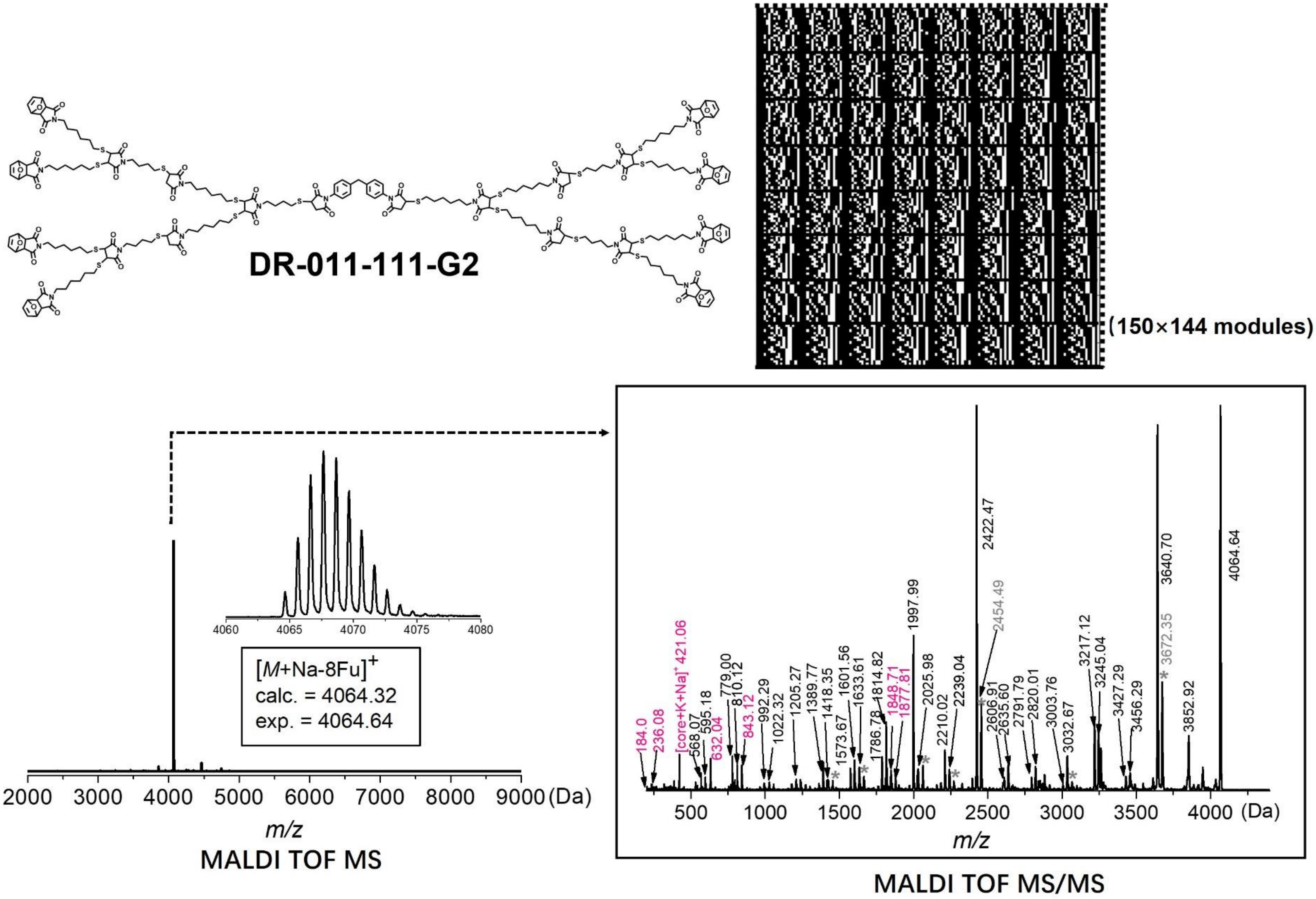

Supplementary Figure 61. Mass spectrometry analysis of the binary coded dendrimer **DR-011-111-G2**. MALDI TOF mass spectrum (left), showing the high monodispersity of the targeted species. MALDI TOF MS/MS mass spectrum (right), showing the assignment of dissociation fragments. * Internal fragments formed during secondary dissociation reactions.

a

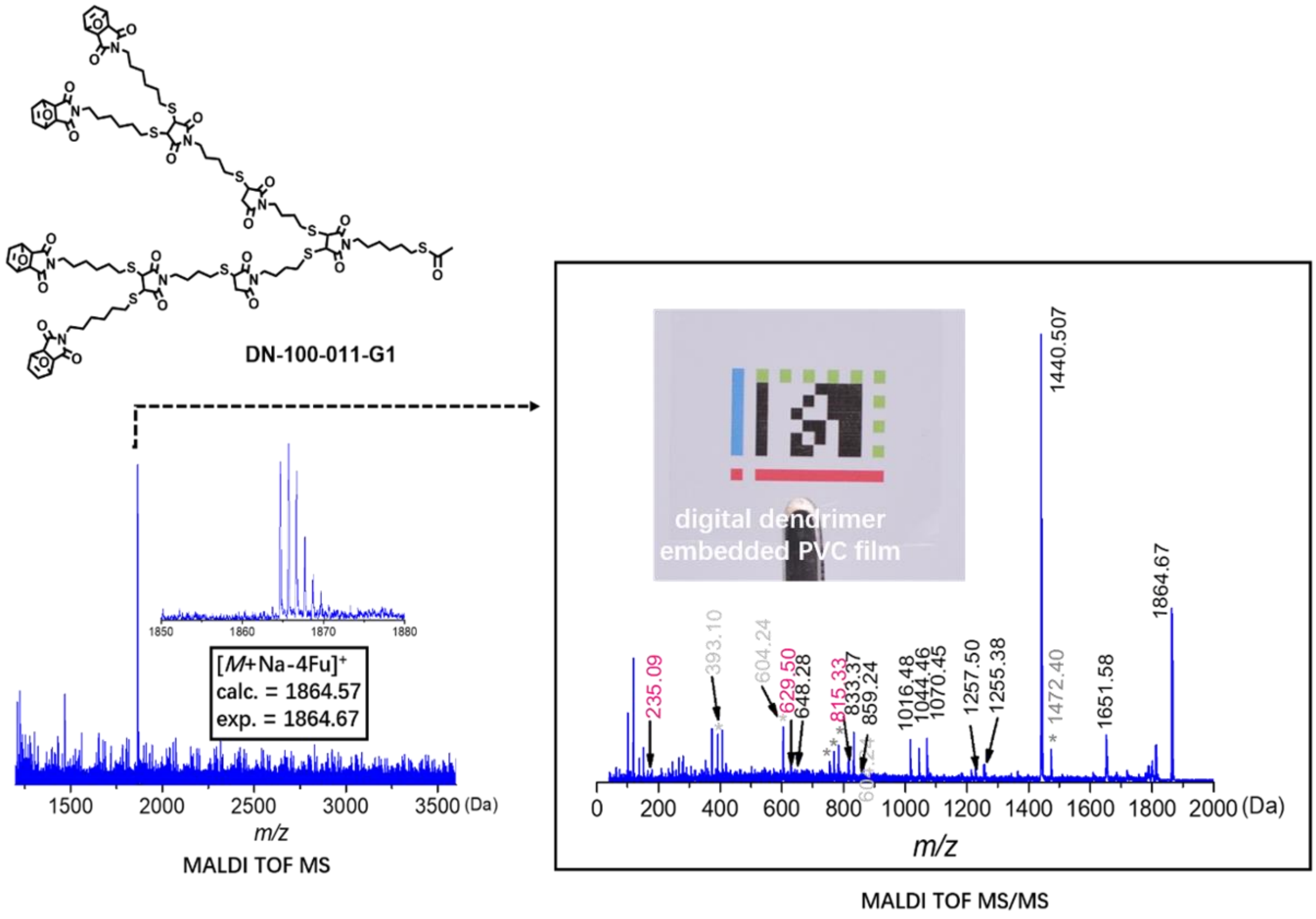
DN-100-011-G1
[M+Na-4Fu]+
calc. = 1864.57
exp. = 1864.67
1850
1860
1870
1880
1500
2000
2500
3000
3500 (Da)
m/z
MALDI TOF MS
digital dendrimer
embedded PVC film
1440.507
1864.67
235.09
393.10
604.24
629.50
648.28
815.33
833.37
859.24
1016.48
1044.46
1070.45
1257.50
1255.38
1472.40
1651.58
0
200
400
600
800
1000
1200
1400
1600
1800
2000 (Da)
m/z
MALDI TOF MS/MS

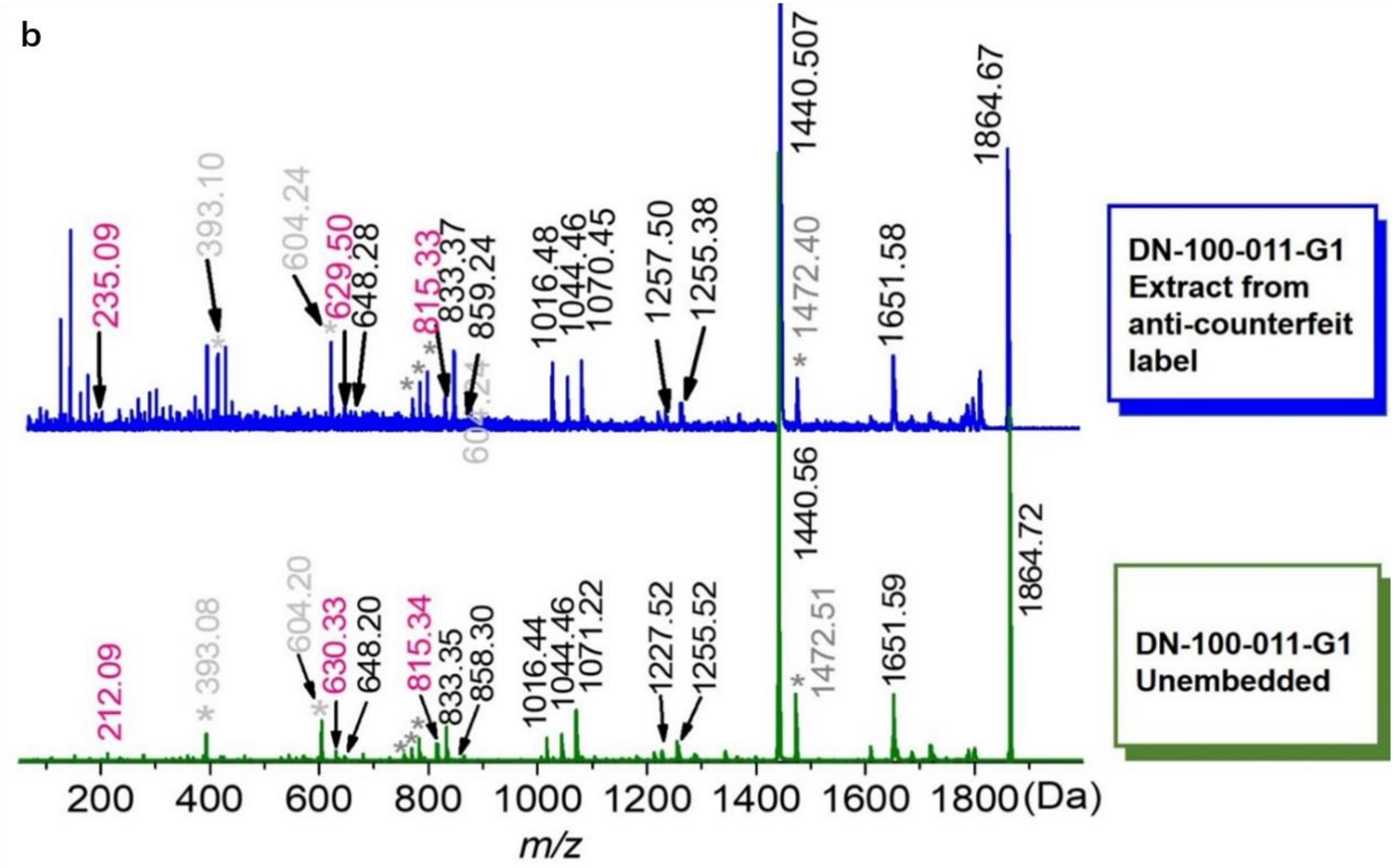

Supplementary Figure 62. (**a**) Mass spectrometry analysis of the binary coded dendron **DN-100-011-G1** that extracted from the anti-counterfeit label**.** (**b**) MALDI TOF MS/MS mass spectra comparison of the embedded (**up**) with unembedded (**down**) **DN-100-011-G1**. * Internal fragments formed during secondary dissociation reactions.

Supplementary References

(1) Wu, J. Y., Kuo, C. D., Chu, C. Y., Chen, M. S., Lin, J. H., Chen, Y. J., Liao, H. F., Synthesis of novel lipophilic N-substituted norcantharimide derivatives and evaluation of their anticancer activities. *Molecules* **19**, 6911 (2014)

(2) Ishihara, Y., Bazzi, H. S., Toader, V., Godin, F., Sleiman, H. F., Molecule-Responsive Block Copolymer Micelles. *Chemistry* **13**, 4560 (2007).

(3) Huang, Z. H., Zhao, J. F., Wang, Z. M., Meng, F. Y., Ding, K. S., Pan, X. Q., Zhou, N. C., Li, X. P., Zhang, Z. B., Zhu, X. L., Combining Orthogonal Chain-End Deprotections and Thiol-Maleimide Michael Coupling: Engineering Discrete Oligomers by an Iterative Growth Strategy. *Angew. Chem. Int. Ed.* **56**, 13612-13617 (2017).
