# Supplementary material for: Binary tree-inspired digital dendrimer
Source: Nat Commun. 2019 Apr 23;10:1918. doi: 10.1038/s41467-019-09957-6 (PMC6478934; doi:10.1038/s41467-019-09957-6)
Supplement: Supplementary file 3 — Description of Additional Supplementary Files [file 41467_2019_9957_MOESM3_ESM.docx]

Description of Additional Supplementary Files

**Supplementary Movie 1:** Movie for the item identification and traceability applications by using the digital dendrimer-based data matrix barcode.
